# Supplementary material for: Shifting tasks from pharmacy to non-pharmacy personnel for providing antiretroviral therapy to people living with HIV: a systematic review and meta-analysis
Source: BMJ Open. 2017 Aug 29;7(8):e015072. doi: 10.1136/bmjopen-2016-015072 (PMC5724105; doi:10.1136/bmjopen-2016-015072)
Supplement: Supplementary file 1 [file bmjopen-2016-015072supp001.pdf]

Downloaded from http://bmjopen.bmj.com/ on September 16, 2016 - Published by group.bmj.com

BMJ Open

The effects of shifting tasks from pharmacy to non-pharmacy personnel for providing antiretroviral therapy to people living with HIV: a systematic review protocol

Nyanyiwe M Mbeye,[1,2] Tamara Kredo,[1] Charles S Wiysonge[1,2]

To cite: Mbeye NM, Kredo T, Wiysonge CS. The effects of shifting tasks from pharmacy to non-pharmacy personnel for providing antiretroviral therapy to people living with HIV: a systematic review protocol. *BMJ Open* 2016;**6**: e008195. doi:10.1136/bmjopen-2015-008195

▸ Prepublication history for this paper is available online. To view these files please visit the journal online (http://dx.doi.org/10.1136/bmjopen-2015-008195).

Received 15 March 2015
Revised 16 November 2015
Accepted 17 November 2015

CrossMark

[1]Cochrane South Africa, South African Medical Research Council, Tygerberg, South Africa
[2]Faculty of Medicine and Health Sciences, Centre for Evidence-based Health Care, Stellenbosch University, Cape Town, South Africa

Correspondence to
Dr Nyanyiwe M Mbeye; nyanyiwembeye@gmail.com

ABSTRACT

Introduction: Shifting selected antiretroviral therapy (ART) tasks from specialised healthcare workers to those with shorter or less formal training has been implemented in resource-limited settings to alleviate critical shortages of human resources for health. However, the specifics of shifting ART dispensing from pharmacy to non-pharmacy personnel have not been addressed in a systematic review, although this can potentially increase access to ART. We will assess the effects of shifting dispensing and distribution of ART and adherence assessment from pharmacy to non-pharmacy personnel in low and middle-income countries.

Methods and analysis: We will search PubMed, CENTRAL, EMBASE, WHO Global Health Library and relevant grey literature for eligible controlled trials. Two authors will screen the search output, select eligible studies, assess risk of bias and extract data from included studies, resolving discrepancies by discussion and consensus. We will perform meta-analysis using both fixed and random effects models, investigate clinical and statistical heterogeneity, and assess our confidence in the overall evidence using standard Cochrane methods, including GRADE.

Ethics and dissemination: Only secondary data will be included in this review and ethics approval is not required. We will disseminate the review findings in various scientific fora, including peer-reviewed journals. The findings may help to inform policy makers in defining the scope of work of healthcare workers, and global recommendations for shifting the dispensing and distribution of ART from pharmacy to non-pharmacy personnel.

Trial registration number: CRD42015017034.

Strengths and limitations of this study

- To our knowledge, this is the first published protocol of a systematic review that will investigate the effects of task shifting from pharmacy to non-pharmacy personnel for dispensing or distributing antiretroviral therapy to patients living with HIV.
- The protocol was written according to the PRISMA-P (Preferred Reporting Items for Systematic Review and Meta-Analysis Protocols) recommendations.
- The review findings may help to inform antiretroviral therapy guidelines by the WHO.
- The possible weakness of the planned review would be the limitations of included studies, for example, high risk of bias and heterogeneity of settings, designs and effects.

INTRODUCTION

Description of the condition

By March 2015, 15 million (40.7%) of the estimated 36.9 million people living with HIV (PLHIV) globally were receiving antiretroviral therapy (ART).[1] Combination ART is effective for reducing HIV related morbidity and mortality as well as preventing HIV transmission.[2] Initiating ART early in the course of HIV infection has been associated with better health outcomes, both at patient and population levels.[3 4] Scale-up of ART in low and middle income countries has averted more than 5 million deaths; however, bottlenecks preventing universal access to ART still exist. One challenge is the critical shortage of human resources for health (HRH), including for the delivery of essential HIV related pharmacy services.

The WHO recommends a minimum of one pharmacist per 2300 population, but most countries in low-resource settings such as sub-Saharan Africa have not yet met this target.[5] In addition to the absolute shortage, it is likely that there is an uneven distribution of pharmacists in such settings, as is the case with other specialist healthcare workers who

Downloaded from http://bmjopen.bmj.com/ on September 16, 2016 - Published by group.bmj.com

tend to concentrate in urban areas and the private sector, further aggravating the HRH shortage.[6] For instance in South Africa, which is home to the largest number of PLHIV in any country in the world, in 2010 only 24% of registered pharmacists worked in the public sector where 80% of the population received care.[7]

Description of the intervention

Studies and programme reports indicate that involvement of pharmacy personnel in HIV care results in improved patient outcomes. For instance, in the USA, the use of a multidisciplinary team approach with pharmacists assuming a central role in ART initiation, dispensing and adherence counselling improved treatment outcomes such as viral load, patient retention and medication adherence.[8]

The work of pharmacists includes supply management, dispensing and distributing medications, promoting adherence, identifying and preventing potential medication-related issues, and monitoring and reporting adverse events. In some settings, programmes have implemented alternative models of pharmacy services that shift selected tasks from pharmacy to non-pharmacy personnel. Such alternative models could potentially increase the number of health workers involved in ART distribution, adherence counselling and patient education, free more time for pharmacy personnel, support the integration of ART in primary care settings, minimise the number of facility visits for ART collection, and reduce pharmacy queue waiting times for patients.[9]

However, the specifics of shifting ART related tasks from pharmacy to non-pharmacy personnel have not yet been addressed in a systematic review. We therefore plan to synthesise the evidence for task shifting in pharmacy services, where non-pharmacy personnel undertake ART dispensing and distribution and medication adherence counselling. For this systematic review, pharmacy personnel will include both pharmacists and pharmacy technicians. Pharmacy technicians constitute an important part of the pharmacy workforce in low and middle-income countries: a survey of 26 low and middle-income countries in 2011 revealed that pharmacy technicians constitute 10% (Nigeria) to 70% (Pakistan) of the pharmacy workforce.[10]

How the intervention might work

Within the last decade, several high HIV burden countries adopted task shifting strategies where nurses and non-physician clinicians initiate and maintain ART.[11] Although this has undeniably expanded access to ART, it is also increasingly essential that long facility waiting times and frequent facility visits to collect ART are addressed to alleviate the burden of care, both for patients and healthcare providers.[12 13]

Recent studies in Uganda, Kenya and Mozambique have shown positive outcomes when non-health professionals (lay people) delivered ART at the community level.[14] In Mozambique the use of PLHIV for distributing ART, monitoring adherence, reporting outcomes and referring sick patients to health facilities yielded a retention rate of 97.5% among stable patients on ART.[14] In a cluster randomised trial in Uganda, the use of trained community health workers produced comparable results with facility-based ART programme in terms of patient retention, viral load suppression and mortality rate.[15] Similar findings were also obtained in Kenya and Uganda when lay providers were engaged in ART delivery.[15 16]

Task shifting has therefore been seen as an achievable solution to the critical human resource shortages for scale-up of ART.[17] While it is imperative to increase the rate of recruitment and training of health workers as well as improve working conditions to reduce attrition and emigration, the HIV pandemic requires a more urgent measure to address the critical skills shortage.[18] Such measures may include shifting selected tasks (including dispensing and distributing ART and adherence counselling) from pharmacy to non-pharmacy personnel. The task shifting could free time for pharmacy personnel to focus on more technical functions such as supply management and pharmacovigilance.

Why this review is important

Previous systematic reviews of task shifting for increasing ART access focused on clinical services where nurses and non-clinician physicians provide care.[11] Dependence on and shortages of pharmacists are also key constraints on ART expansion, but the specifics of task shifting for ART dispensing or distribution from pharmacy to non-pharmacy personnel have not been reviewed systematically. We will systematically review the scientific literature and assess the efficacy and safety of task shifting models that use non-pharmacy personnel in dispensing or distributing ART and assessing adherence to treatment of HIV infection.

OBJECTIVE

The aim of this review is to evaluate the efficacy and safety of shifting dispensing and distribution of ART as well as assessment of adherence from pharmacy to non-pharmacy personnel.

METHODS

This review protocol has been registered in the PROSPERO International Prospective Register of Systematic Reviews (http://www.crd.york.ac.uk/PROSPERO), registration number CRD42015017034.

Criteria for considering studies for this review

Types of studies

We will include randomised controlled trials (RCTs) and non-RCTs, irrespective of whether allocation to interventions occurred at the individual or cluster level.

Types of participants

Participants will be PLHIV receiving ART.

Downloaded from http://bmjopen.bmj.com/ on September 16, 2016 - Published by group.bmj.com

Types of interventions

We will include studies that evaluate the shifting of selected tasks from pharmacy personnel to non-pharmacy personnel. The selected tasks include dispensing and distribution of ART and adherence assessment. Pharmacy personnel will include both pharmacists and pharmacy technicians. Non-pharmacy personnel may include (but are not limited to) nurses, non-physician clinicians, and lay providers such as patient peer groups, community volunteers, PLHIV and community health committees.

Types of outcome measures

Primary outcomes

The primary outcome for this review is risk of death.

Secondary outcomes

Our secondary outcome measures include:

- Virological suppression
- Number of all-cause sick visits made to the health facility, including for adverse events
- Loss to follow-up
- Adherence to ART (as measured within the study, eg, pill counts, recall methods, digital methods)
- Acceptability to pharmacy personnel, non-pharmacy personnel and patients
- Harm, including error rates.

Search methods for identification of studies

We will perform a comprehensive and exhaustive search of electronic databases and conference proceedings in an attempt to identify all relevant studies available by the search date, regardless of language of publication or publication status (published, unpublished, in press or in progress).

Databases of peer-reviewed literature

We will search the following electronic databases, from 1 January 1996 to the search date:

- Cochrane Central Register of Controlled Trials (CENTRAL)
- Excerpta Medica Database (EMBASE)
- PubMed
- ISI Web of Science (Science Citation index)
- WHO Global Health Library, which includes references from AIM (AFRO), LILACS (AMRO/PAHO), IMEMR (EMRO), IMSEAR (SEARO) and WPRIM (WPRO).

Along with appropriate Medical Subject Heading (MeSH) terms and relevant keywords, we will use the Cochrane Highly Sensitive Search Strategy for identifying reports of randomised controlled trials in MEDLINE,[19] and the Cochrane validated strategies for identifying references relevant to HIV infection and AIDS. To identify other study designs, the RCT string will be omitted. The search strategy will be iterative in that references of included studies will be searched for additional references. See table 1 for our provisional search strategy for electronic databases.

Conference databases

We will search conference abstract archives on the web sites of the Conference on Retroviruses and Opportunistic Infections (CROI), the International AIDS Conference (IAC) and the International AIDS Society Conference on HIV Pathogenesis, Treatment and Prevention (IAS), for all available abstracts presented at these conferences from 1996 to the search date.

Searching other resources

We will also search the references of relevant articles as well as the WHO International Clinical Trials Registry Platform (ICTRP) and Clinicaltrials.gov. We will contact relevant experts or organisations who may be aware of additional studies in this field.

Data collection and analysis

We will base the methodology for data collection and analysis on the guidance provided in the Cochrane Handbook of Systematic Reviews of Interventions.[19]

Selection of studies for inclusion

Two authors will read and assess the abstracts of identified publications for potentially eligible studies. We will obtain full text articles for all abstracts judged by at least one of the two authors, to be potentially eligible. Two authors will independently inspect these potentially eligible publications to establish the relevance of the article to the review according to the pre-specified criteria regarding study design, participants, interventions and outcome measures.

Data extraction and management

Two authors will independently extract data into a pre-piloted data extraction form. The following characteristics will be extracted from each included study:

Study details: Complete citations of publications associated with the study, start and end dates, location, study design characteristics, type of facility involved, investigators, funding sources, recruitment, method of randomisation, sequence generation, method of allocation concealment, blinding of participants and personnel, blinding of outcome assessment, length of follow-up, losses to follow-up, withdrawals or drop-outs and other relevant details.

Details of the intervention: training of the cadre of health workers who were dispensing or distributing ART, what training or other support or supervision they received and other relevant details.

Details of participants: Trial inclusion and exclusion criteria, numbers of participants entering the trial, sex, clinical staging, CD4 count and other pertinent details.

Outcome details: Definitions of outcomes, details of how outcomes were assessed, numerators and denominators associated with each outcome, completeness of outcome data, effect estimates reported and other relevant outcome information.

Downloaded from http://bmjopen.bmj.com/ on September 16, 2016 - Published by group.bmj.com

Table 1 Proposed search strategy for electronic databases

ID	Search terms
PubMed	
#1	(task*[tiab] OR task-shifting[tiab] OR referr*[tiab] OR referral and consultation[mh] OR role*[tiab]) AND (health personnel [mh] OR doctor[tiab] OR doctors[tiab] OR clinician[tiab] OR clinicians[tiab] OR physician[tiab] OR physicians[tiab] OR "healthcare provider"[tiab] OR "healthcare providers"[tiab] OR "health care provider"[tiab] OR "health care providers"[tiab] OR pharmac*[tiab] OR apothecar*[tiab] OR chemist*[tiab] OR dispensar*[tiab])
#2	randomized controlled trial[pt] OR controlled clinical trial[pt] OR randomized controlled trials[MeSH] OR random allocation[MeSH] OR double-blind method[MeSH] OR single-blind method[MeSH] OR clinical trial[pt] OR clinical trials [MeSH] OR ("clinical trial"[tw]) OR ((singl*[tw] OR doubl*[tw] OR trebl*[tw] OR tripl*[tw]) AND (mask*[tw] OR blind*[tw])) OR random*[tw] OR research design[mh:noexp] OR prospective studies[MeSH] OR control*[tw] OR volunteer*[tw]) OR observational[tw] OR non-random*[tw] OR nonrandom*[tw] OR before after study[tw] OR time series[tw] OR cohort*[tw] OR cross-section*[tw] OR prospective*[tw] OR retrospective*[tw] OR research design[mh:noexp] OR follow-up studies [MeSH] OR longitud*[tw] OR evaluat*[tiab] OR pre-post[tw] OR (pre-test[tw] AND post-test[tw]) NOT (animals[MeSH] NOT human[MeSH])
#3	(HIV Infections[MeSH] OR HIV[MeSH] OR hiv[tiab] OR hiv-1[tiab] OR hiv-2*[tiab] OR hiv1[tiab] OR hiv2[tiab] OR hiv infect*[tiab] OR human immunodeficiency virus[tiab]OR human immune deficiency virus[tiab] OR human immuno-deficiency virus[tiab] OR human immune-deficiency virus[tiab] OR ((human immun*) AND(deficiency virus[tiab])) OR acquired immunodeficiency syndromes[tiab] OR acquired immune deficiency syndrome[tiab] OR acquired immuno-deficiency syndrome[tiab] OR acquired immune-deficiency syndrome[tiab] OR ((acquired immun*) AND (deficiency syndrome[tiab])) or "sexually transmitted diseases, viral"[mh]) OR HIV[tiab] OR HIV/AIDS[tiab] OR HIV-infected[tiab] OR HIV[title] OR HIV/AIDS[title] OR HIV-infected[title]
#4	(HAART[tiab] OR ART[tiab] OR cART[tiab] OR antiretroviral[tiab] OR anti-retroviral[tiab] OR anti-viral[tiab] OR antiviral [tiab] OR "Antiretroviral Therapy, Highly Active"[Mesh])
**#5**	#1 AND #2 AND #3 AND #4
Scopus	
	(HIV OR HIV/AIDS OR AIDS OR "HUMAN IMMUNODEFICIENCY" OR "ACQUIRED IMMUNODEFICIENCY)" AND TITLE-ABS-KEY (TASK-SHIFTING OR TASKSHIFTING OR (TASK* AND SHIFT*) OR TASK* OR (REFERR* AND (NURSE* OR PHARMAC*))) AND TITLE-ABS-KEY (ANTIRETROVIRAL OR ANTI-RETROVIRAL OR ART OR CART OR HAART) AND TITLE-ABS-KEY (RANDOM* OR RANDOMIZED OR RANDOMISED OR TRIAL OR COHORT* OR GROUP* OR COMPAR* OR OBSERVATIONAL OR PROSPECTIVE* OR RETROSPECTIVE* OR "SYSTEMATIC REVIEW" OR "META-ANALYSIS)"
Web of Science	
	(TS=(HIV OR HIV/AIDS OR AIDS OR "HUMAN IMMUNODEFICIENCY" OR "ACQUIRED IMMUNODEFICIENCY)" AND TS=(TASK-SHIFTING OR TASKSHIFTING OR (TASK* AND SHIFT*) OR TASK* OR (REFERR* AND (NURSE* OR PHARMAC*))) AND TS=(ANTIRETROVIRAL OR ANTI-RETROVIRAL OR ART OR CART OR HAART) AND TS= (RANDOM* OR RANDOMIZED OR RANDOMISED OR TRIAL OR COHORT* OR GROUP* OR COMPAR* OR OBSERVATIONAL OR PROSPECTIVE* OR RETROSPECTIVE* OR "SYSTEMATIC REVIEW" OR "META-ANALYSIS)")
OR	
	(TI=(HIV OR HIV/AIDS OR AIDS OR "HUMAN IMMUNODEFICIENCY" OR "ACQUIRED IMMUNODEFICIENCY)" AND TI=(TASK-SHIFTING OR TASKSHIFTING OR (TASK* AND SHIFT*) OR TASK* OR (REFERR* AND (NURSE* OR PHARMAC*))) AND TI=(ANTIRETROVIRAL OR ANTI-RETROVIRAL OR ART OR cART OR HAART) AND TI= (RANDOM* OR RANDOMIZED OR RANDOMISED OR TRIAL OR COHORT* OR GROUP* OR COMPAR* OR OBSERVATIONAL OR PROSPECTIVE* OR RETROSPECTIVE* OR "SYSTEMATIC REVIEW" OR "META-ANALYSIS)")
CENTRAL	
	HIV* OR HIV-1* OR HIV-2* OR HIV1 OR HIV2 OR HIV INFECT* OR HUMAN IMMUNODEFICIENCY VIRUS OR HUMAN IMMUNEDEFICIENCY VIRUS OR HUMAN IMMUNE-DEFICIENCY VIRUS OR HUMAN IMMUNO-DEFICIENCY VIRUS OR HUMAN IMMUN* DEFICIENCY VIRUS OR ACQUIRED IMMUNODEFICIENCY SYNDROME OR ACQUIRED IMMUNEDEFICIENCY SYNDROME OR ACQUIRED IMMUNO-DEFICIENCY SYNDROME OR ACQUIRED IMMUNE-DEFICIENCY SYNDROME OR ACQUIRED IMMUN* DEFICIENCY SYNDROME in Title, Abstract, Keywords and (TASK-SHIFTING OR TASKSHIFTING OR (TASK* AND SHIFT*) OR TASK* OR (REFERR* AND (NURSE* OR PHARMAC*))) in Title, Abstract, Keywords and ANTIRETROVIRAL OR ANTI-RETROVIRAL OR ART OR cART OR HAART in Title, Abstract, Keywords
WHO Global Health Library	
	(TASK-SHIFTING OR TASKSHIFTING OR (TASK* AND SHIFT*) OR TASK* OR (REFERR* AND (NURSE* OR PHARMAC*)) AND (HIV* OR human immunodeficiency) AND (antiretroviral OR anti-retroviral))) OR (HIV AND task-shifting) OR (HIV* AND task* AND shift*)

Assessment of risk of bias in included studies

We will assess the risk of bias in RCTs using the Cochrane risk of bias assessment tool for randomised studies.[19] For non-RCT studies, we will use the Cochrane Risk of Bias Assessment Tool for Non-Randomised Studies of Interventions (ACROBAT-NRSI).[20]

Downloaded from http://bmjopen.bmj.com/ on September 16, 2016 - Published by group.bmj.com

We will resolve any disagreements between the authors conducting duplicate independent screening of search outputs, assessments of study eligibility, extraction of data, and risk of bias assessment by discussion and consensus. Should this fail to resolve the differences, a third author will arbitrate.

Measures of effect

We will calculate and report risk ratios for dichotomous and time-to-event data and mean differences for continuous data with their 95% CIs.

Unit of analysis issues

The unit of analysis will be the individual study participant. Cluster-randomised trials will be included in meta-analyses only after adjustments are made for design effect. Design effects for cluster-randomised studies will be corrected by using standard procedures, using the formula: design effect=$1+(m-1)r$, where m is the average cluster size and r is the intra-cluster correlation coefficient.

Dealing with missing data

We will contact study authors if it is necessary to obtain data missing from published reports.

Assessment of heterogeneity

We will examine statistical heterogeneity between study results using the χ^2 test of homogeneity, with a significance α-level of 0.1. In addition, we will use the I^2 statistic to measure the amount of heterogeneity among the trials in each analysis. If we identify significant heterogeneity (ie, $p<0.1$), we will explore it by prespecified subgroup analysis. If heterogeneity persists, we will perform sensitivity analyses, report results separately and propose reasons for the observed heterogeneity.

Assessment of reporting biases

If any meta-analysis in our review includes 10 or more studies, we will assess the potential for publication bias using a funnel plot.[21] We will attempt to minimise the potential for publication bias through a comprehensive search of published and unpublished literature.

Data synthesis

We will conduct meta-analysis, if appropriate, using the Cochrane Review Manager software (RevMan [Computer program] The Nordic Cochrane Centre, *The Cochrane Collaboration, Copenhagen*, 2015; Version 5.3). If we find no significant statistical heterogeneity of effects, we will use the fixed effect method of meta-analysis. Otherwise, we will use the random effects model.

Subgroup analysis

In pooled results with significant statistical heterogeneity, we will explore the cause of the heterogeneity through subgroup analyses, with subgroups defined by type of intervention (eg, cadre of health provider), comparison group and region of study (eg, sub-Saharan Africa, Southeast Asia, etc).

Sensitivity analysis

We will conduct a sensitivity analysis to investigate the effect of excluding studies with high risk of bias, with a focus on bias introduced by inadequate allocation concealment, inadequate blinding of outcome assessment and substantial losses to follow-up.

Certainty of evidence

We will assess the certainty (or quality) of evidence using the Grading of Recommendations, Assessment, Development and Evaluation (GRADE) approach,[22] which defines the certainty of evidence for each outcome as "the extent of our confidence that the estimates of effect are correct".[19] The quality rating across studies has four levels: high, moderate, low and very low. Randomised trials are considered to be of high quality but can be downgraded for any of five reasons: risk of bias, indirectness of evidence, unexplained heterogeneity of effects, imprecision of effect estimates and high probability of publication bias. Similarly, observational studies are considered to be of low quality, but can be upgraded for any of three reasons. The quality level of a body of evidence can be increased if there is a large magnitude of effect, if all plausible confounding would reduce a demonstrated effect, and if there is a dose-response gradient.

Reporting of this review

The findings of this review will be presented in a number of ways. The study selection process will be summarised using a flow diagram, and if we identify 10 or more eligible studies, we will assess publication bias using funnel plots. Where appropriate, we will use risk of bias graphs, forest plots and GRADE summary of findings tables. The non-quantitative outcomes will be reported descriptively. We will provide tables of both included and excluded studies. We have prepared this protocol as recommended by the PRISMA-P (Preferred Reporting Items for Systematic Review and Meta-Analysis Protocols) guidelines[23] and will report the findings of the review as recommended by the PRISMA statement.[24]

ETHICS AND DISSEMINATION

Since systematic reviews do not directly involve human participants, they do not require ethical clearance.[25] We will provide the findings of this review to the WHO, with the hope that they may guide policy recommendations of this normative agent regarding the shifting of ART dispensing or distribution from pharmacy to non-pharmacy personnel. Although the majority of national programmes in low and middle-income countries have adopted task shifting in ART care at different levels,

Downloaded from http://bmjopen.bmj.com/ on September 16, 2016 - Published by group.bmj.com

there has been no global policy to guide the practice for task shifting from pharmacy to non-pharmacy personnel. We will also publish the findings of the systematic review in a peer-reviewed journal.

Twitter Follow Charles Wiysonge at @CharlesShey

Acknowledgements The authors acknowledge the Editor and the referees for critical and constructive comments on earlier versions of this manuscript. The authors would also like to acknowledge the various contributions of Eyerusalem Negussie.

Contributors NMM led the development of the protocol, wrote the first draft, coordinated and integrated comments from co-authors and approved the final version for publication. TK and CSW conceived the study, critically revised successive drafts of the manuscript, and approved the final version for publication. CSW is the guarantor of the manuscript.

Funding Funding for preparing this review was provided by the Department of HIV/AIDS at the WHO Headquarters in Geneva, Switzerland. The authors would like to thank Hacsi Horvath (Global Health Sciences, University of California, San Francisco, USA) for his assistance in developing the search strategy for electronic databases. Neither the authors' institutions nor the funder played a role in preparing the manuscript and the views expressed therein are solely those of the authors.

Competing interests None declared.

Provenance and peer review Not commissioned; externally peer reviewed.

Open Access This is an Open Access article distributed in accordance with the terms of the Creative Commons Attribution (CC BY 4.0) license, which permits others to distribute, remix, adapt and build upon this work, for commercial use, provided the original work is properly cited. See: http://creativecommons.org/licenses/by/4.0/

REFERENCES

1. UNAIDS. How AIDS changed everything—MDG 6: 15 years, 15 lessons of hope from the AIDS response. https//:www.unaids.org/en/resources/documents/2015/MDG6_15years-15lessonsfromtheAIDSresponse (accessed 22 Sep 2015).
2. Palella FJ, Delaney KM, Moorman AC, *et al*. Declining morbidity and mortality among patients with advanced human immunodeficiency virus infection. HIV Outpatient Study Investigators. *N Engl J Med* 1998;338:853–60.
3. Anglemyer A, Rutherford GW, Horvath T, *et al*. Antiretroviral therapy for prevention of HIV transmission in HIV-discordant couples. *Cochrane Database Syst Rev* 2013;4:CD009153.
4. Ford N, Calmy A, Mills EJ. The first decade of antiretroviral therapy in Africa. *Global Health* 2011;7:33.
5. King RC, Fomundam HN. Remodeling pharmaceutical care in sub-Saharan Africa (SSA) amidst human resources challenges and the HIV/AIDS pandemic. *Int J Health Plann Manage* 2010;25:30–48.
6. WHO. *Increasing access to health workers in remote and rural areas through improved retention: global policy recommendations*. Geneva, Switzerland: World Health Organisation, 2010.
7. SAPC. Pharmacy Human Resources data. https//:www.healthlink.org.za/healthstats/283/data (accessed 15 Aug 2015).
8. Saberi P, Dong BJ, Johnson MO, *et al*. The impact of HIV clinical pharmacists on HIV treatment outcomes: a systematic review. *Patient Prefer Adherence* 2012;6:297–322.
9. Tseng A, Foisy M, Hughes CA, *et al*. Role of pharmacist in caring for patients with HIV/AIDS: clinical practice guidelines. *Can J Hosp Pharm* 2012;65:125–45.
10. WHO. *The world of medicines situation 2011: pharmaceutical human resources*. Geneva, Switzerland: World Health Organisation, 2011.
11. Kredo T, Adeniyi FB, Bateganya M, *et al*. Task shifting from doctors to non-doctors for initiation and maintenance of antiretroviral therapy. *Cochrane Database Syst Rev* 2014;7:CD007331.
12. Kitahata MM, Koepsell TD, Deyo RA, *et al*. Physicians experience with the acquired immunodeficiency syndrome as a factor in patients' survival. *N Engl J Med* 1996;334:701–6.
13. Landon BE, Wilson IB, McInnes K, *et al*. Physician specialization and the quality of care for human immunodeficiency virus infection. *Arch Intern Med* 2005;165:1133–9.
14. Decroo T, Telfer B, Biot M, *et al*. Distribution of antiretroviral treatment through self-forming groups of patients in Tete province, Mozambique. *J Acquir Immune Defic Syndr* 2011;56:e39–44.
15. Kipp W, Konde-Lule J, Rubaale T, *et al*. Comparing antiretroviral treatment outcomes between a prospective community-based and hospital-based cohort of HIV patients in rural Uganda. *BMC Int Health Hum Rights* 2011;11(Suppl 2):S12.
16. Selke HM, Kimaiyo S, Sidle JE, *et al*. Task-shifting of antiretroviral delivery from health care workers to persons living with HIV/AIDS: clinical outcomes of a community-based program in Kenya. *J Acquir Immune Defic Syndr* 2010;55:483–90.
17. Callaghan M, Ford N, Schneider H. A systematic review of task-shifting for HIV treatment and care in Africa. *Hum Resour Health* 2010;8:8.
18. Laurant M, Reeves D, Hermens R, *et al*. Substitution of doctors by nurses in primary care. *Cochrane Database Syst Rev* 2005;CD001271.
19. Higgins JPT, Green S, eds. *Cochrane handbook for systematic reviews of interventions*. Wiley-Blackwell, 2008.
20. Cochrane Collaboration. A Cochrane Risk of Bias Assessment Tool: for Non-Randomized studied of Interventions (ACROBAT-NRSI). https://www.riskofbias.info/ (accessed 30 Oct 2015).
21. Egger M, Davey Smith G, Schneider M, *et al*. Bias in meta-analysis detected by a simple, graphical test. *BMJ* 1997;315:629–34.
22. Guyatt G, Oxman AD, Akl EA, *et al*. GRADE guidelines: 1. Introduction-GRADE evidence profiles and summary of findings tables. *J Clin Epidemiol* 2011;64:383–94.
23. Shamseer L, Moher D, Clarke M, *et al*; PRISMA-P Group. Preferred reporting items for systematic review and meta-analysis protocols (PRISMA-P). *BMJ* 2015;349:g7647.
24. Liberati A, Altman DG, Tetzlaff J, *et al*. The PRISMA Statement for reporting systematic reviews and meta-analyses of studies that evaluate healthcare interventions: explanation and elaboration. *BMJ* 2009;339:b2700.
25. Emanuel EJ, Wendler D, Killen J, *et al*. What makes clinical research in developing countries ethical? The benchmarks of ethical research. *J Infect Dis* 2004;189:930–7.

Downloaded from http://bmjopen.bmj.com/ on September 16, 2016 - Published by group.bmj.com

BMJ Open

The effects of shifting tasks from pharmacy to non-pharmacy personnel for providing antiretroviral therapy to people living with HIV: a systematic review protocol

Nyanyiwe M Mbeye, Tamara Kredo and Charles S Wiysonge

BMJ Open 2016 6:
doi: 10.1136/bmjopen-2015-008195

Updated information and services can be found at:
http://bmjopen.bmj.com/content/6/3/e008195

These include:

References This article cites 18 articles, 4 of which you can access for free at:
http://bmjopen.bmj.com/content/6/3/e008195#BIBL

Open Access This is an Open Access article distributed in accordance with the terms of the Creative Commons Attribution (CC BY 4.0) license, which permits others to distribute, remix, adapt and build upon this work, for commercial use, provided the original work is properly cited. See:
http://creativecommons.org/licenses/by/4.0/

Email alerting service Receive free email alerts when new articles cite this article. Sign up in the box at the top right corner of the online article.

Topic Collections Articles on similar topics can be found in the following collections

Evidence based practice (557)
Health services research (1106)
HIV AIDS (159)

Notes

To request permissions go to:
http://group.bmj.com/group/rights-licensing/permissions

To order reprints go to:
http://journals.bmj.com/cgi/reprintform

To subscribe to BMJ go to:
http://group.bmj.com/subscribe/
