# Supplementary material for: Shifting tasks from pharmacy to non-pharmacy personnel for providing antiretroviral therapy to people living with HIV: a systematic review and meta-analysis
Source: BMJ Open. 2017 Aug 29;7(8):e015072. doi: 10.1136/bmjopen-2016-015072 (PMC5724105; doi:10.1136/bmjopen-2016-015072)
Supplement: Author's manuscript [file bmjopen-2016-015072.draft_revisions.pdf]

BMJ Open

BMJ Open is committed to open peer review. As part of this commitment we make the peer review history of every article we publish publicly available.

When an article is published we post the peer reviewers' comments and the authors' responses online. We also post the versions of the paper that were used during peer review. These are the versions that the peer review comments apply to.

The versions of the paper that follow are the versions that were submitted during the peer review process. They are not the versions of record or the final published versions. They should not be cited or distributed as the published version of this manuscript.

BMJ Open is an open access journal and the full, final, typeset and author-corrected version of record of the manuscript is available on our site with no access controls, subscription charges or pay-per-view fees (http://bmjopen.bmj.com).

If you have any questions on BMJ Open's open peer review process please email editorial.bmjopen@bmj.com

BMJ Open

Shifting tasks from pharmacy to non-pharmacy personnel for providing antiretroviral therapy to people living with HIV: A systematic review and meta-analysis

Journal:	*BMJ Open*
Manuscript ID	bmjopen-2016-015072
Article Type:	Research
Date Submitted by the Author:	09-Nov-2016
Complete List of Authors:	MBEYE, NYANYIWE; Stellenbosch University, Centre for Evidence Based Health Care; College of Medicine, School of Public Health and Family Medicine<br>Adetokunboh, Olatunji; Stellenbosch University, Centre for Evidence Based Health Care; South African Medical Research Council, Cochrane South Africa<br>Negussie, Eyerusalem; World Health Organisation, HIV/AIDS<br>Kredo, Tamara; South African Medical Research Council, Cochrane South Africa; Stellenbosch University Faculty of Medicine and Health Sciences, Division of clinical Pharmacology<br>Wiysonge, Charles; Stellenbosch University, Centre for Evidence-Based Health Care; South African Medical Research Council, Cochrane South Africa
**Primary Subject Heading**:	Evidence based practice
Secondary Subject Heading:	Health services research, HIV/AIDS
Keywords:	Task shifting, HIV, antiretroviral therapy, dispensing, distribution, pharmacy personnel

SCHOLARONE™ Manuscripts

Shifting tasks from pharmacy to non-pharmacy personnel for providing antiretroviral therapy to people living with HIV: A systematic review and meta-analysis

Nyanyiwe M. Mbeye[1,2&5*], Olatunji Adetokunboh[1&2], Eyerusalem Negussie[3], Tamara Kredo[1&4], Charles S. Wiysonge[1&2],

[1] Cochrane South Africa, South African Medical Research Council, Cape Town, South Africa.

[2] Centre for Evidence-based Health Care, Faculty of Medicine and Health Sciences, Stellenbosch University, Cape Town, South Africa.

[3] Department of HIV/AIDS, World Health Organization, Geneva, Switzerland

[4] Division of Clinical Pharmacology, Faculty of Medicine and Health Sciences, Stellenbosch University, Cape Town, South Africa.

[5]College of Medicine, University of Malawi, Blantyre, Malawi.

***Correspondence author:** Dr Nyanyiwe M. Mbeye (nyanyiwembeye@gmail.com), Telephone Number: +265 888356338

For peer review only

Abstract

Objectives

Lay people or non-pharmacy health workers with training could dispense antiretroviral therapy (ART) in resource-constrained countries; freeing up time for pharmacists to focus on more technical tasks. We assessed the effectiveness of such task-shifting in low and middle-income countries.

Method

We conducted comprehensive searches of peer-reviewed and grey literature. Two authors independently screened search outputs, selected controlled trials, and extracted data; resolving discrepancies by consensus. We performed random-effects meta-analysis and assessed certainty of evidence using GRADE.

Results

Three studies with 1993 participants met the inclusion criteria, including two cluster trials conducted in Kenya and Uganda and an individually randomised trial conducted in Brazil. Pooling data we found that there may be no difference between pharmacy and non-pharmacy models of dispensing ART on mortality (RR 1.86, 95% confidence interval 0.44 to 7.95), virological failure (RR 0.92, 0.73 to 1.15) and loss to follow-up (RR 1.13, 0.68 to 1.91). We found some evidence that costs may be reduced for the patient and health system when task-shifting is undertaken. The overall quality of the evidence was low for all outcomes due to serious indirectness (trials compared complex interventions involving, but not limited to, pharmacy to non-pharmacy personnel) and imprecision (effects ranged from appreciable benefit to harm).

For peer review only

Conclusions

Health outcomes may be similar when ART dispensing or distribution is shifted from pharmacy to non-pharmacy personnel. However, the low-certainty evidence implies a high likelihood that further research may find the effects of the intervention to be substantially different from our findings. If resource-constrained countries decide to shift ART dispensing and distribution from pharmacy to non-pharmacy personnel, this should be accompanied by robust monitoring and impact evaluation.

Key words: Task-shifting, HIV, antiretroviral therapy, dispensing, distribution, pharmacy personnel.

Strengths and limitations

- To our knowledge, this is the first published systematic review reporting the effects of task shifting from pharmacy to non-pharmacy personnel for dispensing or distributing antiretroviral therapy to patients living with HIV.
- The review was written according to the PRISMA (Preferred Reporting Items for Systematic Review and Meta-Analysis) Recommendations for reporting systematic reviews
- The review findings may help to inform antiretroviral therapy guidelines by the WHO.
- The possible weakness of the planned review would be the limitations of included studies, for example, high risk of bias and heterogeneity of settings,

For peer review only

designs and effects.

Background

Description of the condition

By March 2015, 15 million (40.7%) of the estimated 36.9 million people living with HIV (PLHIV) globally, were receiving antiretroviral therapy (ART) in low and middle-income countries [1]. Combination ART is effective for reducing HIV-related morbidity and mortality as well as preventing HIV transmission [2]. Initiating ART early in the course of HIV infection has been associated with better health outcomes, both at patient and population levels [3 4]. Scale up of ART in low and middle-income countries has averted more than 5 million deaths; however, bottlenecks preventing universal access to ART still exist. One challenge is the critical shortage of human resources for health (HRH), including for delivery of essential pharmacy services.

As for pharmacists, although the World Health Organization (WHO) recommends a minimum of one pharmacist per 2300 population [5], most countries in low-resource

For peer review only - http://bmjopen.bmj.com/site/about/guidelines.xhtml

settings such as sub-Saharan Africa do not meet this target. In addition to the absolute shortage, it is likely that there is an uneven distribution of pharmacists in such settings, as is the case with other specialist healthcare workers who tend to concentrate in urban areas and the private sector, further aggravating the HRH shortage. For instance in South Africa, which is home to the largest number of PLHIV in any country worldwide, in 2010 only 24% of registered pharmacists worked in the public sector where 80% of the population received care [6].

Description of the intervention

Studies and programmes report that involvement of pharmacy personnel in HIV care results in improved patient outcomes [7]. For instance, in the USA, the use of a multidisciplinary team approach with pharmacists assuming a central role in the initiation, dispensing and adherence counselling improved treatment outcomes such as viral load, patient retention and medication adherence [8].

The work of pharmacists includes supply management, dispensing and distributing medications, promoting adherence, identifying and preventing potential medication-related issues (such as over dosage, sub-therapeutic dosage, adverse drug reactions, medication errors and untreated indications), and monitoring and reporting adverse drug events [9 10]. In some settings, programmes have implemented alternative models of pharmacy services that shift selected tasks from pharmacy to non-pharmacy personnel. Such alternative models could potentially increase the number of health workers involved in ART distribution, adherence counselling and patient education; free more time for pharmacy personnel; support the integration of ART in primary care settings; minimise the number of facility visits for ART collection; and, reduce pharmacy queue waiting times for patients.

For peer review only - http://bmjopen.bmj.com/site/about/guidelines.xhtml

The specifics of shifting ART-related tasks from pharmacy personnel have not been addressed in a systematic review. Available systematic reviews on task shifting focus on clinical services where nurses and non-physician clinicians provide care comparable to physicians [11]. We therefore synthesised the evidence for task shifting in pharmacy personnel services, where non-pharmacy personnel undertake ART distribution and medication adherence counselling. Table 1 provides definitions for the pharmacy functions distribution and dispensing as they have been used in this review.

Table 1: Definitions and descriptions of dispensing and distribution of medications

Term	Definition
Dispensing	• Dispensing is a controlled act that authorises one to select, prepare and provide stock medication that has been prescribed to a patient or client (or his/her representative) for administration at a later time [12].<br>• To dispense is to prepare and supply to a patient a course of therapy on the basis of a prescription [11].<br>• Dispensing is preparation and distribution of a course of therapy to a patient, with appropriate instructions based on a prescription [11].
Distribution	• At facility level, drug distribution mainly refers to drugs dispensed by licensed practitioners, such as nurses, doctors, pharmacists or pharmacy assistants and collected by a patient.<br>• At community level, drug distribution refers to trained lay people collecting pre-packed medications from the facility and delivering them to HIV patients in the community [12].

How task shifting using non-pharmacy personnel might work

Within the last decade, several high HIV burden countries adopted task shifting strategies where nurses and non-physician clinicians initiate and maintain ART. Although this has undeniably expanded access to ART, it is also increasingly essential

For peer review only

that long facility waiting times and frequent facility visits to collect ART are addressed to alleviate the burden of care, both for patients and healthcare providers [9] [10].

Recent studies in Uganda, Kenya and Mozambique have shown positive outcomes when non-health professionals (lay people) delivered ART at the community level [13]. In Mozambique, the use of PLHIV for distributing ART, monitoring adherence, reporting outcomes and referring sick patients to health facilities yielded a retention rate of 97.5% among stable patients on ART [13]. In a cluster randomised trial in Uganda, the use of community health workers produced comparable results with the facility-based ART programme in terms of patient retention, viral load suppression and mortality rate [14]. Similar findings were also obtained in Kenya and some other parts of Uganda when lay providers were engaged in ART delivery [14 15].

Task shifting has therefore been seen as an achievable solution to the critical human resource shortages affecting scale up of ART [16]. While it is imperative to increase the rate of recruitment and training of health workers as well as improve working conditions to reduce attrition and emigration, the HIV pandemic requires that all possible options are considered to address the critical skills shortage [17]. Such measures may include shifting selected tasks from pharmacy to non-pharmacy personnel, particularly for patients stable on their ART. Task-shifting allows more time for pharmacy personnel to focus on more technical functions such as supply management, pharmacovigilance and patient consultation.

Why this review is important

Dependence on and shortage of pharmacists are key constraints on ART expansion, but the specifics of task shifting for ART distribution from pharmacy to non-

For peer review only

pharmacy personnel have not been reviewed systematically. Previous systematic reviews of task shifting for increasing ART access focused on clinical services where nurses and non-physician clinicians provide care. We systematically reviewed the scientific literature and assessed the efficacy of task-shifting models that use non-pharmacy and pharmacy personnel in distributing ART and assessing adherence to treatment of HIV infection.

Objective

The aim of this review was to evaluate the efficacy and safety of shifting pharmacy related tasks, including ART distribution and adherence assessment, from pharmacy to non-pharmacy personnel.

Methods

The review title was registered in the PROSPERO International Prospective Register of Systematic reviews (http://www.crd.york.ac.uk/PROSPERO), and the protocol published in the BMJ Open [18].

For peer review only

Criteria for considering studies for this review

Types of studies: We included randomised controlled trials (RCTs) conducted in low and middle-income countries.

Types of participants*:* Eligible participants were people living with HIV receiving ART.

Types of interventions: We included studies that evaluated shifting of selected tasks from pharmacy to non-pharmacy personnel. The selected tasks include distribution of ART, and assessing adherence to ART shown in Table 1. Pharmacy personnel included pharmacists and pharmacy technicians, while non-pharmacy personnel included patient peer groups, community volunteers, people living with HIV, community health committees, nurses, physicians and non-physician clinicians.

Types of outcome measures*:* The primary outcome for this review is risk of death. Secondary outcomes include virological suppression; number of all-cause sick visits made to the health facility including adverse events; loss to follow-up; adherence to ART (as measured within the study, e.g. pill counts, recall methods, digital methods, self report); acceptability to pharmacy personnel, non-pharmacy personnel, patients; cost and harm including rates of errors.

Search methods for identification of studies

We performed a comprehensive search of electronic databases and conference proceedings to identify all relevant studies available up until 10 May 2016, regardless of language of publication or publication status (published, unpublished, in press and in progress). We searched the following electronic databases for studies indexed by the search date: Cochrane Central Register of Controlled Trials (CENTRAL),

For peer review only

Excerpta Medica Database (EMBASE), PubMed, ISI Web of Science (Science Citation index), and World Health Organization (WHO) Global Health Library. We used appropriate Medical Subject Heading (MeSH) terms, relevant keywords, and validated terms for identifying reports of randomised controlled trials [19]. We provide the details of the search strategy for each electronic database in Table 2. We also searched conference abstract archives on the websites of the Conference on Retroviruses and Opportunistic Infections (CROI), the International AIDS Conference (IAC), and the International AIDS Society Conference on HIV Pathogenesis, Treatment and Prevention (IAS), for all available abstracts presented at these conferences up to 10 May 2016. We contacted experts and organisations for additional studies in the field.

For peer review only

Table 2: Search strategy for electronic databases

ID	Search terms
**PubMed**	
#1	(task*[tiab] OR task-shifting[tiab] OR referr*[tiab] OR referral and consultation[mh] OR role*[tiab]) AND (health personnel[mh] OR doctor[tiab] OR doctors[tiab] OR clinician[tiab] OR clinicians[tiab] OR physician[tiab] OR physicians[tiab] OR "healthcare provider"[tiab] OR "healthcare providers"[tiab] OR "health care provider"[tiab] OR "health care providers"[tiab] OR pharmac*[tiab] OR apothecar*[tiab] OR chemist*[tiab] OR dispensar*[tiab])
#2	randomized controlled trial[pt] OR controlled clinical trial[pt] OR randomized controlled trials[MeSH] OR random allocation[MeSH] OR double-blind method[MeSH] OR single-blind method[MeSH] OR clinical trial[pt] OR clinical trials[MeSH] OR ("clinical trial"[tw]) OR ((singl*[tw] OR doubl*[tw] OR trebl*[tw] OR tripl*[tw]) AND (mask*[tw] OR blind*[tw])) OR random*[tw] OR research design[mh:noexp] OR prospective studies[MeSH] OR control*[tw] OR volunteer*[tw]) OR observational[tw] OR non-random*[tw] OR nonrandom*[tw] OR before after study[tw] OR time series[tw] OR cohort*[tw] OR cross-section*[tw] OR prospective*[tw] OR retrospective*[tw] OR research design[mh:noexp] OR follow-up studies[MeSH] OR longitud*[tw] OR evaluat*[tiab] OR pre-post[tw] OR (pre-test[tw] AND post-test[tw]) NOT (animals[MeSH] NOT human[MeSH])
#3	(HIV Infections[MeSH] OR HIV[MeSH] OR hiv[tiab] OR hiv-1[tiab] OR hiv-2*[tiab] OR hiv1[tiab] OR hiv2[tiab] OR hiv infect*[tiab] OR human immunodeficiency virus[tiab]OR human immune deficiency virus[tiab] OR

	human immuno-deficiency virus[tiab] OR human immune-deficiency virus[tiab] OR ((human immun*) AND(deficiency virus[tiab])) OR acquired immunodeficiency syndromes[tiab] OR acquired immune deficiency syndrome[tiab] OR acquired immuno-deficiency syndrome[tiab] OR acquired immune-deficiency syndrome[tiab] OR ((acquired immun*) AND (deficiency syndrome[tiab])) or “sexually transmitted diseases, viral”[mh]) OR HIV[tiab] OR HIV/AIDS[tiab] OR HIV-infected[tiab] OR HIV[title] OR HIV/AIDS[title] OR HIV-infected[title]
#4	(HAART[tiab] OR ART[tiab] OR cART[tiab] OR antiretroviral[tiab] OR anti-retroviral[tiab] OR anti-viral[tiab] OR antiviral[tiab] OR "Antiretroviral Therapy, Highly Active"[Mesh])
**#5**	#1 AND #2 AND #3 AND #4
**Scopus**	
	(HIV OR HIV/AIDS OR AIDS OR "HUMAN IMMUNODEFICIENCY" OR "ACQUIRED IMMUNODEFICIENCY") AND TITLE-ABS-KEY (TASK-SHIFTING OR TASKSHIFTING OR (TASK* AND SHIFT*) OR TASK* OR (REFERR* AND (NURSE* OR PHARMAC*))) AND TITLE-ABS-KEY (ANTIRETROVIRAL OR ANTI-RETROVIRAL OR ART OR CART OR HAART) AND TITLE-ABS-KEY (RANDOM* OR RANDOMIZED OR RANDOMISED OR TRIAL OR COHORT* OR GROUP* OR COMPAR* OR OBSERVATIONAL OR PROSPECTIVE* OR RETROSPECTIVE* OR "SYSTEMATIC REVIEW" OR "META-ANALYSIS")
**Web of Science**	
	(TS=(HIV OR HIV/AIDS OR AIDS OR "HUMAN IMMUNODEFICIENCY" OR "ACQUIRED IMMUNODEFICIENCY") AND TS=(TASK-SHIFTING OR TASKSHIFTING OR (TASK* AND SHIFT*) OR TASK* OR (REFERR* AND (NURSE* OR PHARMAC*))) AND TS=(ANTIRETROVIRAL OR ANTI-RETROVIRAL OR ART OR CART OR HAART) AND TS=(RANDOM* OR RANDOMIZED OR RANDOMISED OR TRIAL OR COHORT* OR GROUP* OR COMPAR* OR OBSERVATIONAL OR PROSPECTIVE* OR RETROSPECTIVE* OR "SYSTEMATIC REVIEW" OR "META-ANALYSIS"))<br><br>**OR**<br><br>(TI=(HIV OR HIV/AIDS OR AIDS OR "HUMAN IMMUNODEFICIENCY" OR "ACQUIRED IMMUNODEFICIENCY") AND TI=(TASK-SHIFTING OR TASKSHIFTING OR (TASK* AND SHIFT*) OR TASK* OR (REFERR* AND (NURSE* OR PHARMAC*))) AND TI=(ANTIRETROVIRAL OR ANTI-RETROVIRAL OR ART OR cART OR HAART) AND TI=(RANDOM* OR RANDOMIZED OR RANDOMISED OR TRIAL OR COHORT* OR GROUP* OR COMPAR* OR OBSERVATIONAL OR PROSPECTIVE* OR RETROSPECTIVE* OR "SYSTEMATIC REVIEW" OR "META-ANALYSIS"))

For peer review only

CENTRAL	
	HIV* OR HIV-1* OR HIV-2* OR HIV1 OR HIV2 OR HIV INFECT* OR HUMAN IMMUNODEFICIENCY VIRUS OR HUMAN IMMUNEDEFICIENCY VIRUS OR HUMAN IMMUNE-DEFICIENCY VIRUS OR HUMAN IMMUNO-DEFICIENCY VIRUS OR HUMAN IMMUN* DEFICIENCY VIRUS OR ACQUIRED IMMUNODEFICIENCY SYNDROME OR ACQUIRED IMMUNEDEFICIENCY SYNDROME OR ACQUIRED IMMUNO-DEFICIENCY SYNDROME OR ACQUIRED IMMUNE-DEFICIENCY SYNDROME OR ACQUIRED IMMUN* DEFICIENCY SYNDROME in Title, Abstract, Keywords and (TASK-SHIFTING OR TASKSHIFTING OR (TASK* AND SHIFT*) OR TASK* OR (REFERR* AND (NURSE* OR PHARMAC*))) in Title, Abstract, Keywords and ANTIRETROVIRAL OR ANTI-RETROVIRAL OR ART OR cART OR HAART in Title, Abstract, Keywords
**WHO Global Health Library**	
	(TASK-SHIFTING OR TASKSHIFTING OR (TASK* AND SHIFT*) OR TASK* OR (REFERR* AND (NURSE* OR PHARMAC*)) AND (HIV* OR human immunodeficiency) AND (antiretroviral OR anti-retroviral))) OR (HIV AND task-shifting) OR (HIV* AND task* AND shift*)

For peer review only

Data collection and analysis

We based the methodology for data collection and analysis on the guidance of Cochrane Handbook of Systematic Reviews of Interventions [19].

Selection of studies for inclusion

Two review authors Nyanyiwe M. Mbeye (NMM) and Ola Adetokunboh (OA) read the titles, abstracts and descriptor terms of the downloaded citations to identify potentially eligible reports. We obtained full-text articles for all citations identified as potentially eligible by at least one of the two authors. The two authors independently inspected potentially eligible publications to establish the relevance of the article to the review according to the pre-specified criteria regarding study designs, participants, interventions, and comparisons and outcome measures. Differences between the two authors were discussed with a third author Charles Shey Wiysonge (CSW) or Tamara Kredo (TK) and resolved by consensus.

Data extraction and management

Two review authors (NMM and OA) independently extracted data onto standardised, pre-piloted data extraction forms. The following characteristics were extracted from each included study:

- **Study details:** Complete citations of publications associated with the study, start and end dates, location, study design characteristics, type of facility involved, investigators, funding sources, recruitment, method of randomisation, sequence

For peer review only

generation, method of allocation concealment, blinding of participants and personnel, blinding of outcome assessors, length of follow up, losses to follow up, withdrawals or drop-outs and other relevant details.

- **Details of the intervention:** details of the cadre of health worker who was dispensing or distributing ART, what training or other support or supervision they received and other relevant details.
- **Details of participants:** Trial inclusion and exclusion criteria, numbers of participants entering the trial, sex, clinical staging, CD4 count and other pertinent details.
- **Outcome details:** Definitions of outcomes, details of how outcomes were assessed, numerators and denominators associated with each outcome, completeness of outcome data, effect estimates reported and other relevant outcome information.

Assessment of risk of bias in included controlled trials

Two review authors (NMM and OA) independently assessed the risk of bias in each study using the Cochrane risk of bias tool which measures risk of bias in controlled trials across seven domains: sequence generation, allocation concealment, blinding of participants and personnel, blinding of outcome assessors, completeness of outcome data, selective outcome reporting and other potential biases [19].

Sequence generation (checking for selection bias)

We considered a study as having a low risk of selection bias due to sequence generation if investigators described a random component in the sequence-generation process, such as the use of random number tables; coin tossing, card or envelope shuffling. We classified a study as having a high risk of bias if investigators described

For peer review only - http://bmjopen.bmj.com/site/about/guidelines.xhtml

a non-random component in the sequence-generation process, such as the use of date of births, day of the week, or hospital or clinic record numbers. Where there was insufficient information to permit judgment about the sequence generation process, we classified the study as having unclear risk of bias.

Allocation concealment (checking for selection bias)

We classified the study as having a low risk due to allocation concealment if participants and investigators enrolling participants could not foresee assignment (e.g. central allocation; or sequentially numbered, opaque, sealed envelopes). A study was classified as having a high risk of selection bias if participants and investigators could foresee assignment (e.g. an open, random-allocation schedule; a list of non-random numbers; or use of unsealed, non-opaque, or not sequentially numbered envelopes. We assigned a study as having an unclear risk of selection bias when the study reported insufficient information to permit judgment of the allocation concealment or the methods used to conceal allocation were not described.

Blinding (checking for performance and detection bias)

We determined that a study was at low risk of performance bias when there was blinding of the participants and key study personnel, and it was unlikely that the blinding could have been broken or there was no blinding in the situation but non-blinding was unlikely to introduce bias. A study with no blinding or incomplete blinding when the outcome was likely to be influenced by lack of blinding was classified as having a high risk of performance bias. In the absence of sufficient information to permit judgment of adequacy of the blinding of participants and investigators, the study was classified as having unclear risk of performance bias.

Regarding detection bias, we determined that a study was at low risk of bias when there was blinding of outcome assessors. We classified a study as having a high risk

For peer review only

of detection bias if there was no blinding or the outcome was likely to be influenced by lack of blinding of outcome assessors. In the absence of sufficient information to permit judgment of adequacy of the blinding of outcome assessment, the study was classified as having unclear risk of detection bias.

Incomplete outcome data (checking for attrition bias)

We classified a study as having a low risk of attrition bias if there was no missing outcome data, reasons for missing outcome data were unlikely to be related to the outcome, or the proportion of missing outcome data was balanced across groups. We classified a study as having a high risk of attrition bias if there was imbalance in proportions lost to follow-up across groups and the reason for missing outcome data was likely to be related to the outcome. When there was insufficient reporting of attrition or exclusions, we classified the study as having an unclear risk of attrition bias.

Selective reporting (checking for reporting bias)

We determined a study as having a low risk of reporting bias if a protocol was available and primary outcomes in the final trial report corresponded closely to those presented in the protocol. High risk of selective reporting was considered to be present when the primary outcomes differed between the protocol and final trial report. When no trial protocol was available or there was insufficient reporting to determine if selective reporting was present, we classified the study as having an unclear risk of reporting bias.

Checking for other forms of bias

We classified a study as having a low risk of other forms of bias, when there was no evidence of bias from other sources. A study was classified as having a high risk from other sources when potential bias was present from other sources (e.g. early stopping

For peer review only

of trial, fraudulent activity, financial conflicts of interest). We assigned a study as having an unclear risk of bias if insufficient information was available to permit an adequate judgment of presence of other forms of bias.

Measures of effect

We calculated and presented summary statistics for the risk ratio (RR) for dichotomous outcomes and the weighted-mean difference for continuous outcomes, with their 95% confidence intervals (CI).

Unit of analysis issues

The unit of analysis was the individual study participant for all the trials.

Dealing with missing data

We contacted study authors for one of the included trials to obtain information on the composition of health professionals in the control groups involved in dispensing ART in order to establish whether pharmacy personnel were part of the team. Unfortunately the author could not remember details but assumed that health professionals also included pharmacy personnel.

Assessment of heterogeneity

We used the I^2 statistic to measure heterogeneity among the trials. We planned to explore substantial heterogeneity (I^2 >50%) by pre-specified subgroup analysis. However, there was no evidence of serious heterogeneity in our review hence we did not perform sub-group analyses.

Assessment of reporting biases

We contacted study authors to provide any missing outcome data. However, only one author from one included trial responded but did not provide any additional

For peer review only

information. If any meta-analysis in our review was to include 10 or more studies, we planned to assess the potential for publication bias using a funnel plot [19 20]. We minimised the potential for publication bias by using a comprehensive search strategy, as described above.

Data synthesis

We conducted meta-analysis, using Cochrane's Review Manager software [21]. We used a random effects model considering the diverse settings of the included studies. Where meta-analysis was not possible, for instance, for adherence and cost, a narrative synthesis of the evidence was carried out. We summarised the quality (or certainty) of evidence for each outcome using GRADE [22 23]. For GRADE we independently considered the five factors for downgrading the evidence to assess the certainty of a body of evidence as it relates to the studies that contribute data to the meta-analyses for the pre-specified outcomes. The quality rating across studies has four levels: high, moderate, low or very low. Randomised trials are considered to be of high quality but can be downgraded for any of the following five reasons: risk of bias; indirectness of evidence; unexplained heterogeneity or inconsistency of results; imprecision of results; and, high probability of publication bias. Similarly, observational studies are considered to be of low quality, but can be upgraded for any of these three reasons: large magnitude of effect; all plausible confounding would reduce a demonstrated effect; and, the presence of a dose-response gradient.

Subgroup analysis and sensitivity analysis

Due to the absence of significant heterogeneity, we did not perform subgroup analyses. Additionally, sensitivity analyses were not conducted to investigate the

For peer review only

effect of excluding studies with high or low risk of bias due to the small number of studies included in the review.

Results

A total of 3557 records were identified. Following title and abstract screening, eight studies were identified for full-text screening. The full-text review resulted in three studies [15 24 25] that met the review inclusion criteria. The study selection process is shown in Figure 1. The characteristics of included and excluded studies are shown in Tables 3 and 4 respectively. In total, 1993 HIV-infected patients on ART were enrolled in the three included studies. Of these, 1121 were assigned to non-pharmacy personnel model of ART delivery while 872 were assigned to pharmacy personnel model of ART delivery. However, 974 and 746 were available for analysis in the non-pharmacy personnel and pharmacy personnel groups respectively for all the three studies. Overall, there were no differences in loss to follow up between non-pharmacy and pharmacy personnel groups (3% versus 3%) p=0.9. Two studies [15], [24] included lay people in the non-pharmacy personnel group as shown in Table 3 while the third [25] included nurses in the non-pharmacy personnel group.

For peer review only

Table 3: Summary of included studies

Study ID	Setting	Methods	Outcomes
Selke 2010 [15]	Kenya	**Study design:** Cluster randomised trial<br><br>**Description of interventions:**<br>**Non-pharmacy personnel (home-based) group:**<br>Community Care Counsellors were:<br>• Patients were clinically stable with self-reported 100% adherence to ART over the six-month period before recruitment<br>• Considered by the clinic staff to be a good role model and mentor for other patients<br>Duties for care givers during home visits:<br>• Obtained and entered data concerning patients' symptoms<br>• Vital sign assessment<br>• Assessment of adherence to ART and opportunistic infections prophylaxis<br>• Distributed a one-month supply of the patients' medications (from a prefilled kit)<br><br>**Pharmacy personnel (facility-based) group:**<br>• Nurses/clinical officers or physicians performed the following:<br>○ took an interim medical history<br>○ addressed any acute concerns<br>○ reviewed medications<br>○ prescribed ART and opportunistic infection prophylaxis<br>• Patients collected a one-month supply of their medication dispensed from the pharmacy	• Adherence<br>• Viral load responses<br>• Intercurrent opportunistic infections<br>• Hospitalisation<br>• Loss to follow up<br>• Change in second-line therapy<br>• Mortality
Jaffar 2009 [24]	Uganda	**Study design:** Cluster-randomised trial	• Plasma RNA VL>500 copies per

For peer review only

For peer review only - http://bmjopen.bmj.com/site/about/guidelines.xhtml

		**Description of interventions:**<br>**Non-pharmacy personnel (home-based care) group**<br>• Field workers with degree or college diploma received a week of intensive training at start of study and yearly refresher courses on:<br>○ The principles of antiretroviral therapy<br>○ Adherence support<br>• Field workers visited patients in the homes on motor bikes every month to:<br>○ Deliver drugs<br>○ Monitor participants with a checklist of signs and symptoms of drug toxicity and disease progression<br>○ Provide adherence support<br>○ Referred patients to physician or counsellor at the clinic when judged necessary<br>• At two and six months after starting therapy, all patients were reviewed by a Medical Officer at the clinic and then at twelfth month<br>• Drugs were not dispensed during the clinic visits for this group<br>• Patients were visited again when not found at home<br>• Patients asked to come to clinic when unwell<br><br>**Pharmacy personnel (facility-based) group:**<br>• Patients collected drugs every month from the pharmacy<br>• Routine reviews by a Medical Officer and counsellor at two and three months after start of treatment & every three months thereafter<br>• Patients assessed by a nurse and referred to a doctor when necessary during clinic visits<br>• Patients followed up at home by field workers when missed an appointment<br>• Patients received vouchers for their households for free voluntary counselling and testing at the clinic	mL<br>• Either plasma RNA >500 copies per mL if undetectable at six months, or an increase of 1000 copies between two consecutive tests if RNA detectable at six months<br>• All-cause mortality<br>• Mortality or plasma RNA VL >500 copies per mL<br>• Admitted on one or more occasions<br>• All admissions<br>• Death, first admission, or change to second-line therapy<br>• Frequency of outpatient attendance<br>• Adherence<br>• Costs of health service delivery and costs incurred by patients to access care

For peer review only

For peer review only - http://bmjopen.bmj.com/site/about/guidelines.xhtml

		• Patients asked to come to clinic when unwell	
Silveira 2014 [25]	Brazil	**Study design:** Individually randomised trial<br><br>**Description of interventions:**<br>**Pharmacy personnel group**<br>Patients received this intervention on a monthly basis through the Da´der method in which consisted of a series of scheduled meetings at which the pharmacist and patient addressed, reviewed, and solved drug-related problems for a 12-month period. Patients received structured counselling from pharmacists on their prescription regimens at the time of their initial drug dispensing and at monthly refill visits.<br>Key elements included:<br>• Reviewing the prescription with the patient<br>• Reviewing a card on which medications were colour-coded to facilitate recognition and reduce confusion that might arise from complicated drug names<br>• Reviewing the schedule, length and date of next appointment<br>• Reviewing patient's understanding of the prescription by asking patient to describe it and giving patients verbal information on the expected side effects of their medications and instructing them to seek medical assistance by calling the pharmacist if side effects occurred.<br><br>**Non-pharmacy personnel group**<br>Patients received Usual Care (UC) for ART drug distribution. The control group received UC for ART drug delivery. This included the following:<br>At the first appointment, when patients received their medications, a nurse first provided information on:<br>• the regimen,<br>• how and when to use the medications,	• Self-reported adherence calculated by taking the number of tablets that patients reported ingesting and diving it by the number they should have ingested. Patients were classified as adherent if they reported using 95% or more of the tablets prescribed<br>• Depression: This was investigated using a validated Portuguese-language version of BDI with standard scoring

For peer review only

		• principal side effects<br>• importance of adhering to the prescription.<br><br>Afterward, patients picked up their medications at a drug-dispensation counter. They had no encounters with a pharmacist. Patients were informed that they could schedule time with a nurse for any questions about their treatment or disease.<br>In both groups, patients were scheduled every four months for medical appointments and measurements of viral load and CD4 count. Participants who did not appear for their regularly scheduled appointment were contacted by telephone and asked to return.	

For peer review only

Table 4: Summary of excluded studies

Study ID	Reasons for exclusion
March 2007 [26]	All participants were enrolled in the pharmacist-managed HIV drug optimisation clinic, with no non-pharmacist control group
Chang 2010 [27]	Non-pharmacy personnel did not dispense or distribute ART
Kiweewa 2013 [28]	Dispensing of ART done in same way for both groups
Hansudewechaku 2012 [29]	Cohort study
Henderson 2011 [30]	Observational study with no comparison group.

Risk of bias in included studies

We judged the three trials to have a low risk of selection bias as a result of adequate randomisation and allocation concealment. The studies by Selke et al [15] and Jaffar et al [24] were judged to have low risk of performance bias for blinding because the reported outcomes were objective and unlikely to be influenced by lack of blinding. However, the Selke trial [15] did not give sufficient information to permit judgment of whether or not detection bias was present. The Silveira trial [25] was judged to have a high risk of performance and detection bias due to lack of blinding for participants, personnel and outcome assessment respectively for all study outcomes. The three studies were judged to have a low risk of attrition bias as they did not have differential or large numbers of losses to follow-up across the intervention arms. The risk of selective reporting bias was judged to be low for the study by Jaffar et al [24] and unclear for the Selke trial [15] because the trial protocol was not available. The Silveira trial [25] was also judged as having unclear risk of reporting bias due to insufficient information. It [25] was also judged as having a high risk of other biases due to

For peer review only

inadequate sample size. Figure 2 provides a graphical summary of the risk of bias assessments.

Mortality

There was one reported death in the non-pharmacy personnel group in the Selke trial [15] during the study period. However, the authors reported the death as unrelated to HIV but rather due to use of herbal abortifacients. The trial by Jaffar [24] in Uganda did not find differences in mortality rates between the non-pharmacy and pharmacy personnel groups: adjusted rate ratio RR 0.95 (95% CI 0.71 to 1.28). Five deaths were reported in the non-pharmacy personnel group compared to none in the pharmacy personnel group in the Silveira trial [25]. This difference was not statistically significant (RR 11.00, 95% CI 0.61 to 197.35). When these data were pooled as shown in Figure 3, there was no difference in mortality between the groups cared for by non-pharmacy personnel and those cared for by pharmacy personnel (RR 1.86; 95% CI 0.44 to 7.95), with no significant heterogeneity (Chi^2 = 3.34; df = 2; p = 0.19; I^2 = 40%). The certainty of the evidence for this outcome was low due to serious indirectness and imprecision as shown in Table 5. We downgraded for indirectness because two of the three trials (Selke [15] and Jaffar [24]) compared complex interventions that included, but were not limited to, pharmacy and non-pharmacy personnel as shown in Table 5. There were few deaths in the trials and the effect estimate was imprecise, with wide confidence intervals ranging from appreciable benefit to substantial harm as reported in Table 5.

For peer review only - http://bmjopen.bmj.com/site/about/guidelines.xhtml

Table 5: GRADE Summary of findings table for pharmacy versus non-pharmacy personnel for dispensing antiretroviral therapy

Population: People living with HIV
Setting: Brazil, Kenya, and Uganda (one study per country)
Intervention: Non-pharmacy personnel for dispensing antiretroviral therapy
Control: Pharmacy personnel for dispensing antiretroviral therapy

**Outcomes**<br><br>(Mean follow-up: 12 months)	**Pharmacy personnel**	**Non-pharmacy personnel**	**Relative effect (95% CI)**	**No of Participants (studies)**	**Quality of evidence (GRADE)**
**Mortality**	**76 per 1000**	**141 per 1000**<br>(33 to 602)	**RR 1.86**<br>(0.44 to 7.95)	1993<br>(three studies)	⊕⊕⊖⊖<br>**low**[1,2]
**Virological failure**	**150 per 1000**	**138 per 1000**<br>(109 to 172)	**RR 0.92**<br>(0.73 to 1.15)	1752<br>(three studies)	⊕⊕⊖⊖<br>**low**[1,2]
**Loss to follow up**	**28 per 1000**	**31 per 1000**<br>(19 to 53)	**RR 1.13**<br>(0.68 to 1.91)	1993<br>(three studies)	⊕⊕⊖⊖<br>**low**[1,2]

CI: Confidence interval; **RR:** Risk ratio;

GRADE Working Group grades of evidence
High certainty: Further research is very unlikely to change our confidence in the estimate of effect.
Moderate certainty: Further research is likely to have an important impact on our confidence in the estimate of effect and may change the estimate.
Low certainty: Further research is very likely to have an important impact on our confidence in the estimate of effect and is likely to change the estimate.
Very low certainty: We are very uncertain about the estimate.

[1] We downgraded by one for serious indirectness: Two trials compared complex interventions that included, but were not merely limited to, pharmacy and non-pharmacy personnel.
[2] We downgraded by one for serious imprecision: There are few events and the effect estimates have wide confidence intervals, ranging from appreciable benefit to harm.

For peer review only

For peer review only - http://bmjopen.bmj.com/site/about/guidelines.xhtml

Virological response

In the Selke trial [15], we did not find significant differences between groups in the proportions of undetectable viral load (10.5% in the intervention group versus 13.5% in the control group), p = 0.65 at 12 months.

Jaffar et al [24] investigated HIV-RNA suppression and survival over time by study group. They found that rates of detection of plasma viral loads of more than 500 copies per mL after six-month visit were similar in both groups (RR 1.04, 95% CI: 0.78 to 1.40). Similarly, the composite rates of either plasma RNA viral loads of more than 500 copies per mL after a six-month visit were comparable in those who had undetectable viral loads at six months, or an increase in plasma RNA viral load of 1000 copies per mL between two consecutive tests in those who had detectable viral loads at six months (RR: 0.99, 95% CI: 0.73 to 1.34). By the end of the study 66% of the participants in the non-pharmacy personnel group and 63% of the participants in the pharmacy personnel group had undetectable viral loads.

Silveira [25] in Brazil did not report significant differences in viral load at baseline. In the pharmacy personnel group 52.1% (n=86) and in the non-pharmacy personnel group 51.2% (n=85), p=0.86 were reported to have undetectable viral load. However, fifty participants (23 in the pharmacy personnel group and 27 in the non-pharmacy personnel group) were reported to have detectable viral load at baseline. For these, the authors conducted a sub-group analysis after two years of follow up. Seventy-six per cent (95% CI, 50.0 to 94.7) in the pharmacy personnel group and 42.9% (95% CI, 23.9 to 68.3) in the non-pharmacy personnel group cumulatively had virological success.

For peer review only - http://bmjopen.bmj.com/site/about/guidelines.xhtml

In a meta-analysis, we detected no differences in virological failure between the group cared for by non-pharmacy personnel and that cared for by pharmacy personnel (RR 0.92; 95% CI: 0.73 to 1.15), with no significant heterogeneity detected between the trials ($Chi^2 = 0.24$; df = 2; p=0.89; $I^2 = 0\%$) as shown in Figure 4. The quality of the evidence for this outcome was low, due to serious indirectness and imprecision as shown in Table 5.

Loss to follow up

Selke [15] reported no differences in median duration (days) in the study for both groups at six or 12 months (343 days for both groups at 12 months), p = 0.2. At study closure, the proportion lost to follow up was similar between the two groups (5.2% for the non-pharmacy personnel group and 4.5% for the pharmacy personnel group), p = 1.0. Jaffar [24] reported very low rates of loss to follow up (1% in the non-pharmacy versus 2% in the pharmacy personnel group), however, 3% in the non-pharmacy personnel group and 6% in the pharmacy personnel group respectively withdrew from participation. Similarly, in the Silveira trial [25], 4.8% (n = 8) participants were lost to follow up in the non-pharmacy personnel group and 3% (n = 5) in the pharmacy personnel group. Overall, there were no differences in loss to follow up between the non-pharmacy personnel and the pharmacy personnel group with both having a 3% loss to follow up, p=0.96.

In our meta-analysis, we detected no difference in loss to follow up between the groups (RR 1.13; 95% CI: 0.68 to 1.91), with no significant heterogeneity ($Chi^2 = 0.54$; df = 2; p=0.76; $I^2 = 0\%$) as shown in Figure 4. The quality of the evidence for this outcome was low due to serious indirectness and imprecision as shown in Table 5.

For peer review only - http://bmjopen.bmj.com/site/about/guidelines.xhtml

Clinic visits and incidence of opportunistic infections

Selke [15] reported significantly more clinic visits in the pharmacy compared to the non-pharmacy personnel group (mean visits 12.6 versus 6.4), p < 0.001. Despite fewer clinic visits for the non-pharmacy personnel group, clinical outcomes between the two groups were comparable with an incidence of 13.6 HIV opportunistic infections per 100 person-years in the non-pharmacy personnel group and 19.8 HIV opportunistic infections per 100 person-years in the pharmacy personnel group, p = 0.42. Although the non-pharmacy personnel group was found to have fewer clinic visits, the authors observed that this group undertook 64% more clinic visits than originally scheduled. The study by Jaffar [24] found a high frequency of outpatient attendance (15 242 visits) in the pharmacy compared to the non-pharmacy personnel group (6691 visits). However, they found similar distribution of new diagnoses between groups where more than 50% were infectious and parasitic infections. The trial by Silveira [25] did not report on clinic visits.

Adherence

Both groups in the Selke trial [15] demonstrated high levels of self-reported medication adherence with no statistical differences between groups at six and 12 months (96% versus 97%), p = 0.71 and (94% versus 97%), p = 0.47 respectively. In the Jaffar trial [24], self-reported adherence at routine clinical and counselling reviews was high with 94% in the intervention group and 91% in the control group. In the Silveira trial [25], participants' self-reported adherence remained the same between the two groups after controlling for potential confounders RR 1.05, 95% CI 0.95-1.15, p=0.35.

Cost

In the trial by Jaffar [24], a societal perspective economical analysis showed a higher mean cost per patient per year in the pharmacy personnel group ($838) for health

For peer review only - http://bmjopen.bmj.com/site/about/guidelines.xhtml

services (staff, transport, drugs, laboratory and clinical services, sensitisation, training and workshops, utilities, supervision and overhead capital) compared to non-pharmacy personnel group ($793). Similarly, total per patient costs to access care per year were higher in the pharmacy personnel group ($54) compared to non-pharmacy personnel group ($18). Costs to access care included transport, lunch, childcare and lost work time. The outcome cost was not pre-specified in the protocol, however, this provides additional data that may be relevant for decision makers and was therefore included for consideration.

Acceptability to pharmacy personnel, non-pharmacy personnel and patients and harm, including error rates

These outcomes were not reported in the included trials.

Discussion

Summary of main results

Two cluster randomised clinical trials and one non-blinded randomised controlled trial were included in this review of effects of shifting responsibility from pharmacy to non-pharmacy personnel for adherence assessment and dispensing antiretroviral drugs to HIV-infected patients.

In our meta-analysis, we found low quality evidence that there may probably be no difference in mortality, virological response, and loss to follow up between non-pharmacy personnel and pharmacy personnel distributing ART to patients. In addition, we did not observe significant differences in adherence to treatment. Selke et al [15] reports that the non-pharmacy personnel group had significantly fewer all-cause

For peer review only - http://bmjopen.bmj.com/site/about/guidelines.xhtml

sick visits to the clinic compared to the pharmacy personnel group. Although the main difference between the Selke et al [15] and Jaffar et al [24] was the level of education of the non-pharmacy personnel, it did not seem to affect their overall performance [15 24]. Additionally, the Jaffar trial [24] found the non-pharmacy dispensing strategy to be cost–effective and cheaper to run than the pharmacy dispensing strategy by almost 45 US dollars per patient per annum and this accounted for only 6% of the total cost of healthcare service expenditure for the intervention group [24]. The patients who were accessing their medications and other healthcare services from pharmacy personnel incurred more costs in terms of transportation, lunch, childcare costs and lost work time. In the first year of the study, the pharmacy personnel group incurred double the cost of healthcare services per patient per annum compared to the non-pharmacy personnel group. This is a challenge in most poor settings in Africa where many cannot afford basic necessities and this further impedes their access to treatment [24]. Lack of or inadequate financial resources are major factors in late presentation to health facilities, poor access to HIV care and support, and low retention in care after initiation of ART [31 32]. Although health services are provided for free in most public institutions, the cost of accessing the services may serve as a hindrance to achieving optimal care and support for HIV-infected patients [31]. The non-pharmacy personnel dispensing ART could therefore considerably reduce the cost of services for both patients and governments with potentially favourable clinical outcomes.

Although issues related to drug-drug interaction, medication errors, pre-screening for ART and some special cases of adherence to therapy may be beyond the capacity of non-pharmacy personnel, targeted pharmacy support to non-pharmacy personnel could offer benefits to patients having challenges with their medications especially those who are taking concomitant medications with ART [33].

For peer review only

For peer review only - http://bmjopen.bmj.com/site/about/guidelines.xhtml

This review and meta-analysis has shown that the use of non-pharmacy personnel comprising either lay people or other health professionals such as nurses who are given well-tailored and comprehensive short trainings with refreshers could effectively support ART dispensing systems for HIV-infected patients. The use of non-pharmacy personnel such as lay people who are known and trusted members of the community in dispensing ART and monitoring patients' health would enable identification of vital psychosocial features that might be overlooked by physicians, nurses and pharmacists at the facility. Some of these psychosocial problems such as gender-based violence, food insecurity and alcohol abuse negatively impact on adherence and retention in care [15]. In the case of community-based non-pharmacy personnel, they could also serve as a link between the patients and other healthcare workers and facilitate smooth communication between the patients and the pharmacists on issues such as changes in regimen and dosage of drugs [15 33].

Overall completeness and applicability of evidence

Although it may not be possible to rule out publication and language biases in systematic reviews and that this could potentially threaten the conduct of this review, our trial search was not restricted to any language, setting or publication status. We are of the opinion that we identified all the existing randomised controlled trials relevant to our research question. However, there is a possibility that some unpublished or published trials were not accessible by our search.

We identified three studies for inclusion in this review. The trials generally had small sample sizes. Recruitment restrictions such as basing recruitment on location of residence of the participants resulted in a small sample size in the Selke trial [15]. The

For peer review only - http://bmjopen.bmj.com/site/about/guidelines.xhtml

trials did not include some of the outcomes of interest for decision-making such as acceptability to participants and feasibility of the intervention, for example, adherence to treatment.

Certainty of evidence

We assessed the certainty or quality of evidence using the Grading of Recommendations Assessment, Development and Evaluation (GRADE) approach [34], which defines the certainty of evidence for each outcome as "the extent of our confidence that the estimates of effect are correct" [35]. Evidence from this review should be applied with caution considering its low quality. Three outcomes in this review were found to be of low quality. We are therefore not confident enough to state that the estimates lie close to the true value and it is possible that different and further studies are needed to contribute to the body of knowledge to increase our certainty in these review findings.

Conclusions

There is low-quality evidence that the use of non-pharmacy personnel including lay people in dispensing and distribution of ART may be as effective as pharmacy personnel performing these functions in resource-limited settings when accompanied by continued capacity building and supervision by trained pharmacy personnel. The introduction of non-pharmacy personnel is probably cost-effective for the patient and health system. Strong referral systems are crucial in task-shifting of dispensing responsibilities to non-pharmacy personnel to support patients that require advanced medical attention. Due to the critical shortage of human personnel in the health sector, most HIV programmes engaged other cadres of health personnel other than doctors to prescribe and dispense ART at the onset of their programme implementation. This

For peer review only - http://bmjopen.bmj.com/site/about/guidelines.xhtml

might have contributed to scarcity of studies on the use of pharmacy personnel for dispensing and distributing ART. More trials, with adequate sample sizes to detect clinically relevant health benefits are therefore needed to investigate task-shifting of dispensing and distributing ART from pharmacy to non-pharmacy personnel.

Author contributions

EN, TK, and CSW conceived the idea; NMM, TK and CSW drafted the review protocol; NMM, OA, TK and CSW conducted screening, selected the studies, extracted data, performed analyses and drafted the manuscript. All authors reviewed and approved the manuscript.

Acknowledgements

Funding for preparing this review was provided by the Department of HIV/AIDS at the WHO Headquarters in Geneva, Switzerland through a grant from University of California, San Francisco. The authors would like to thank George Rutherford and Hacsi Horvath (Global Health Sciences, University of California, San Francisco, USA) for support and specific assistance in developing the search strategy for electronic databases. Neither the authors' institutions nor the funders played a role in preparing the manuscript, and the views expressed therein are solely those of the authors. We would like to thank Joy Oliver from Cochrane South Africa, South African Medical Research Council for assistance with the updated search. Both Charles Wiysonge and Tamara Kredo are partly supported by the Effective Health Care Research Consortium. This Consortium is funded by the UK government for the

For peer review only - http://bmjopen.bmj.com/site/about/guidelines.xhtml

benefit of developing countries (Grant: 5242). The views expressed in this publication do not necessarily reflect UK government policy.

Competing interests

None declared

References

1. UNAIDS. How AIDS changed everything - MDG 6: 15 years, 15 lessions of hope from the AIDS response. http://wwwunaidsorg/en/resources/presscentre/pressreleaseandstatementarchive/2015/july/20150714 PR MDG6report 2015;**accessed 22nd September 2015**.
2. Palella FJ DK, Moorman AC, Loveless MO, et al. . Declining morbidity and mortality among patients with advanced human immunodeficiency virus infection. New England Journal of Medicine 1998;**338**(13):853-60.
3. Anglemyer A RG, Horvath T, Baggaley RC, Egger M, Siegfried N. . Antiretroviral therapy for prevention of HIV transmission in HIV-discordant couples. Cochrane Database Syst Rev 2013;**4**(CD009153).
4. Ford N, Calmy, A., Mills, E,J. The first decade of antiretroviral therapy in Africa. Globalization and Health 2011;**7**(33).
5. King RC, Fomundam, H.N. Remodeling pharmaceutical care in sub-Saharan Africa (SSA) amidst human resources challenges and the HIV/AIDS pandemic. Int J Health Plann Manage 2010;**25**(1):30-48.
6. SAPC. Pharmacy Human Resources data. http://wwwhealthlingorgza/healthstats/283/data;accessed **15th August, 2015**.
7. Tseng A, Foisy, M., Hughes, C.A., et, al.,. Role of pharmacists in caring for patients with HIV/AIDS: Clinical Practice Guidelines. CJHP 2012;**65**(2):125-45.
8. Saberi P, Dong, B.J., Johnson, M.O., Greenblatt, R.M., Cocohoba, J.M. The impact of HIV clinical pharmacists on HIV treatment outcomes: systematic review. Patient Prefer Adherence 2012;**6**:297-322.
9. Kitahata MM, Koepsell, T.D., Deyo, R.A., et, al. Physicians experience with the acquired immunodeficiency syndrome as a factor in patients' survival. N Engl J Med 1996;**334**(11):701-6.
10. Landon BE, Wilson, I..B., Malness, K., et, al. Physician specialization and the quality of care for human immunodeficiency virus infection. Arch Intern Med 2005;**165**(10):1133-9.

For peer review only - http://bmjopen.bmj.com/site/about/guidelines.xhtml

For peer review only

11. Kredo T AF, Bateganya M, Pienaar ED. . Task shifting from doctors to non-doctors for initiation and maintenance of antiretroviral therapy. Cochrane Database of Systematic Reviews 2014;**CD007331**(7).

12. MSH. *Managing Drug Supply*. Second Edition ed. 14 Oakwood Avenue, West Hartford, Connecticut, 06119-227 USA: Kumarian Press Inc, 1997.

13. Decroo T, Telfer, B., Biot M, et al,. . Distribution of antiretroviral treatment through self-forming groups of patients in Tete province, Mozambique. J Acquir Immune Defic Syndr 2011;**56**:39-44.

14. Kipp W, KondeLule, J., Rubaale,T., et al. . Comparing antiretroviral treatment outcomes between a prospective community-based and hospital-based cohort of HIV patients in rural Uganda. BMC Int Health Hum Rights 2011;**11Suppl**(2):S12.

15. Selke H, Kimaiyo, S., Sidle, J.E., et, al., . Task-shiftingofantiretroviraldelivery from health care workers to persons living with HIV/AIDS: clinical outcomes of a community-based program in Kenya. J Acquir Immune Defic Syndr 2010;**55: 483-90.**

16. Callaghan M, Ford, N., Schnider, H. Asystematic review of task-shifting for HIV treatment and care in Africa. Human Resources for health 2010;**8**(8).

17. Laurant M RD, Hermens R, Braspenning J, Grol R, Sibbald B. . Substitution of doctors by nurses in primary care. Cochrane Database of Systematic Reviews 2005;**CD001271**.

18. Mbeye NM, Kredo, T., Wiysonge, C.S. The effects of shifting tasks from pharmacy to non-pharmacy personnel for providing antiretroviral therapy to people living with HIV: a systematic review protocol. BMJ Open 2016;**6:e008195**.

19. Higgins JPT, Green, S. (eds). . Cochrane Handbook for Systematic Reviews of Interventions. Wiley-Blackwell 2008.

20. Egger M DSG, Schneider M, Minder C. . Bias in meta-analysis detected by a simple, graphical test. BMJ 1997;**315**(7109):629-34.

21. (RevMan) RM. Computer program. The Nordic Cochrane Centre, The Cochrane Collaboration, Copenhagen 2015;**Version 5.3**.

22. Guyatt GH OA, Santesso N, Helfand M, Vist G, Kunz R, et al. . GRADE guidelines: 12. Preparing Summary of Findings tables-binary outcomes. Journal of Clinical Epidemiology 2013;**66**(2):158-72.

23. Guyatt GH TK, Oxman AD, Walter SD, Patrick D, Furukawa TA, et al. . GRADE guidelines: 13. Preparing Summary of Findings tables and evidence profiles-continuous outcomes. Journal of Clinical Epidemiology 2013;**66**(2):173-83.

24. Jaffar S, Amuron B., Foster S., et, al., . Rates of virologic failure in patients treated in a home-based versus a facility-based HIV-care model in Jinja, southeast Uganda: a cluster-randomised equivalence trial. Lancet 2009;**DOI:10.1016/S1040-6736(09)61674-3.**

25. Silveira MPT, Guttier, M.C., Page, K., Moreira, L.B. Randomized Controlled Trial to Evaluate the Impact
of Pharmaceutical Care on Therapeutic Success in HIV-Infected
Patients in Southern Brazil. AIDS Behav 2014;**18**:S75-S84.

26. March K MM, Louie SG. . Effects of pharmacists' interventions on patient outcomes in an HIV primary care clinic. American Journal of Health-System Pharmacy 2007;**64**:2574-78.

For peer review only - http://bmjopen.bmj.com/site/about/guidelines.xhtml

27. Chang LW KJ, Nakigozi G, Ssempijja V, Packer AH, Serwadda D, Quinn TC, Gray RH, Bollinger RC, Reynolds SJ. . Effect of peer health workers on AIDS care in Rakai, Uganda: A cluster-randomized trial. PLoS ONE 2010:; **5** (6).

28. Kiweewa FM WD, Nakibuuka J, Mubiru M, Bagenda D, Musoke P, Fowler MG, Antelman G. . Noninferiority of a task-shifting hiv care and treatment model using peer counselors and nurses among ugandan women initiated on ART: Evidence from a randomized trial. Journal of Acquired Immune Deficiency Syndromes 2013;**63** (4):e125-e32.

29. Hansudewechakul R NT, Katana A, Faikratok W, Lolekha R, Thainuea V, McConnell MS. . Successful clinical outcomes following decentralization of tertiary paediatric HIV care to a community-based paediatric antiretroviral treatment network, Chiangrai, Thailand. Journal of the International AIDS Society 2012;**15 (2)**

30. Henderson KC HJ, Johnson SC, Valuck RJ, Kiser JJ. . Assessing the effectiveness of pharmacy-based adherence interventions on antiretroviral adherence in persons with HIV. IDS Patient Care and STDs **25**(4):221-28.

31. Hardon A, Akurut, D., Comoro, C., et al.,. Hunger, waiting time and transport costs: time to confront challenges to ART adherence in Africa. AIDS Care 2007;**19: 658–65.**

32. Rosen S, Fox, MP., Gill, C.J. . Patient retention in antiretroviral therapy programs in sub-Saharan Africa: a systematic review. PLoS Med 2007;**4: e298.**

33. Seden K, . Bradley, M., Miller, AR., Beadsworth, M.B., Khoo, S.H. . The clinical utility of HIV outpatient pharmacist prescreening to reduce medication error and assess adherence. Int J STD AIDS 2013:;**24 (3);237-41.**

34. Guyatt GOA, Akl, E.A., Kunz, R., Vist, G., Brozek, J., Norris, S,. Falck-Ytter, Y., Glasziou, P., DeBeer, H., Jaeschke, R,. Rind, D., Meerpohl, J., Dahm, P., Schünemann, H.J. . GRADE guidelines: 1. Introduction-GRADE evidence profiles and summary of findings tables. Journal of Clinical Epidemiology 2011;**64(4): 383-94**.

35. Higgins JP, editor. Chichester, England: . Cochrane handbook for systematic reviews of interventions. England. Wiley-Blackwell 2008.

List of figures

Fig 1: Flow diagram showing the search and selection of studies

Fig 2: Summary of risk of bias in included studies

Fig 3: Effect of shifting dispensing of ART from pharmacy to non-pharmacy personnel on mortality

Fig 4: Effect of shifting dispensing of ART from pharmacy to non-pharmacy personnel on virological failure

For peer review only

For peer review only - http://bmjopen.bmj.com/site/about/guidelines.xhtml

Fig 5: Effect of shifting dispensing of ART from pharmacy to non-pharmacy personnel on loss to follow up

Supporting Information

S1. Task-shifting review protocol

For peer review only

For peer review only - http://bmjopen.bmj.com/site/about/guidelines.xhtml

Fig 1

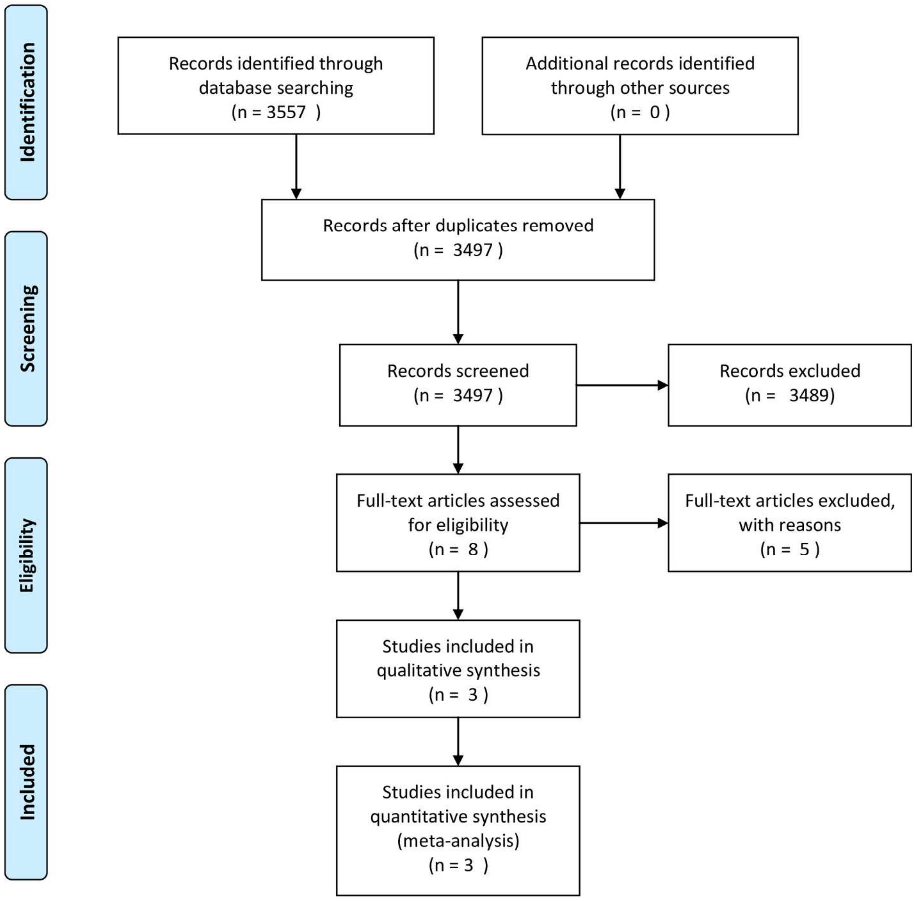

Fig 1: Flow diagram showing the search and selection of studies

168x222mm (300 x 300 DPI)

Fig 2.

Domain	Trial		
	**Jaffar 2009**	**Selke 2010**	**Silveira 2013**
Random sequence generation (selection bias)	+	+	+
Allocation concealment (selection bias)	+	+	?
Blinding of participants and personnel (performance bias)	+	+	−
Blinding of outcome assessment (detection bias)	+	?	−
Incomplete outcome data (attrition bias)	+	+	+
Selective reporting (reporting bias)	+	?	?
Other bias	+	?	−

+ Low risk; − High risk; ? Unclear risk.

Fig 2: Summary of risk of bias in included studies

144x94mm (300 x 300 DPI)

Fig 3

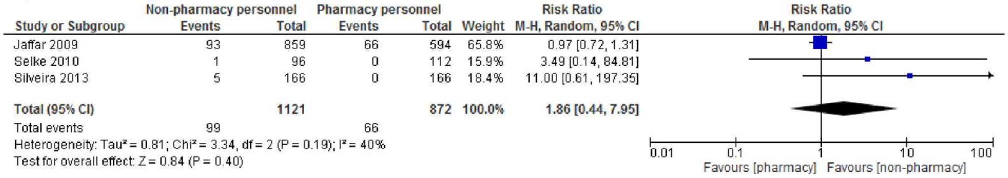

Fig 3: Effect of shifting dispensing of ART from pharmacy to non-pharmacy personnel on mortality

184x35mm (300 x 300 DPI)

For peer review only

Fig 4

Study or Subgroup	Non-pharmacy personnel Events	Non-pharmacy personnel Total	Pharmacy personnel Events	Pharmacy personnel Total	Weight	Risk Ratio M-H, Random, 95% CI
Jaffar 2009	106	729	74	483	71.4%	0.95 [0.72, 1.25]
Selke 2010	9	96	13	112	8.3%	0.81 [0.36, 1.81]
Silveira 2013	23	166	27	166	20.3%	0.85 [0.51, 1.42]
**Total (95% CI)**		**991**		**761**	**100.0%**	**0.92 [0.73, 1.15]**
Total events	138		114			

Heterogeneity: $Tau^2 = 0.00$; $Chi^2 = 0.24$, df = 2 (P = 0.89); $I^2 = 0\%$

Test for overall effect: Z = 0.74 (P = 0.46)

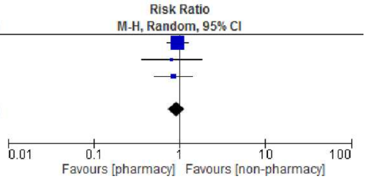

Fig 4: Effect of shifting dispensing of ART from pharmacy to non-pharmacy personnel on virological failure

187x36mm (300 x 300 DPI)

For peer review only

Fig 5

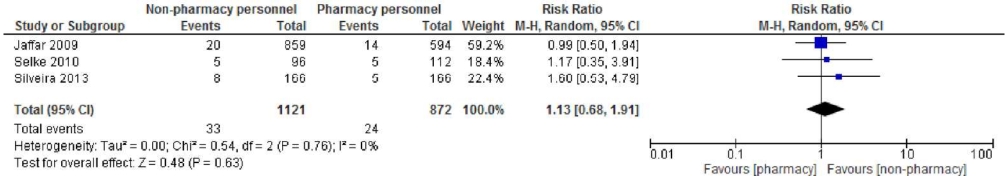

Fig 5: Effect of shifting dispensing of ART from pharmacy to non-pharmacy personnel on loss to follow up

189x37mm (300 x 300 DPI)

For peer review only

S2. PRISMA 2009 Checklist

Section/topic	#	Checklist item	Reported on page #
**TITLE**			
Title	1	Identify the report as a systematic review, meta-analysis, or both.	1
**ABSTRACT**			
Structured summary	2	Provide a structured summary including, as applicable: background; objectives; data sources; study eligibility criteria, participants, and interventions; study appraisal and synthesis methods; results; limitations; conclusions and implications of key findings; systematic review registration number.	2-3
**INTRODUCTION**			
Rationale	3	Describe the rationale for the review in the context of what is already known.	7
Objectives	4	Provide an explicit statement of questions being addressed with reference to participants, interventions, comparisons, outcomes, and study design (PICOS).	8
**METHODS**			
Protocol and registration	5	Indicate if a review protocol exists, if and where it can be accessed (e.g., Web address), and, if available, provide registration information including registration number.	8
Eligibility criteria	6	Specify study characteristics (e.g., PICOS, length of follow-up) and report characteristics (e.g., years considered, language, publication status) used as criteria for eligibility, giving rationale.	8-9
Information sources	7	Describe all information sources (e.g., databases with dates of coverage, contact with study authors to identify additional studies) in the search and date last searched.	9
Search	8	Present full electronic search strategy for at least one database, including any limits used, such that it could be repeated.	10-11
Study selection	9	State the process for selecting studies (i.e., screening, eligibility, included in systematic review, and, if applicable, included in the meta-analysis).	12
Data collection process	10	Describe method of data extraction from reports (e.g., piloted forms, independently, in duplicate) and any processes for obtaining and confirming data from investigators.	12-13
Data items	11	List and define all variables for which data were sought (e.g., PICOS, funding sources) and any assumptions and simplifications made.	12-13
Risk of bias in individual studies	12	Describe methods used for assessing risk of bias of individual studies (including specification of whether this was done at the study or outcome level), and how this information is to be used in any data synthesis.	13-16
Summary measures	13	State the principal summary measures (e.g., risk ratio, difference in means).	16
Synthesis of results	14	Describe the methods of handling data and combining results of studies, if done, including measures of consistency (e.g., I^2) for each meta-analysis.	16-17

For peer review only - http://bmjopen.bmj.com/site/about/guidelines.xhtml

S2. PRISMA 2009 Checklist

Section/topic	#	Checklist item	Reported on page #
Risk of bias across studies	15	Specify any assessment of risk of bias that may affect the cumulative evidence (e.g., publication bias, selective reporting within studies).	N/A
Additional analyses	16	Describe methods of additional analyses (e.g., sensitivity or subgroup analyses, meta-regression), if done, indicating which were pre-specified.	17
**RESULTS**			
Study selection	17	Give numbers of studies screened, assessed for eligibility, and included in the review, with reasons for exclusions at each stage, ideally with a flow diagram.	17-18
Study characteristics	18	For each study, present characteristics for which data were extracted (e.g., study size, PICOS, follow-up period) and provide the citations.	19-23
Risk of bias within studies	19	Present data on risk of bias of each study and, if available, any outcome level assessment (see item 12).	23-24
Results of individual studies	20	For all outcomes considered (benefits or harms), present, for each study: (a) simple summary data for each intervention group (b) effect estimates and confidence intervals, ideally with a forest plot.	24-29
Synthesis of results	21	Present results of each meta-analysis done, including confidence intervals and measures of consistency.	24-28
Risk of bias across studies	22	Present results of any assessment of risk of bias across studies (see Item 15).	24
Additional analysis	23	Give results of additional analyses, if done (e.g., sensitivity or subgroup analyses, meta-regression [see Item 16]).	n/a
**DISCUSSION**			
Summary of evidence	24	Summarize the main findings including the strength of evidence for each main outcome; consider their relevance to key groups (e.g., healthcare providers, users, and policy makers).	29-31
Limitations	25	Discuss limitations at study and outcome level (e.g., risk of bias), and at review-level (e.g., incomplete retrieval of identified research, reporting bias).	32
Conclusions	26	Provide a general interpretation of the results in the context of other evidence, and implications for future research.	31-32
**FUNDING**			
Funding	27	Describe sources of funding for the systematic review and other support (e.g., supply of data); role of funders for the systematic review.	34

For peer review only - http://bmjopen.bmj.com/site/about/guidelines.xhtml

Downloaded from http://bmjopen.bmj.com/ on September 16, 2016 - Published by group.bmj.com

Open Access Protocol

BMJ Open

The effects of shifting tasks from pharmacy to non-pharmacy personnel for providing antiretroviral therapy to people living with HIV: a systematic review protocol

Nyanyiwe M Mbeye,[1,2] Tamara Kredo,[1] Charles S Wiysonge[1,2]

For peer review only

To cite: Mbeye NM, Kredo T, Wiysonge CS. The effects of shifting tasks from pharmacy to non-pharmacy personnel for providing antiretroviral therapy to people living with HIV: a systematic review protocol. *BMJ Open* 2016;**6**: e008195. doi:10.1136/ bmjopen-2015-008195

▸ Prepublication history for this paper is available online. To view these files please visit the journal online (http://dx.doi.org/10.1136/ bmjopen-2015-008195).

Received 15 March 2015
Revised 16 November 2015
Accepted 17 November 2015

[1]Cochrane South Africa, South African Medical Research Council, Tygerberg, South Africa
[2]Faculty of Medicine and Health Sciences, Centre for Evidence-based Health Care, Stellenbosch University, Cape Town, South Africa

Correspondence to
Dr Nyanyiwe M Mbeye; nyanyiwembeye@gmail.com

ABSTRACT

Introduction: Shifting selected antiretroviral therapy (ART) tasks from specialised healthcare workers to those with shorter or less formal training has been implemented in resource-limited settings to alleviate critical shortages of human resources for health. However, the specifics of shifting ART dispensing from pharmacy to non-pharmacy personnel have not been addressed in a systematic review, although this can potentially increase access to ART. We will assess the effects of shifting dispensing and distribution of ART and adherence assessment from pharmacy to non-pharmacy personnel in low and middle-income countries.

Methods and analysis: We will search PubMed, CENTRAL, EMBASE, WHO Global Health Library and relevant grey literature for eligible controlled trials. Two authors will screen the search output, select eligible studies, assess risk of bias and extract data from included studies, resolving discrepancies by discussion and consensus. We will perform meta-analysis using both fixed and random effects models, investigate clinical and statistical heterogeneity, and assess our confidence in the overall evidence using standard Cochrane methods, including GRADE.

Ethics and dissemination: Only secondary data will be included in this review and ethics approval is not required. We will disseminate the review findings in various scientific fora, including peer-reviewed journals. The findings may help to inform policy makers in defining the scope of work of healthcare workers, and global recommendations for shifting the dispensing and distribution of ART from pharmacy to non-pharmacy personnel.

Trial registration number: CRD42015017034.

Strengths and limitations of this study

- To our knowledge, this is the first published protocol of a systematic review that will investigate the effects of task shifting from pharmacy to non-pharmacy personnel for dispensing or distributing antiretroviral therapy to patients living with HIV.
- The protocol was written according to the PRISMA-P (Preferred Reporting Items for Systematic Review and Meta-Analysis Protocols) recommendations.
- The review findings may help to inform antiretroviral therapy guidelines by the WHO.
- The possible weakness of the planned review would be the limitations of included studies, for example, high risk of bias and heterogeneity of settings, designs and effects.

INTRODUCTION

Description of the condition

By March 2015, 15 million (40.7%) of the estimated 36.9 million people living with HIV (PLHIV) globally were receiving antiretroviral therapy (ART).[1] Combination ART is effective for reducing HIV related morbidity and mortality as well as preventing HIV transmission.[2] Initiating ART early in the course of HIV infection has been associated with better health outcomes, both at patient and population levels.[3 4] Scale-up of ART in low and middle income countries has averted more than 5 million deaths; however, bottlenecks preventing universal access to ART still exist. One challenge is the critical shortage of human resources for health (HRH), including for the delivery of essential HIV related pharmacy services.

The WHO recommends a minimum of one pharmacist per 2300 population, but most countries in low-resource settings such as sub-Saharan Africa have not yet met this target.[5] In addition to the absolute shortage, it is likely that there is an uneven distribution of pharmacists in such settings, as is the case with other specialist healthcare workers who

For peer review only - http://bmjopen.bmj.com/site/about/guidelines.xhtml

Downloaded from http://bmjopen.bmj.com/ on September 16, 2016 - Published by group.bmj.com

tend to concentrate in urban areas and the private sector, further aggravating the HRH shortage.[6] For instance in South Africa, which is home to the largest number of PLHIV in any country in the world, in 2010 only 24% of registered pharmacists worked in the public sector where 80% of the population received care.[7]

Description of the intervention

Studies and programme reports indicate that involvement of pharmacy personnel in HIV care results in improved patient outcomes. For instance, in the USA, the use of a multidisciplinary team approach with pharmacists assuming a central role in ART initiation, dispensing and adherence counselling improved treatment outcomes such as viral load, patient retention and medication adherence.[8]

The work of pharmacists includes supply management, dispensing and distributing medications, promoting adherence, identifying and preventing potential medication-related issues, and monitoring and reporting adverse events. In some settings, programmes have implemented alternative models of pharmacy services that shift selected tasks from pharmacy to non-pharmacy personnel. Such alternative models could potentially increase the number of health workers involved in ART distribution, adherence counselling and patient education, free more time for pharmacy personnel, support the integration of ART in primary care settings, minimise the number of facility visits for ART collection, and reduce pharmacy queue waiting times for patients.[9]

However, the specifics of shifting ART related tasks from pharmacy to non-pharmacy personnel have not yet been addressed in a systematic review. We therefore plan to synthesise the evidence for task shifting in pharmacy services, where non-pharmacy personnel undertake ART dispensing and distribution and medication adherence counselling. For this systematic review, pharmacy personnel will include both pharmacists and pharmacy technicians. Pharmacy technicians constitute an important part of the pharmacy workforce in low and middle-income countries: a survey of 26 low and middle-income countries in 2011 revealed that pharmacy technicians constitute 10% (Nigeria) to 70% (Pakistan) of the pharmacy workforce.[10]

How the intervention might work

Within the last decade, several high HIV burden countries adopted task shifting strategies where nurses and non-physician clinicians initiate and maintain ART.[11] Although this has undeniably expanded access to ART, it is also increasingly essential that long facility waiting times and frequent facility visits to collect ART are addressed to alleviate the burden of care, both for patients and healthcare providers.[12 13]

Recent studies in Uganda, Kenya and Mozambique have shown positive outcomes when non-health professionals (lay people) delivered ART at the community level.[14] In Mozambique the use of PLHIV for distributing ART, monitoring adherence, reporting outcomes and referring sick patients to health facilities yielded a retention rate of 97.5% among stable patients on ART.[14] In a cluster randomised trial in Uganda, the use of trained community health workers produced comparable results with facility-based ART programme in terms of patient retention, viral load suppression and mortality rate.[15] Similar findings were also obtained in Kenya and Uganda when lay providers were engaged in ART delivery.[15 16]

Task shifting has therefore been seen as an achievable solution to the critical human resource shortages for scale-up of ART.[17] While it is imperative to increase the rate of recruitment and training of health workers as well as improve working conditions to reduce attrition and emigration, the HIV pandemic requires a more urgent measure to address the critical skills shortage.[18] Such measures may include shifting selected tasks (including dispensing and distributing ART and adherence counselling) from pharmacy to non-pharmacy personnel. The task shifting could free time for pharmacy personnel to focus on more technical functions such as supply management and pharmacovigilance.

Why this review is important

Previous systematic reviews of task shifting for increasing ART access focused on clinical services where nurses and non-clinician physicians provide care.[11] Dependence on and shortages of pharmacists are also key constraints on ART expansion, but the specifics of task shifting for ART dispensing or distribution from pharmacy to non-pharmacy personnel have not been reviewed systematically. We will systematically review the scientific literature and assess the efficacy and safety of task shifting models that use non-pharmacy personnel in dispensing or distributing ART and assessing adherence to treatment of HIV infection.

OBJECTIVE

The aim of this review is to evaluate the efficacy and safety of shifting dispensing and distribution of ART as well as assessment of adherence from pharmacy to non-pharmacy personnel.

METHODS

This review protocol has been registered in the PROSPERO International Prospective Register of Systematic Reviews (http://www.crd.york.ac.uk/PROSPERO), registration number CRD42015017034.

Criteria for considering studies for this review

Types of studies

We will include randomised controlled trials (RCTs) and non-RCTs, irrespective of whether allocation to interventions occurred at the individual or cluster level.

Types of participants

Participants will be PLHIV receiving ART.

For peer review only - http://bmjopen.bmj.com/site/about/guidelines.xhtml

Downloaded from http://bmjopen.bmj.com/ on September 16, 2016 - Published by group.bmj.com

Types of interventions

We will include studies that evaluate the shifting of selected tasks from pharmacy personnel to non-pharmacy personnel. The selected tasks include dispensing and distribution of ART and adherence assessment. Pharmacy personnel will include both pharmacists and pharmacy technicians. Non-pharmacy personnel may include (but are not limited to) nurses, non-physician clinicians, and lay providers such as patient peer groups, community volunteers, PLHIV and community health committees.

Types of outcome measures

Primary outcomes

The primary outcome for this review is risk of death.

Secondary outcomes

Our secondary outcome measures include:

- Virological suppression
- Number of all-cause sick visits made to the health facility, including for adverse events
- Loss to follow-up
- Adherence to ART (as measured within the study, eg, pill counts, recall methods, digital methods)
- Acceptability to pharmacy personnel, non-pharmacy personnel and patients
- Harm, including error rates.

Search methods for identification of studies

We will perform a comprehensive and exhaustive search of electronic databases and conference proceedings in an attempt to identify all relevant studies available by the search date, regardless of language of publication or publication status (published, unpublished, in press or in progress).

Databases of peer-reviewed literature

We will search the following electronic databases, from 1 January 1996 to the search date:

- Cochrane Central Register of Controlled Trials (CENTRAL)
- Excerpta Medica Database (EMBASE)
- PubMed
- ISI Web of Science (Science Citation index)
- WHO Global Health Library, which includes references from AIM (AFRO), LILACS (AMRO/PAHO), IMEMR (EMRO), IMSEAR (SEARO) and WPRIM (WPRO).

Along with appropriate Medical Subject Heading (MeSH) terms and relevant keywords, we will use the Cochrane Highly Sensitive Search Strategy for identifying reports of randomised controlled trials in MEDLINE,[19] and the Cochrane validated strategies for identifying references relevant to HIV infection and AIDS. To identify other study designs, the RCT string will be omitted. The search strategy will be iterative in that references of included studies will be searched for additional references. See table 1 for our provisional search strategy for electronic databases.

Conference databases

We will search conference abstract archives on the web sites of the Conference on Retroviruses and Opportunistic Infections (CROI), the International AIDS Conference (IAC) and the International AIDS Society Conference on HIV Pathogenesis, Treatment and Prevention (IAS), for all available abstracts presented at these conferences from 1996 to the search date.

Searching other resources

We will also search the references of relevant articles as well as the WHO International Clinical Trials Registry Platform (ICTRP) and Clinicaltrials.gov. We will contact relevant experts or organisations who may be aware of additional studies in this field.

Data collection and analysis

We will base the methodology for data collection and analysis on the guidance provided in the Cochrane Handbook of Systematic Reviews of Interventions.[19]

Selection of studies for inclusion

Two authors will read and assess the abstracts of identified publications for potentially eligible studies. We will obtain full text articles for all abstracts judged by at least one of the two authors, to be potentially eligible. Two authors will independently inspect these potentially eligible publications to establish the relevance of the article to the review according to the pre-specified criteria regarding study design, participants, interventions and outcome measures.

Data extraction and management

Two authors will independently extract data into a pre-piloted data extraction form. The following characteristics will be extracted from each included study:

Study details: Complete citations of publications associated with the study, start and end dates, location, study design characteristics, type of facility involved, investigators, funding sources, recruitment, method of randomisation, sequence generation, method of allocation concealment, blinding of participants and personnel, blinding of outcome assessment, length of follow-up, losses to follow-up, withdrawals or drop-outs and other relevant details.

Details of the intervention: training of the cadre of health workers who were dispensing or distributing ART, what training or other support or supervision they received and other relevant details.

Details of participants: Trial inclusion and exclusion criteria, numbers of participants entering the trial, sex, clinical staging, CD4 count and other pertinent details.

Outcome details: Definitions of outcomes, details of how outcomes were assessed, numerators and denominators associated with each outcome, completeness of outcome data, effect estimates reported and other relevant outcome information.

For peer review only

For peer review only - http://bmjopen.bmj.com/site/about/guidelines.xhtml

Downloaded from http://bmjopen.bmj.com/ on September 16, 2016 - Published by group.bmj.com

Table 1 Proposed search strategy for electronic databases

ID	Search terms
PubMed	
#1	(task*[tiab] OR task-shifting[tiab] OR referr*[tiab] OR referral and consultation[mh] OR role*[tiab]) AND (health personnel [mh] OR doctor[tiab] OR doctors[tiab] OR clinician[tiab] OR clinicians[tiab] OR physician[tiab] OR physicians[tiab] OR “healthcare provider"[tiab] OR “healthcare providers"[tiab] OR “health care provider"[tiab] OR “health care providers"[tiab] OR pharmac*[tiab] OR apothecar*[tiab] OR chemist*[tiab] OR dispensar*[tiab])
#2	randomized controlled trial[pt] OR controlled clinical trial[pt] OR randomized controlled trials[MeSH] OR random allocation[MeSH] OR double-blind method[MeSH] OR single-blind method[MeSH] OR clinical trial[pt] OR clinical trials [MeSH] OR (“clinical trial"[tw]) OR ((singl*[tw] OR doubl*[tw] OR trebl*[tw] OR tripl*[tw]) AND (mask*[tw] OR blind*[tw])) OR random*[tw] OR research design[mh:noexp] OR prospective studies[MeSH] OR control*[tw] OR volunteer*[tw]) OR observational[tw] OR non-random*[tw] OR nonrandom*[tw] OR before after study[tw] OR time series[tw] OR cohort*[tw] OR cross-section*[tw] OR prospective*[tw] OR retrospective*[tw] OR research design[mh:noexp] OR follow-up studies [MeSH] OR longitud*[tw] OR evaluat*[tiab] OR pre-post[tw] OR (pre-test[tw] AND post-test[tw]) NOT (animals[MeSH] NOT human[MeSH])
#3	(HIV Infections[MeSH] OR HIV[MeSH] OR hiv[tiab] OR hiv-1[tiab] OR hiv-2*[tiab] OR hiv1[tiab] OR hiv2[tiab] OR hiv infect*[tiab] OR human immunodeficiency virus[tiab]OR human immune deficiency virus[tiab] OR human immuno-deficiency virus[tiab] OR human immune-deficiency virus[tiab] OR ((human immun*) AND(deficiency virus[tiab])) OR acquired immunodeficiency syndromes[tiab] OR acquired immune deficiency syndrome[tiab] OR acquired immuno-deficiency syndrome[tiab] OR acquired immune-deficiency syndrome[tiab] OR ((acquired immun*) AND (deficiency syndrome[tiab])) or “sexually transmitted diseases, viral”[mh]) OR HIV[tiab] OR HIV/AIDS[tiab] OR HIV-infected[tiab] OR HIV[title] OR HIV/AIDS[title] OR HIV-infected[title]
#4	(HAART[tiab] OR ART[tiab] OR cART[tiab] OR antiretroviral[tiab] OR anti-retroviral[tiab] OR anti-viral[tiab] OR antiviral [tiab] OR “Antiretroviral Therapy, Highly Active"[Mesh])
**#5**	#1 AND #2 AND #3 AND #4
Scopus	
	(HIV OR HIV/AIDS OR AIDS OR “HUMAN IMMUNODEFICIENCY” OR “ACQUIRED IMMUNODEFICIENCY)” AND TITLE-ABS-KEY (TASK-SHIFTING OR TASKSHIFTING OR (TASK* AND SHIFT*) OR TASK* OR (REFERR* AND (NURSE* OR PHARMAC*))) AND TITLE-ABS-KEY (ANTIRETROVIRAL OR ANTI-RETROVIRAL OR ART OR CART OR HAART) AND TITLE-ABS-KEY (RANDOM* OR RANDOMIZED OR RANDOMISED OR TRIAL OR COHORT* OR GROUP* OR COMPAR* OR OBSERVATIONAL OR PROSPECTIVE* OR RETROSPECTIVE* OR “SYSTEMATIC REVIEW” OR “META-ANALYSIS)”
Web of Science	
	(TS=(HIV OR HIV/AIDS OR AIDS OR “HUMAN IMMUNODEFICIENCY” OR “ACQUIRED IMMUNODEFICIENCY)” AND TS=(TASK-SHIFTING OR TASKSHIFTING OR (TASK* AND SHIFT*) OR TASK* OR (REFERR* AND (NURSE* OR PHARMAC*))) AND TS=(ANTIRETROVIRAL OR ANTI-RETROVIRAL OR ART OR CART OR HAART) AND TS= (RANDOM* OR RANDOMIZED OR RANDOMISED OR TRIAL OR COHORT* OR GROUP* OR COMPAR* OR OBSERVATIONAL OR PROSPECTIVE* OR RETROSPECTIVE* OR “SYSTEMATIC REVIEW” OR “META-ANALYSIS)”)
OR	
	(TI=(HIV OR HIV/AIDS OR AIDS OR “HUMAN IMMUNODEFICIENCY” OR “ACQUIRED IMMUNODEFICIENCY)” AND TI=(TASK-SHIFTING OR TASKSHIFTING OR (TASK* AND SHIFT*) OR TASK* OR (REFERR* AND (NURSE* OR PHARMAC*))) AND TI=(ANTIRETROVIRAL OR ANTI-RETROVIRAL OR ART OR cART OR HAART) AND TI= (RANDOM* OR RANDOMIZED OR RANDOMISED OR TRIAL OR COHORT* OR GROUP* OR COMPAR* OR OBSERVATIONAL OR PROSPECTIVE* OR RETROSPECTIVE* OR “SYSTEMATIC REVIEW” OR “META-ANALYSIS)”)
CENTRAL	
	HIV* OR HIV-1* OR HIV-2* OR HIV1 OR HIV2 OR HIV INFECT* OR HUMAN IMMUNODEFICIENCY VIRUS OR HUMAN IMMUNEDEFICIENCY VIRUS OR HUMAN IMMUNE-DEFICIENCY VIRUS OR HUMAN IMMUNO-DEFICIENCY VIRUS OR HUMAN IMMUN* DEFICIENCY VIRUS OR ACQUIRED IMMUNODEFICIENCY SYNDROME OR ACQUIRED IMMUNEDEFICIENCY SYNDROME OR ACQUIRED IMMUNO-DEFICIENCY SYNDROME OR ACQUIRED IMMUNE-DEFICIENCY SYNDROME OR ACQUIRED IMMUN* DEFICIENCY SYNDROME in Title, Abstract, Keywords and (TASK-SHIFTING OR TASKSHIFTING OR (TASK* AND SHIFT*) OR TASK* OR (REFERR* AND (NURSE* OR PHARMAC*))) in Title, Abstract, Keywords and ANTIRETROVIRAL OR ANTI-RETROVIRAL OR ART OR cART OR HAART in Title, Abstract, Keywords
WHO Global Health Library	
	(TASK-SHIFTING OR TASKSHIFTING OR (TASK* AND SHIFT*) OR TASK* OR (REFERR* AND (NURSE* OR PHARMAC*)) AND (HIV* OR human immunodeficiency) AND (antiretroviral OR anti-retroviral))) OR (HIV AND task-shifting) OR (HIV* AND task* AND shift*)

Assessment of risk of bias in included studies

We will assess the risk of bias in RCTs using the Cochrane risk of bias assessment tool for randomised studies.[19] For non-RCT studies, we will use the Cochrane Risk of Bias Assessment Tool for Non-Randomised Studies of Interventions (ACROBAT-NRSI).[20]

For peer review only - http://bmjopen.bmj.com/site/about/guidelines.xhtml

Downloaded from http://bmjopen.bmj.com/ on September 16, 2016 - Published by group.bmj.com

We will resolve any disagreements between the authors conducting duplicate independent screening of search outputs, assessments of study eligibility, extraction of data, and risk of bias assessment by discussion and consensus. Should this fail to resolve the differences, a third author will arbitrate.

Measures of effect

We will calculate and report risk ratios for dichotomous and time-to-event data and mean differences for continuous data with their 95% CIs.

Unit of analysis issues

The unit of analysis will be the individual study participant. Cluster-randomised trials will be included in meta-analyses only after adjustments are made for design effect. Design effects for cluster-randomised studies will be corrected by using standard procedures, using the formula: design effect=1+(m−1)r, where m is the average cluster size and r is the intra-cluster correlation coefficient.

Dealing with missing data

We will contact study authors if it is necessary to obtain data missing from published reports.

Assessment of heterogeneity

We will examine statistical heterogeneity between study results using the χ^2 test of homogeneity, with a significance α-level of 0.1. In addition, we will use the I^2 statistic to measure the amount of heterogeneity among the trials in each analysis. If we identify significant heterogeneity (ie, p<0.1), we will explore it by prespecified subgroup analysis. If heterogeneity persists, we will perform sensitivity analyses, report results separately and propose reasons for the observed heterogeneity.

Assessment of reporting biases

If any meta-analysis in our review includes 10 or more studies, we will assess the potential for publication bias using a funnel plot.[21] We will attempt to minimise the potential for publication bias through a comprehensive search of published and unpublished literature.

Data synthesis

We will conduct meta-analysis, if appropriate, using the Cochrane Review Manager software (RevMan [Computer program] The Nordic Cochrane Centre, *The Cochrane Collaboration, Copenhagen*, 2015; Version 5.3). If we find no significant statistical heterogeneity of effects, we will use the fixed effect method of meta-analysis. Otherwise, we will use the random effects model.

Subgroup analysis

In pooled results with significant statistical heterogeneity, we will explore the cause of the heterogeneity through subgroup analyses, with subgroups defined by type of intervention (eg, cadre of health provider), comparison group and region of study (eg, sub-Saharan Africa, Southeast Asia, etc).

Sensitivity analysis

We will conduct a sensitivity analysis to investigate the effect of excluding studies with high risk of bias, with a focus on bias introduced by inadequate allocation concealment, inadequate blinding of outcome assessment and substantial losses to follow-up.

Certainty of evidence

We will assess the certainty (or quality) of evidence using the Grading of Recommendations, Assessment, Development and Evaluation (GRADE) approach,[22] which defines the certainty of evidence for each outcome as "the extent of our confidence that the estimates of effect are correct".[19] The quality rating across studies has four levels: high, moderate, low and very low. Randomised trials are considered to be of high quality but can be downgraded for any of five reasons: risk of bias, indirectness of evidence, unexplained heterogeneity of effects, imprecision of effect estimates and high probability of publication bias. Similarly, observational studies are considered to be of low quality, but can be upgraded for any of three reasons. The quality level of a body of evidence can be increased if there is a large magnitude of effect, if all plausible confounding would reduce a demonstrated effect, and if there is a dose-response gradient.

Reporting of this review

The findings of this review will be presented in a number of ways. The study selection process will be summarised using a flow diagram, and if we identify 10 or more eligible studies, we will assess publication bias using funnel plots. Where appropriate, we will use risk of bias graphs, forest plots and GRADE summary of findings tables. The non-quantitative outcomes will be reported descriptively. We will provide tables of both included and excluded studies. We have prepared this protocol as recommended by the PRISMA-P (Preferred Reporting Items for Systematic Review and Meta-Analysis Protocols) guidelines[23] and will report the findings of the review as recommended by the PRISMA statement.[24]

ETHICS AND DISSEMINATION

Since systematic reviews do not directly involve human participants, they do not require ethical clearance.[25] We will provide the findings of this review to the WHO, with the hope that they may guide policy recommendations of this normative agent regarding the shifting of ART dispensing or distribution from pharmacy to non-pharmacy personnel. Although the majority of national programmes in low and middle-income countries have adopted task shifting in ART care at different levels,

For peer review only - http://bmjopen.bmj.com/site/about/guidelines.xhtml

Downloaded from http://bmjopen.bmj.com/ on September 16, 2016 - Published by group.bmj.com

there has been no global policy to guide the practice for task shifting from pharmacy to non-pharmacy personnel. We will also publish the findings of the systematic review in a peer-reviewed journal.

Twitter Follow Charles Wiysonge at @CharlesShey

Acknowledgements The authors acknowledge the Editor and the referees for critical and constructive comments on earlier versions of this manuscript. The authors would also like to acknowledge the various contributions of Eyerusalem Negussie.

Contributors NMM led the development of the protocol, wrote the first draft, coordinated and integrated comments from co-authors and approved the final version for publication. TK and CSW conceived the study, critically revised successive drafts of the manuscript, and approved the final version for publication. CSW is the guarantor of the manuscript.

Funding Funding for preparing this review was provided by the Department of HIV/AIDS at the WHO Headquarters in Geneva, Switzerland. The authors would like to thank Hacsi Horvath (Global Health Sciences, University of California, San Francisco, USA) for his assistance in developing the search strategy for electronic databases. Neither the authors' institutions nor the funder played a role in preparing the manuscript and the views expressed therein are solely those of the authors.

Competing interests None declared.

Provenance and peer review Not commissioned; externally peer reviewed.

Open Access This is an Open Access article distributed in accordance with the terms of the Creative Commons Attribution (CC BY 4.0) license, which permits others to distribute, remix, adapt and build upon this work, for commercial use, provided the original work is properly cited. See: http://creativecommons.org/licenses/by/4.0/

REFERENCES

1. UNAIDS. How AIDS changed everything—MDG 6: 15 years, 15 lessons of hope from the AIDS response. https//:www.unaids.org/en/resources/documents/2015/MDG6_15years-15lessonsfromtheAIDSresponse (accessed 22 Sep 2015).
2. Palella FJ, Delaney KM, Moorman AC, *et al*. Declining morbidity and mortality among patients with advanced human immunodeficiency virus infection. HIV Outpatient Study Investigators. *N Engl J Med* 1998;338:853–60.
3. Anglemyer A, Rutherford GW, Horvath T, *et al*. Antiretroviral therapy for prevention of HIV transmission in HIV-discordant couples. *Cochrane Database Syst Rev* 2013;4:CD009153.
4. Ford N, Calmy A, Mills EJ. The first decade of antiretroviral therapy in Africa. *Global Health* 2011;7:33.
5. King RC, Fomundam HN. Remodeling pharmaceutical care in sub-Saharan Africa (SSA) amidst human resources challenges and the HIV/AIDS pandemic. *Int J Health Plann Manage* 2010;25:30–48.
6. WHO. *Increasing access to health workers in remote and rural areas through improved retention: global policy recommendations*. Geneva, Switzerland: World Health Organisation, 2010.
7. SAPC. Pharmacy Human Resources data. https//:www.healthlink.org.za/healthstats/283/data (accessed 15 Aug 2015).
8. Saberi P, Dong BJ, Johnson MO, *et al*. The impact of HIV clinical pharmacists on HIV treatment outcomes: a systematic review. *Patient Prefer Adherence* 2012;6:297–322.
9. Tseng A, Foisy M, Hughes CA, *et al*. Role of pharmacist in caring for patients with HIV/AIDS: clinical practice guidelines. *Can J Hosp Pharm* 2012;65:125–45.
10. WHO. *The world of medicines situation 2011: pharmaceutical human resources*. Geneva, Switzerland: World Health Organisation, 2011.
11. Kredo T, Adeniyi FB, Bateganya M, *et al*. Task shifting from doctors to non-doctors for initiation and maintenance of antiretroviral therapy. *Cochrane Database Syst Rev* 2014;7:CD007331.
12. Kitahata MM, Koepsell TD, Deyo RA, *et al*. Physicians experience with the acquired immunodeficiency syndrome as a factor in patients' survival. *N Engl J Med* 1996;334:701–6.
13. Landon BE, Wilson IB, McInnes K, *et al*. Physician specialization and the quality of care for human immunodeficiency virus infection. *Arch Intern Med* 2005;165:1133–9.
14. Decroo T, Telfer B, Biot M, *et al*. Distribution of antiretroviral treatment through self-forming groups of patients in Tete province, Mozambique. *J Acquir Immune Defic Syndr* 2011;56:e39–44.
15. Kipp W, Konde-Lule J, Rubaale T, *et al*. Comparing antiretroviral treatment outcomes between a prospective community-based and hospital-based cohort of HIV patients in rural Uganda. *BMC Int Health Hum Rights* 2011;11(Suppl 2):S12.
16. Selke HM, Kimaiyo S, Sidle JE, *et al*. Task-shifting of antiretroviral delivery from health care workers to persons living with HIV/AIDS: clinical outcomes of a community-based program in Kenya. *J Acquir Immune Defic Syndr* 2010;55:483–90.
17. Callaghan M, Ford N, Schneider H. A systematic review of task-shifting for HIV treatment and care in Africa. *Hum Resour Health* 2010;8:8.
18. Laurant M, Reeves D, Hermens R, *et al*. Substitution of doctors by nurses in primary care. *Cochrane Database Syst Rev* 2005;CD001271.
19. Higgins JPT, Green S, eds. *Cochrane handbook for systematic reviews of interventions*. Wiley-Blackwell, 2008.
20. Cochrane Collaboration. A Cochrane Risk of Bias Assessment Tool: for Non-Randomized studied of Interventions (ACROBAT-NRSI). https://www.riskofbias.info/ (accessed 30 Oct 2015).
21. Egger M, Davey Smith G, Schneider M, *et al*. Bias in meta-analysis detected by a simple, graphical test. *BMJ* 1997;315:629–34.
22. Guyatt G, Oxman AD, Akl EA, *et al*. GRADE guidelines: 1. Introduction-GRADE evidence profiles and summary of findings tables. *J Clin Epidemiol* 2011;64:383–94.
23. Shamseer L, Moher D, Clarke M, *et al*; PRISMA-P Group. Preferred reporting items for systematic review and meta-analysis protocols (PRISMA-P). *BMJ* 2015;349:g7647.
24. Liberati A, Altman DG, Tetzlaff J, *et al*. The PRISMA Statement for reporting systematic reviews and meta-analyses of studies that evaluate healthcare interventions: explanation and elaboration. *BMJ* 2009;339:b2700.
25. Emanuel EJ, Wendler D, Killen J, *et al*. What makes clinical research in developing countries ethical? The benchmarks of ethical research. *J Infect Dis* 2004;189:930–7.

For peer review only

Downloaded from http://bmjopen.bmj.com/ on September 16, 2016 - Published by group.bmj.com

BMJ Open

The effects of shifting tasks from pharmacy to non-pharmacy personnel for providing antiretroviral therapy to people living with HIV: a systematic review protocol

Nyanyiwe M Mbeye, Tamara Kredo and Charles S Wysonge

BMJ Open 2016 6:
doi: 10.1136/bmjopen-2015-008195

Updated information and services can be found at:
http://bmjopen.bmj.com/content/6/3/e008195

These include:

References This article cites 18 articles, 4 of which you can access for free at:
http://bmjopen.bmj.com/content/6/3/e008195#BIBL

Open Access This is an Open Access article distributed in accordance with the terms of the Creative Commons Attribution (CC BY 4.0) license, which permits others to distribute, remix, adapt and build upon this work, for commercial use, provided the original work is properly cited. See:
http://creativecommons.org/licenses/by/4.0/

Email alerting service Receive free email alerts when new articles cite this article. Sign up in the box at the top right corner of the online article.

Topic Collections Articles on similar topics can be found in the following collections

- Evidence based practice (557)
- Health services research (1106)
- HIV AIDS (159)

Notes

To request permissions go to:
http://group.bmj.com/group/rights-licensing/permissions

To order reprints go to:
http://journals.bmj.com/cgi/reprintform

To subscribe to BMJ go to:
http://group.bmj.com/subscribe/

For peer review only

BMJ Open

Shifting tasks from pharmacy to non-pharmacy personnel for providing antiretroviral therapy to people living with HIV: A systematic review and meta-analysis

Journal:	*BMJ Open*
Manuscript ID	bmjopen-2016-015072.R1
Article Type:	Research
Date Submitted by the Author:	11-Apr-2017
Complete List of Authors:	MBEYE, NYANYIWE; Stellenbosch University, Centre for Evidence Based Health Care; College of Medicine, School of Public Health and Family Medicine<br>Adetokunboh, Olatunji; Stellenbosch University, Centre for Evidence Based Health Care; South African Medical Research Council, Cochrane South Africa<br>Negussie, Eyerusalem; World Health Organisation, HIV/AIDS<br>Kredo, Tamara; South African Medical Research Council, Cochrane South Africa; Stellenbosch University Faculty of Medicine and Health Sciences, Division of clinical Pharmacology<br>Wiysonge, Charles; Stellenbosch University, Centre for Evidence-Based Health Care; South African Medical Research Council, Cochrane South Africa
**Primary Subject Heading**:	Evidence based practice
Secondary Subject Heading:	Health services research, HIV/AIDS, Evidence based practice
Keywords:	Task shifting, HIV, antiretroviral therapy, dispensing, distribution, pharmacy personnel

SCHOLARONE™ Manuscripts

Shifting tasks from pharmacy to non-pharmacy personnel for providing antiretroviral therapy to people living with HIV: A systematic review and meta-analysis

Nyanyiwe M. Mbeye[1,2&5*], Olatunji Adetokunboh[1&2], Eyerusalem Negussie[3], Tamara Kredo[1&4], Charles S. Wiysonge[1&2],

[1] Cochrane South Africa, South African Medical Research Council, Cape Town, South Africa.

[2] Centre for Evidence-based Health Care, Faculty of Medicine and Health Sciences, Stellenbosch University, Cape Town, South Africa.

[3] Department of HIV/AIDS, World Health Organization, Geneva, Switzerland

[4] Division of Clinical Pharmacology, Faculty of Medicine and Health Sciences, Stellenbosch University, Cape Town, South Africa.

[5]College of Medicine, University of Malawi, Blantyre, Malawi.

***Correspondence author:** Dr Nyanyiwe M. Mbeye (nyanyiwembeye@gmail.com), Telephone Number: +265 888356338

For peer review only

Abstract

Objectives

Lay people or non-pharmacy health workers with training could dispense antiretroviral therapy (ART) in resource-constrained countries; freeing up time for pharmacists to focus on more technical tasks. We assessed the effectiveness of such task-shifting in low and middle-income countries.

Method

We conducted comprehensive searches of peer-reviewed and grey literature. Two authors independently screened search outputs, selected controlled trials, extracted data and resolved discrepancies by consensus. We performed random-effects meta-analysis and assessed certainty of evidence using GRADE.

Results

Three studies with 1993 participants met the inclusion criteria, including two cluster trials conducted in Kenya and Uganda and an individually randomised trial conducted in Brazil. Pooling data we found that there may be no differences between pharmacy and non-pharmacy models of dispensing ART on mortality (RR 1.86, 95% confidence interval 0.44 to 7.95), virological failure (RR 0.92, 0.73 to 1.15) and loss to follow-up (RR 1.13, 0.68 to 1.91). We found some evidence that costs may be reduced for the patient and health system when task-shifting is undertaken. The overall quality of the evidence was low for all outcomes due to serious indirectness (trials compared complex interventions involving, but not limited to, pharmacy to non-pharmacy personnel) and imprecision (effects ranged from appreciable benefit to harm).

For peer review only

Conclusions

Health outcomes may be similar when ART dispensing or distribution is shifted from pharmacy to non-pharmacy personnel. However, the low-certainty evidence implies a high likelihood that further research may find the effects of the intervention to be substantially different from our findings. If resource-constrained countries decide to shift ART dispensing and distribution from pharmacy to non-pharmacy personnel, this should be accompanied by robust monitoring and impact evaluation.

Key words: Task-shifting, HIV, antiretroviral therapy, dispensing, distribution, and pharmacy personnel.

Strengths and limitations

- To our knowledge, this is the first published systematic review reporting the effects of task shifting from pharmacy to non-pharmacy personnel for dispensing or distributing antiretroviral therapy to patients living with HIV.
- The review was written according to the PRISMA (Preferred Reporting Items for Systematic Review and Meta-Analysis) Recommendations for reporting systematic reviews.
- The review findings may help to inform antiretroviral therapy guidelines by the World Health Organisation (WHO).
- The possible weakness of the review would be the limitations of included studies, for example, high risk of bias and heterogeneity of settings, designs and effects.

For peer review only

Background

Description of the condition

By March 2015, 15 million (40.7%) of the estimated 36.9 million people living with HIV (PLHIV) globally, were receiving antiretroviral therapy (ART) in low and middle-income countries [1]. Combination ART is effective for reducing HIV-related morbidity and mortality as well as preventing HIV transmission [2]. Initiating ART early in the course of HIV infection has been associated with better health outcomes, both at patient and population levels [3 4]. Scale up of ART in low and middle-income countries has averted more than 5 million deaths; however, bottlenecks preventing universal access to ART still exist. One challenge is the critical shortage of human resources for health (HRH), including for delivery of essential pharmacy services.

As for pharmacists, although the World Health Organization (WHO) recommends a minimum of one pharmacist per 2300 population [5], most countries in low-resource settings such as sub-Saharan Africa do not meet this target. In addition to the absolute shortage, it is likely that there is an uneven distribution of pharmacists in such settings, as is the case with other specialist healthcare workers who tend to concentrate in urban areas and the private sector, further aggravating the HRH shortage. For instance in South Africa, which is home to the largest number of PLHIV in any country worldwide, in 2010 only 24% of registered pharmacists worked in the public sector where 80% of the population received care [6].

Description of the intervention

Studies and programmes report that involvement of pharmacy personnel in HIV care results in improved patient outcomes [7]. For instance, in the United States of America (USA), the use of a multidisciplinary team approach with pharmacists assuming a

For Peer review only

central role in the initiation, dispensing and adherence counselling improved treatment outcomes such as viral load, patient retention and medication adherence [8]. The work of pharmacists includes supply management, dispensing and distributing medications, promoting adherence, identifying and preventing potential medication-related issues (such as over dosage, sub-therapeutic dosage, adverse drug reactions, medication errors and untreated indications), and monitoring and reporting adverse drug events [9 10]. In some settings, programmes have implemented alternative models of pharmacy services that shift selected tasks from pharmacy to non-pharmacy personnel. Such alternative models could potentially increase the number of health workers involved in ART distribution, adherence counselling and patient education; free more time for pharmacy personnel; support the integration of ART in primary care settings; minimise the number of facility visits for ART collection; and, reduce pharmacy queue waiting times for patients.

The specifics of shifting ART-related tasks from pharmacy personnel have not been addressed in a systematic review. Available systematic reviews on task shifting focus on clinical services where nurses and non-physician clinicians provide care comparable to physicians [11]. We therefore synthesised the evidence for task shifting in pharmacy personnel services, where non-pharmacy personnel undertake ART distribution and medication adherence counselling. Table 1 provides definitions for the pharmacy functions distribution and dispensing as they have been used in this review. However, these definitions seem to overlap at facility level.

Table 1: Definitions and descriptions of dispensing and distribution of medications

Term	Definition
Dispensing	• Dispensing is a controlled act that authorises one to select, prepare and provide stock medication that has been prescribed to a patient or client (or his/her representative) for

For peer review only

	administration at a later time[12] . • To dispense is to prepare and supply to a patient a course of therapy on the basis of a prescription[13] .. • Dispensing is preparation and distribution of a course of therapy to a patient, with appropriate instructions based on a prescription [13].
Distribution	• At facility level, drug distribution mainly refers to drugs dispensed by licensed practitioners, such as nurses, doctors, pharmacists or pharmacy assistants and collected by a patient. • At community level, drug distribution refers to trained lay people collecting pre-packed medications from the facility and delivering them to HIV patients in the community [12].

How task shifting using non-pharmacy personnel might work

Within the last decade, several high HIV burden countries adopted task shifting strategies where nurses and non-physician clinicians initiate and maintain ART. Although this has undeniably expanded access to ART, it is also increasingly essential that long facility waiting times and frequent facility visits to collect ART are addressed to alleviate the burden of care, both for patients and healthcare providers [9 10].

Recent studies in Uganda, Kenya and Mozambique have shown positive outcomes when non-health professionals (lay people) delivered ART at the community level [14]. In Mozambique, the use of PLHIV for distributing ART, monitoring adherence, reporting outcomes and referring sick patients to health facilities yielded a retention rate of 97.5% among stable patients on ART [14]. In a cluster randomised trial in Uganda, the use of community health workers produced comparable results with the facility-based ART programme in terms of patient retention, viral load suppression and mortality rate [15]. Similar findings were also obtained in Kenya and some other parts of Uganda when lay providers were engaged in ART delivery [15 16].

For peer review only

Task shifting has therefore been seen as an achievable solution to the critical human resource shortages affecting scale up of ART [17]. While it is imperative to increase the rate of recruitment and training of health workers as well as improve working conditions to reduce attrition and emigration, the HIV pandemic requires that all possible options are considered to address the critical skills shortage [18]. Such measures may include shifting selected tasks from pharmacy to non-pharmacy personnel, particularly for patients stable on their ART. Task-shifting allows more time for pharmacy personnel to focus on more technical functions such as supply management, pharmacovigilance and patient consultation.

Why this review is important

Dependence on and shortage of pharmacists are key constraints on ART expansion, but the specifics of task shifting for ART distribution from pharmacy to non-pharmacy personnel have not been reviewed systematically. Previous systematic reviews of task shifting for increasing ART access focused on clinical services where nurses and non-physician clinicians provide care. We systematically reviewed the scientific literature and assessed the efficacy of task-shifting models that use non-pharmacy and pharmacy personnel in distributing ART and assessing adherence to treatment of HIV infection.

Objective

The aim of this review was to evaluate the efficacy and safety of shifting pharmacy related tasks, including ART distribution and adherence assessment, from pharmacy to non-pharmacy personnel.

For peer review only - http://bmjopen.bmj.com/site/about/guidelines.xhtml

Methods

The review title was registered in the PROSPERO International Prospective Register of Systematic reviews (http://www.crd.york.ac.uk/PROSPERO), registration number CRD42015017034. The protocol was published in the BMJ Open [19].

Criteria for considering studies for this review

Types of studies: We included randomised controlled trials (RCTs) conducted in low and middle-income countries.

Types of participants*:* Eligible participants were people living with HIV receiving ART.

Types of interventions: We included studies that evaluated shifting of selected tasks from pharmacy to non-pharmacy personnel. The selected tasks include distribution, dispensing of ART as shown in Table 1 and adherence to ART. Pharmacy personnel included pharmacists and pharmacy technicians while non-pharmacy personnel included patient peer groups, community volunteers, people living with HIV, community health committees, nurses, physicians and non-physician clinicians.

For peer review only

Types of outcome measures*:* The primary outcome for this review is risk of death. Secondary outcomes include virological suppression; number of all-cause sick visits made to the health facility including adverse events; loss to follow-up; adherence to ART (as measured within the study, e.g. pill counts, recall methods, digital methods, self report); acceptability to pharmacy personnel, non-pharmacy personnel, patients; cost and harm including rates of errors.

Search methods for identification of studies

We performed a comprehensive search of electronic databases and conference proceedings to identify all relevant studies available up until 10 May 2016, regardless of language of publication or publication status (published, unpublished, in press and in progress). We searched the following electronic databases for studies indexed by the search date: Cochrane Central Register of Controlled Trials (CENTRAL), Excerpta Medica Database (EMBASE), PubMed, ISI Web of Science (Science Citation index), and World Health Organization (WHO) Global Health Library.

We used appropriate Medical Subject Heading (MeSH) terms, relevant keywords, and validated terms for identifying reports of randomised controlled trials [20]. We provide the details of the search strategy for each electronic database in Table 2. We also searched conference abstract archives on the websites of the Conference on Retroviruses and Opportunistic Infections (CROI), the International AIDS Conference (IAC), and the International AIDS Society Conference on HIV Pathogenesis, Treatment and Prevention (IAS), for all available abstracts presented at these conferences up to 10 May 2016. We contacted experts and organisations for additional studies in the field.

For peer review only

Table 2: Search strategy for electronic databases

ID	Search terms
**PubMed**	
#1	(task*[tiab] OR task-shifting[tiab] OR referr*[tiab] OR referral and consultation[mh] OR role*[tiab]) AND (health personnel[mh] OR doctor[tiab] OR doctors[tiab] OR clinician[tiab] OR clinicians[tiab] OR physician[tiab] OR physicians[tiab] OR "healthcare provider"[tiab] OR "healthcare providers"[tiab] OR "health care provider"[tiab] OR "health care providers"[tiab] OR pharmac*[tiab] OR apothecar*[tiab] OR chemist*[tiab] OR dispensar*[tiab])
#2	randomized controlled trial[pt] OR controlled clinical trial[pt] OR randomized controlled trials[MeSH] OR random allocation[MeSH] OR double-blind method[MeSH] OR single-blind method[MeSH] OR clinical trial[pt] OR clinical trials[MeSH] OR ("clinical trial"[tw]) OR ((singl*[tw] OR doubl*[tw] OR trebl*[tw] OR tripl*[tw]) AND (mask*[tw] OR blind*[tw])) OR random*[tw] OR research design[mh:noexp] OR prospective studies[MeSH] OR control*[tw] OR volunteer*[tw]) OR observational[tw] OR non-random*[tw] OR nonrandom*[tw] OR before after study[tw] OR time series[tw] OR cohort*[tw] OR cross-section*[tw] OR prospective*[tw] OR retrospective*[tw] OR research design[mh:noexp] OR follow-up studies[MeSH] OR longitud*[tw] OR evaluat*[tiab] OR pre-post[tw] OR (pre-test[tw] AND post-test[tw]) NOT (animals[MeSH] NOT human[MeSH])
#3	(HIV Infections[MeSH] OR HIV[MeSH] OR hiv[tiab] OR hiv-1[tiab] OR hiv-2*[tiab] OR hiv1[tiab] OR hiv2[tiab] OR hiv infect*[tiab] OR human immunodeficiency virus[tiab]OR human immune deficiency virus[tiab] OR human immuno-deficiency virus[tiab] OR human immune-deficiency virus[tiab] OR ((human immun*) AND(deficiency virus[tiab])) OR acquired immunodeficiency syndromes[tiab] OR acquired immune deficiency syndrome[tiab] OR acquired immuno-deficiency syndrome[tiab] OR acquired immune-deficiency syndrome[tiab] OR ((acquired immun*) AND (deficiency syndrome[tiab])) or "sexually transmitted diseases, viral"[mh]) OR HIV[tiab] OR HIV/AIDS[tiab] OR HIV-infected[tiab] OR HIV[title] OR HIV/AIDS[title] OR HIV-infected[title]
#4	(HAART[tiab] OR ART[tiab] OR cART[tiab] OR antiretroviral[tiab] OR anti-retroviral[tiab] OR anti-viral[tiab] OR antiviral[tiab] OR "Antiretroviral Therapy, Highly Active"[Mesh])
**#5**	#1 AND #2 AND #3 AND #4
**Scopus**	
	(HIV OR HIV/AIDS OR AIDS OR "HUMAN IMMUNODEFICIENCY" OR "ACQUIRED IMMUNODEFICIENCY") AND TITLE-ABS-KEY (TASK-SHIFTING OR TASKSHIFTING OR (TASK* AND SHIFT*) OR TASK* OR (REFERR* AND (NURSE* OR PHARMAC*))) AND TITLE-ABS-KEY (ANTIRETROVIRAL OR ANTI-RETROVIRAL OR ART OR CART OR HAART) AND TITLE-ABS-KEY (RANDOM* OR RANDOMIZED OR RANDOMISED OR TRIAL OR COHORT* OR

For peer review only

	GROUP* OR COMPAR* OR OBSERVATIONAL OR PROSPECTIVE* OR RETROSPECTIVE* OR "SYSTEMATIC REVIEW" OR "META-ANALYSIS")
**Web of Science**	
	(TS=(HIV OR HIV/AIDS OR AIDS OR "HUMAN IMMUNODEFICIENCY" OR "ACQUIRED IMMUNODEFICIENCY") AND TS=(TASK-SHIFTING OR TASKSHIFTING OR (TASK* AND SHIFT*) OR TASK* OR (REFERR* AND (NURSE* OR PHARMAC*))) AND TS=(ANTIRETROVIRAL OR ANTI-RETROVIRAL OR ART OR CART OR HAART) AND TS=(RANDOM* OR RANDOMIZED OR RANDOMISED OR TRIAL OR COHORT* OR GROUP* OR COMPAR* OR OBSERVATIONAL OR PROSPECTIVE* OR RETROSPECTIVE* OR "SYSTEMATIC REVIEW" OR "META-ANALYSIS")) <br>**OR** <br>(TI=(HIV OR HIV/AIDS OR AIDS OR "HUMAN IMMUNODEFICIENCY" OR "ACQUIRED IMMUNODEFICIENCY") AND TI=(TASK-SHIFTING OR TASKSHIFTING OR (TASK* AND SHIFT*) OR TASK* OR (REFERR* AND (NURSE* OR PHARMAC*))) AND TI=(ANTIRETROVIRAL OR ANTI-RETROVIRAL OR ART OR cART OR HAART) AND TI=(RANDOM* OR RANDOMIZED OR RANDOMISED OR TRIAL OR COHORT* OR GROUP* OR COMPAR* OR OBSERVATIONAL OR PROSPECTIVE* OR RETROSPECTIVE* OR "SYSTEMATIC REVIEW" OR "META-ANALYSIS"))
**CENTRAL**	
	HIV* OR HIV-1* OR HIV-2* OR HIV1 OR HIV2 OR HIV INFECT* OR HUMAN IMMUNODEFICIENCY VIRUS OR HUMAN IMMUNEDEFICIENCY VIRUS OR HUMAN IMMUNE-DEFICIENCY VIRUS OR HUMAN IMMUNO-DEFICIENCY VIRUS OR HUMAN IMMUN* DEFICIENCY VIRUS OR ACQUIRED IMMUNODEFICIENCY SYNDROME OR ACQUIRED IMMUNEDEFICIENCY SYNDROME OR ACQUIRED IMMUNO-DEFICIENCY SYNDROME OR ACQUIRED IMMUNE-DEFICIENCY SYNDROME OR ACQUIRED IMMUN* DEFICIENCY SYNDROME in Title, Abstract, Keywords and (TASK-SHIFTING OR TASKSHIFTING OR (TASK* AND SHIFT*) OR TASK* OR (REFERR* AND (NURSE* OR PHARMAC*))) in Title, Abstract, Keywords and ANTIRETROVIRAL OR ANTI-RETROVIRAL OR ART OR cART OR HAART in Title, Abstract, Keywords
**WHO Global Health Library**	
	(TASK-SHIFTING OR TASKSHIFTING OR (TASK* AND SHIFT*) OR TASK* OR (REFERR* AND (NURSE* OR PHARMAC*)) AND (HIV* OR human immunodeficiency) AND (antiretroviral OR anti-retroviral))) OR (HIV AND task-shifting) OR (HIV* AND task* AND shift*)

For peer review only

Data collection and analysis

We based the methodology for data collection and analysis on the guidance of Cochrane Handbook of Systematic Reviews of Interventions [20].

Selection of studies for inclusion

Two review authors Nyanyiwe M. Mbeye (NMM) and Ola Adetokunboh (OA) read the titles, abstracts and descriptor terms of the downloaded citations to identify potentially eligible reports. We obtained full-text articles for all citations identified as potentially eligible by at least one of the two authors. The two authors independently inspected potentially eligible publications to establish the relevance of the article to the review according to the pre-specified criteria regarding study designs, participants, interventions, and comparisons and outcome measures. Differences between the two authors were discussed with a third author Charles Shey Wiysonge (CSW) or Tamara Kredo (TK) and resolved by consensus.

Data extraction and management

Two review authors (NMM and OA) independently extracted data onto standardised, pre-piloted data extraction forms. The following characteristics were extracted from each included study:

- **Study details:** Complete citations of publications associated with the study, start and end dates, location, study design characteristics, type of facility involved, investigators, funding sources, recruitment, method of randomisation, sequence generation, method of allocation concealment, blinding of participants and

For peer review only

personnel, blinding of outcome assessors, length of follow up, losses to follow up, withdrawals or drop-outs and other relevant details.

- **Details of the intervention:** details of the cadre of health worker who was dispensing or distributing ART, what training or other support or supervision they received and other relevant details.
- **Details of participants:** Trial inclusion and exclusion criteria, numbers of participants entering the trial, sex, clinical staging, CD4 count and other pertinent details.
- **Outcome details:** Definitions of outcomes, details of how outcomes were assessed, numerators and denominators associated with each outcome, completeness of outcome data, effect estimates reported and other relevant outcome information.

Assessment of risk of bias in included controlled trials

Two review authors (NMM and OA) independently assessed the risk of bias in each study using the Cochrane risk of bias tool which measures risk of bias in controlled trials across seven domains: sequence generation, allocation concealment, blinding of participants and personnel, blinding of outcome assessors, completeness of outcome data, selective outcome reporting and other potential biases [20].

Sequence generation (checking for selection bias)

We considered a study as having a low risk of selection bias due to sequence generation if investigators described a random component in the sequence-generation process, such as the use of random number tables; coin tossing, card or envelope shuffling. We classified a study as having a high risk of bias if investigators described a non-random component in the sequence-generation process, such as the use of date

For peer review only

of births, day of the week, or hospital or clinic record numbers. Where there was insufficient information to permit judgment about the sequence generation process, we classified the study as having unclear risk of bias.

Allocation concealment (checking for selection bias)

We classified the study as having a low risk due to allocation concealment if participants and investigators enrolling participants could not foresee assignment (e.g. central allocation; or sequentially numbered, opaque, sealed envelopes). A study was classified as having a high risk of selection bias if participants and investigators could foresee assignment (e.g. an open, random-allocation schedule; a list of non-random numbers; or use of unsealed, non-opaque, or not sequentially numbered envelopes). We assigned a study as having an unclear risk of selection bias when the study reported insufficient information to permit judgment of the allocation concealment or the methods used to conceal allocation were not described.

Blinding (checking for performance and detection bias)

We determined that a study was at low risk of performance bias when there was blinding of the participants and key study personnel, and it was unlikely that the blinding could have been broken or there was no blinding in the situation but non-blinding was unlikely to introduce bias. A study with no blinding or incomplete blinding when the outcome was likely to be influenced by lack of blinding was classified as having a high risk of performance bias. In the absence of sufficient information to permit judgment of adequacy of the blinding of participants and investigators, the study was classified as having unclear risk of performance bias.

Regarding detection bias, we determined that a study was at low risk of bias when there was blinding of outcome assessors. We classified a study as having a high risk of detection bias if there was no blinding or the outcome was likely to be influenced

For peer review only

by lack of blinding of outcome assessors. In the absence of sufficient information to permit judgment of adequacy of the blinding of outcome assessment, the study was classified as having unclear risk of detection bias.

Incomplete outcome data (checking for attrition bias)

We classified a study as having a low risk of attrition bias if there was no missing outcome data, reasons for missing outcome data were unlikely to be related to the outcome, or the proportion of missing outcome data was balanced across groups. We classified a study as having a high risk of attrition bias if there was imbalance in proportions lost to follow-up across groups and the reason for missing outcome data was likely to be related to the outcome. When there was insufficient reporting of attrition or exclusions, we classified the study as having an unclear risk of attrition bias.

Selective reporting (checking for reporting bias)

We determined a study as having a low risk of reporting bias if a protocol was available and primary outcomes in the final trial report corresponded closely to those presented in the protocol. High risk of selective reporting was considered to be present when the primary outcomes differed between the protocol and final trial report. When no trial protocol was available or there was insufficient reporting to determine if selective reporting was present, we classified the study as having an unclear risk of reporting bias.

Checking for other forms of bias

We classified a study as having a low risk of other forms of bias, when there was no evidence of bias from other sources. A study was classified as having a high risk from other sources when potential bias was present from other sources (e.g. early stopping of trial, fraudulent activity, financial conflicts of interest). We assigned a study as

For peer review only

having an unclear risk of bias if insufficient information was available to permit an adequate judgment of presence of other forms of bias.

Measures of effect

We calculated and presented summary statistics for the risk ratio (RR) for dichotomous outcomes and the weighted-mean difference for continuous outcomes, with their 95% confidence intervals (CI).

Unit of analysis issues

The unit of analysis was the individual study participant for all the trials.

Dealing with missing data

We contacted study authors for one of the included trials to obtain information on the composition of health professionals in the control groups involved in dispensing ART in order to establish whether pharmacy personnel were part of the team. Unfortunately the author could not remember details but assumed that health professionals also included pharmacy personnel.

Assessment of heterogeneity

We used the I^2 statistic to measure heterogeneity among the trials. We planned to explore substantial heterogeneity (I^2 >50%) by pre-specified subgroup analysis. However, there was no evidence of serious heterogeneity in our review hence we did not perform sub-group analyses.

Assessment of reporting biases

We contacted study authors to provide any missing outcome data. However, only one author from one included trial responded but did not provide any additional information. If any meta-analysis in our review was to include 10 or more studies, we

For peer review only

planned to assess the potential for publication bias using a funnel plot [20 21]. We minimised the potential for publication bias by using a comprehensive search strategy, as described above.

Data synthesis

We conducted meta-analysis, using Cochrane's Review Manager software [22]. We used a random effects model considering the diverse settings of the included studies. Where meta-analysis was not possible, for instance, for adherence and cost, a narrative synthesis of the evidence was carried out. We summarised the quality (or certainty) of evidence for each outcome using GRADE [23 24]. The GRADE system defines the certainty of evidence for each outcome as "the extent of our confidence that the estimates of effect are correct" [20]. For GRADE we independently considered the five factors for downgrading the evidence to assess the certainty of a body of evidence as it relates to the studies that contribute data to the meta-analyses for the pre-specified outcomes. The quality rating across studies has four levels: high, moderate, low or very low. Randomised trials are considered to be of high quality but can be downgraded for any of the following five reasons: risk of bias; indirectness of evidence; unexplained heterogeneity or inconsistency of results; imprecision of results; and, high probability of publication bias. Similarly, observational studies are considered to be of low quality, but can be upgraded for any of these three reasons: large magnitude of effect; all plausible confounding would reduce a demonstrated effect; and, the presence of a dose-response gradient.

Subgroup analysis and sensitivity analysis

Due to the absence of significant heterogeneity, we did not perform subgroup analyses. Additionally, sensitivity analyses were not conducted to investigate the

For peer review only

effect of excluding studies with high or low risk of bias due to the small number of studies included in the review.

Results

A total of 3557 records were identified. Following title and abstract screening, eight studies were identified for full-text screening. The full-text review resulted in three studies [16 25 26] that met the review inclusion criteria. The study selection process is shown in Figure 1. The characteristics of included and excluded studies are shown in Tables 3 and 4 respectively. In total, 1993 HIV-infected patients on ART were enrolled in the three included studies. Of these, 1121 were assigned to non-pharmacy personnel model of ART delivery while 872 were assigned to pharmacy personnel model of ART delivery. However, 974 and 746 were available for analysis in the non-pharmacy personnel and pharmacy personnel groups respectively for all the three studies. Overall, there were no differences in loss to follow up between non-pharmacy and pharmacy personnel groups (3% versus 3%) p=0.9. Two studies [16], [25] included lay people in the non-pharmacy personnel group as shown in Table 3 while the third [26] included nurses in the non-pharmacy personnel group.

For peer review only

378 **Table 3: Summary of included studies**

Study ID	Setting	Methods	Outcomes
Selke 2010 [16]	Kenya	**Study design:** Cluster randomised trial<br><br>**Description of interventions:**<br>**Non-pharmacy personnel (home-based) group:**<br>Community Care Counsellors were:<br>• Clinically stable patients with self-reported 100% adherence to ART over the six-month period before recruitment.<br>• A patient considered by the clinic staff to be good role model and mentor for other patients.<br>Duties for care givers during home visits:<br>• Obtained and entered data concerning patients' symptoms.<br>• Vital sign assessment.<br>• Assessment of adherence to ART and opportunistic infections prophylaxis.<br>• Distributed a one-month supply of the patients' medications (from a prefilled kit).<br><br>**Pharmacy personnel (facility-based) group:**<br>• Nurses/clinical officers or physicians performed the following:<br>○ Took an interim medical history.<br>○ Addressed any acute concerns.<br>○ Reviewed medications.<br>○ Prescribed ART and opportunistic infection prophylaxis.<br>• Patients collected a one-month supply of their medication dispensed from the pharmacy.	• Adherence<br>• Viral load responses<br>• Intercurrent opportunistic infections<br>• Hospitalisation<br>• Loss to follow up<br>• Change in second-line therapy<br>• Mortality
Jaffar 2009 [25]	Uganda	**Study design:** Cluster-randomised trial	• Plasma RNA VL>500 copies per

For peer review only - http://bmjopen.bmj.com/site/about/guidelines.xhtml

For peer review only

		**Description of interventions:**<br>**Non-pharmacy personnel (home-based care) group:**<br>• Field workers with degree or college diploma who received a week of intensive training at start of study and yearly refresher courses on:<br>○ The principles of antiretroviral therapy.<br>○ Adherence support.<br>• Field workers visited patients in the homes on motor bikes every month to:<br>○ Deliver drugs.<br>○ Monitor participants with a checklist of signs and symptoms of drug toxicity and disease progression.<br>○ Provide adherence support.<br>○ Referred patients to physician or counsellor at the clinic when judged necessary.<br>• At two and six months after starting therapy, all patients were reviewed by a Medical Officer at the clinic and then at the twelfth month.<br>• Drugs were not dispensed during the clinic visits for this group.<br>• Patients were visited again when not found at home.<br>• Patients asked to come to clinic when unwell.<br><br>**Pharmacy personnel (facility-based) group:**<br>• Patients collected drugs every month from the pharmacy.<br>• Routine reviews by a Medical Officer and counsellor at two and three months after start of treatment & every three months thereafter.<br>• Patients assessed by a nurse and referred to a doctor when necessary during clinic visits.<br>• Patients followed up at home by field workers when missed an appointment.<br>• Patients received vouchers for their households for free voluntary counselling and testing at the clinic.	mL.<br>• Either plasma RNA >500 copies per mL if undetectable at six months, or an increase of 1000 copies between two consecutive tests if RNA detectable at six months.<br>• All-cause mortality.<br>• Mortality or plasma RNA VL >500 copies per mL.<br>• Admitted on one or more occasions.<br>• All admissions.<br>• Death, first admission, or change to second-line therapy.<br>• Frequency of out-patient attendance.<br>• Adherence.<br>• Costs of health service delivery and costs incurred by patients to access care.

For peer review only

For peer review only - http://bmjopen.bmj.com/site/about/guidelines.xhtml

		• Patients asked to come to clinic when unwell.	
Silveira 2014 [26]	Brazil	**Study design:** Individually randomised trial.<br><br>**Description of interventions:**<br>**Pharmacy personnel group:**<br>Patients received this intervention on a monthly basis through the Da´der method which consisted a series of scheduled meetings where the pharmacist and patient addressed, reviewed, and solved drug-related problems for a 12-month period. Patients received structured counselling from pharmacists on their prescription regimens at the time of their initial drug dispensing and at monthly refill visits.<br>Key elements included:<br>• Reviewing the prescription with the patient.<br>• Reviewing a card on which medications were colour-coded to facilitate recognition and reduce confusion that might arise from complicated drug names.<br>• Reviewing the schedule, length and date of next appointment.<br>• Reviewing patient's understanding of the prescription by asking patient to describe it and giving patients verbal information on the expected side effects of their medications and instructing them to seek medical assistance by calling the pharmacist if side effects occurred.<br><br>**Non-pharmacy personnel group:**<br>Patients received Usual Care (UC) for ART drug distribution. The control group received UC for ART drug delivery. This included the following:<br>At the first appointment, when patients received their medications, a nurse first provided information on:<br>• The regimen,<br>• How and when to use the medications,	• Self-reported adherence calculated by taking the number of tablets that patients reported ingesting and dividing it by the number they should have ingested. Patients were classified as adherent if they reported using 95% or more of the tablets prescribed<br>• Depression: This was investigated using a validated Portuguese-language version of BDI with standard scoring

For peer review only - http://bmjopen.bmj.com/site/about/guidelines.xhtml

		• Principal side effects.<br>• Importance of adhering to the prescription.<br><br>Afterward, patients picked up their medications at a drug-dispensation counter. They had no encounters with a pharmacist. Patients were informed that they could schedule time with a nurse for any questions about their treatment or disease.<br>In both groups, patients were scheduled every four months for medical appointments and measurements of viral load and CD4 count. Participants who did not appear for their regularly scheduled appointment were contacted by telephone and asked to return.	

For peer review only

Table 4: Summary of excluded studies

Study ID	Reasons for exclusion
March 2007 [27]	All participants were enrolled in the pharmacist-managed HIV drug optimisation clinic, with no non-pharmacist control group. We excluded this study because we could not make the comparisons due to the absence of a non-pharmacy personnel group.
Chang 2010 [28]	Non-pharmacy personnel did not dispense or distribute ART
Kiweewa 2013 [29]	Dispensing of ART done in same way for both groups hence we could not make comparisons.
Hansudewechaku 2012 [30]	Cohort study with comparisons between community and non-community hospitals with both groups having pharmacy personnel
Henderson 2011 [31]	Observational study with no comparison group.

Risk of bias in included studies

We judged the three trials to have a low risk of selection bias as a result of adequate randomisation and allocation concealment. The studies by Selke et al [16] and Jaffar et al [25] were judged to have low risk of performance bias for blinding because the reported outcomes were objective and unlikely to be influenced by lack of blinding. However, the Selke trial [16] did not give sufficient information to permit judgment of whether or not detection bias was present. The Silveira trial [26] was judged to have a high risk of performance and detection bias due to lack of blinding for participants, personnel and outcome assessment respectively for all study outcomes. The three studies were judged to have a low risk of attrition bias as they did not have differential or large numbers of losses to follow-up across the intervention arms. The risk of selective reporting bias was judged to be low for the study by Jaffar et al [25] and unclear for the Selke trial [16] because the trial protocol was not available. The Silveira

For peer review only

For peer review only - http://bmjopen.bmj.com/site/about/guidelines.xhtml

trial [26] was judged as having unclear risk of reporting bias due to insufficient information and as having a high risk of other biases due to inadequate sample size. Figure 2 provides a graphical summary of the risk of bias assessments.

Mortality

There was one reported death in the non-pharmacy personnel group in the Selke trial [16] during the study period. However, the authors reported the death as unrelated to HIV but rather due to use of herbal abortifacients. The trial by Jaffar [25] in Uganda did not find differences in mortality rates between the non-pharmacy and pharmacy personnel groups: adjusted rate ratio RR 0.95 (95% CI: 0.71 to 1.28). Five deaths were reported in the non-pharmacy personnel group compared to none in the pharmacy personnel group in the Silveira trial [26]. This difference was not statistically significant (RR 11.00, 95% CI: 0.61 to 197.35). When these data were pooled as shown in Figure 3, there was no difference in mortality between the groups cared for by non-pharmacy personnel and those cared for by pharmacy personnel (RR 1.86; 95% CI: 0.44 to 7.95), with no significant heterogeneity (Chi^2 = 3.34; df = 2; p = 0.19; I^2 = 40%). The certainty of the evidence for this outcome was low due to serious indirectness and imprecision as shown in Table 5. We downgraded for indirectness because two of the three trials (Selke [16] and Jaffar [25]) compared complex interventions that included, but were not limited to, pharmacy and non-pharmacy personnel as shown in Table 5. There were few deaths in the trials and the effect estimate was imprecise, with wide confidence intervals ranging from appreciable benefit to substantial harm as reported in Table 5.

For peer review only - http://bmjopen.bmj.com/site/about/guidelines.xhtml

Table 5: GRADE Summary of findings table for pharmacy versus non-pharmacy personnel for dispensing antiretroviral therapy

Population: People living with HIV
Setting: Brazil, Kenya, and Uganda (one study per country)
Intervention: Non-pharmacy personnel for dispensing antiretroviral therapy
Control: Pharmacy personnel for dispensing antiretroviral therapy

**Outcomes** (Mean follow-up: 12 months)	**Pharmacy personnel**	**Non-pharmacy personnel**	**Relative effect (95% CI)**	**No. of Participants (studies)**	**Quality of evidence (GRADE)**
**Mortality**	**76 per 1000**	**141 per 1000** (33 to 602)	**RR 1.86** (0.44 to 7.95)	1993 (three studies)	⊕⊕⊖⊖ **low**[1,2]
**Virological failure**	**150 per 1000**	**138 per 1000** (109 to 172)	**RR 0.92** (0.73 to 1.15)	1752 (three studies)	⊕⊕⊖⊖ **low**[1,2]
**Loss to follow up**	**28 per 1000**	**31 per 1000** (19 to 53)	**RR 1.13** (0.68 to 1.91)	1993 (three studies)	⊕⊕⊖⊖ **low**[1,2]

CI: Confidence interval; **RR:** Risk ratio;

GRADE Working Group grades of evidence
High certainty: Further research is very unlikely to change our confidence in the estimate of effect.
Moderate certainty: Further research is likely to have an important impact on our confidence in the estimate of effect and may change the estimate.
Low certainty: Further research is very likely to have an important impact on our confidence in the estimate of effect and is likely to change the estimate.
Very low certainty: We are very uncertain about the estimate.

[1] We downgraded by one for serious indirectness: Two trials compared complex interventions that included, but were not merely limited to, pharmacy and non-pharmacy personnel.
[2] We downgraded by one for serious imprecision: There are few events and the effect estimates have wide confidence intervals, ranging from appreciable benefit to harm.

For peer review only

Virological response

In the Selke trial [16], we did not find significant differences between groups in the proportions of undetectable viral load (10.5% in the intervention group versus 13.5% in the control group), p = 0.65 at 12 months.

Jaffar et al [25] investigated HIV-RNA suppression and survival over time by study group. They found that rates of detection of plasma viral loads of more than 500 copies per mL after six-month visit were similar in both groups (RR 1.04, 95% CI: 0.78 to 1.40). Similarly, the composite rates of either plasma RNA viral loads of more than 500 copies per mL after a six-month visit were comparable in those who had undetectable viral loads at six months, or an increase in plasma RNA viral load of 1000 copies per mL between two consecutive tests in those who had detectable viral loads at six months (RR: 0.99, 95% CI: 0.73 to 1.34). By the end of the study 66% of the participants in the non-pharmacy personnel group and 63% of the participants in the pharmacy personnel group had undetectable viral loads.

Silveira [26] in Brazil did not report significant differences in viral load at baseline. In the pharmacy personnel group 52.1% (n=86) and in the non-pharmacy personnel group 51.2% (n=85), p=0.86 were reported to have undetectable viral load. However, fifty participants (23 in the pharmacy personnel group and 27 in the non-pharmacy personnel group) were reported to have detectable viral load at baseline. For these, the authors conducted a sub-group analysis after two years of follow up. Seventy-six per cent (95% CI: 50.0 to 94.7) in the pharmacy personnel group and 42.9% (95% CI: 23.9 to 68.3) in the non-pharmacy personnel group cumulatively had virological success.

For peer review only - http://bmjopen.bmj.com/site/about/guidelines.xhtml

In a meta-analysis, we detected no differences in virological failure between the group cared for by non-pharmacy personnel and that cared for by pharmacy personnel (RR 0.92; 95% CI: 0.73 to 1.15), with no significant heterogeneity detected between the trials ($Chi^2 = 0.24$; df = 2; p=0.89; $I^2 = 0\%$) as shown in Figure 4. The quality of the evidence for this outcome was low due to serious indirectness and imprecision as shown in Table 5.

Loss to follow up

Selke [16] reported no differences in median duration (days) in the study for both groups at six or 12 months (343 days for both groups at 12 months), p=0.2. At study closure, the proportion lost to follow up was similar between the two groups (5.2% for the non-pharmacy personnel group and 4.5% for the pharmacy personnel group), p=1.0. Jaffar [25] reported very low rates of loss to follow up (1% in the non-pharmacy versus 2% in the pharmacy personnel group). However, 3% in the non-pharmacy personnel group and 6% in the pharmacy personnel group respectively withdrew from participation. Similarly, in the Silveira trial [26], 4.8% (n = 8) participants were lost to follow up in the non-pharmacy personnel group and 3% (n = 5) in the pharmacy personnel group. Overall, there were no differences in loss to follow up between the non-pharmacy personnel and the pharmacy personnel group with both having a 3% loss to follow up, p=0.96.

In our meta-analysis, we detected no difference in loss to follow up between the groups (RR 1.13; 95% CI: 0.68 to 1.91), with no significant heterogeneity ($Chi^2 = 0.54$; df = 2; p=0.76; $I^2 = 0\%$) as shown in Figure 4. The quality of the evidence for this outcome was low due to serious indirectness and imprecision as shown in Table 5.

For peer review only

For peer review only - http://bmjopen.bmj.com/site/about/guidelines.xhtml

Clinic visits and incidence of opportunistic infections

Selke [16] reported significantly more clinic visits in the pharmacy compared to the non-pharmacy personnel group (mean visits 12.6 versus 6.4), p<0.001. Despite fewer clinic visits for the non-pharmacy personnel group, clinical outcomes between the two groups were comparable with an incidence of 13.6 HIV opportunistic infections per 100 person-years in the non-pharmacy personnel group and 19.8 HIV opportunistic infections per 100 person-years in the pharmacy personnel group, p=0.42. Although the non-pharmacy personnel group was found to have fewer clinic visits, the authors observed that this group undertook 64% more clinic visits than originally scheduled. The study by Jaffar [25] found a high frequency of outpatient attendance (15,242 visits) in the pharmacy compared to the non-pharmacy personnel group (6,691 visits). However, they found similar distribution of new diagnoses between groups where more than 50% were infectious and parasitic infections. The trial by Silveira [26] did not report on clinic visits.

Adherence

Both groups in the Selke trial [16] demonstrated high levels of self-reported medication adherence with no statistical differences between groups at six and 12 months (96% versus 97%), p=0.71 and (94% versus 97%), p=0.47 respectively. In the Jaffar trial [25], self-reported adherence at routine clinical and counselling reviews was high with 94% in the intervention group and 91% in the control group. In the Silveira trial [26], participants' self-reported adherence remained the same between the two groups after controlling for potential confounders RR 1.05, 95% CI: 0.95-1.15, p=0.35.

Cost

In the trial by Jaffar [25], a societal perspective economical analysis showed a higher mean cost per patient per year in the pharmacy personnel group ($838) for health

For peer review only - http://bmjopen.bmj.com/site/about/guidelines.xhtml

services (staff, transport, drugs, laboratory and clinical services, sensitisation, training and workshops, utilities, supervision and overhead capital) compared to non-pharmacy personnel group ($793). Similarly, total per patient costs to access care per year were higher in the pharmacy personnel group ($54) compared to non-pharmacy personnel group ($18). Costs to access care included transport, lunch, childcare and lost work time. The outcome cost was not pre-specified in the protocol, however, this provides additional data that may be relevant for decision makers and was therefore included for consideration.

Acceptability to pharmacy personnel, non-pharmacy personnel and patients and harm, including error rates

These outcomes were not reported in the included trials.

Discussion

Summary of main results

Two cluster randomised clinical trials and one non-blinded randomised controlled trial were included in this review of effects of shifting responsibility from pharmacy to non-pharmacy personnel for adherence assessment and dispensing antiretroviral drugs to HIV-infected patients.

In our meta-analysis, we found low quality evidence that there may probably be no difference in mortality, virological response, and loss to follow up between non-pharmacy personnel and pharmacy personnel distributing ART to patients. In addition, we did not observe significant differences in adherence to treatment. Selke et al [16] reports that the non-pharmacy personnel group had significantly fewer all-cause sick visits to the clinic compared to the pharmacy personnel group. Although the main

For peer review only - http://bmjopen.bmj.com/site/about/guidelines.xhtml

difference between the Selke [16] and Jaffar [25] trials was the level of education of the non-pharmacy personnel, it did not seem to affect their overall performance [16 25]. Additionally, the Jaffar trial [25] found the non-pharmacy dispensing strategy to be cost–effective and cheaper to run than the pharmacy dispensing strategy by almost 45 US dollars per patient per annum and this accounted for only 6% of the total cost of healthcare service expenditure for the intervention group [25]. The patients who were accessing their medications and other healthcare services from pharmacy personnel incurred more costs in terms of transportation, lunch, childcare costs and lost work time. In the first year of the study, the pharmacy personnel group incurred double the cost of healthcare services per patient per annum compared to the non-pharmacy personnel group. This is a challenge in most poor settings in Africa where many cannot afford basic necessities and this further impedes their access to treatment [25]. Lack of or inadequate financial resources are major factors in late presentation to health facilities, poor access to HIV care and support, and low retention in care after initiation of ART [32 33]. Although health services are provided for free in most public institutions, the cost of accessing the services may serve as a hindrance to achieving optimal care and support for HIV-infected patients [32]. The non-pharmacy personnel dispensing ART could therefore considerably reduce the cost of services for both patients and governments with potentially favourable clinical outcomes.

Although issues related to drug-drug interaction, medication errors, pre-screening for ART and some special cases of adherence to therapy may be beyond the capacity of non-pharmacy personnel, targeted pharmacy support to non-pharmacy personnel could offer benefits to patients having challenges with their medications especially those who are taking concomitant medications with ART [34].

For peer review only - http://bmjopen.bmj.com/site/about/guidelines.xhtml

This review and meta-analysis has shown that the use of non-pharmacy personnel comprising either lay people or other health professionals such as nurses who are given well-tailored and comprehensive short trainings with refreshers could effectively support ART dispensing systems for HIV-infected patients. The use of non-pharmacy personnel such as lay people who are known and trusted members of the community in dispensing ART and monitoring patients' health would enable identification of vital psychosocial features that might be overlooked by physicians, nurses and pharmacists at the facility. Some of these psychosocial problems such as gender-based violence, food insecurity and alcohol abuse negatively impact on adherence and retention in care [16]. In the case of community-based non-pharmacy personnel, they could also serve as a link between the patients and other healthcare workers and facilitate smooth communication between the patients and the pharmacists on issues such as changes in regimen and dosage of drugs [16 34].

Overall completeness and applicability of evidence

Although it may not be possible to rule out publication and language biases in systematic reviews and that this could potentially threaten the conduct of this review, our trial search was not restricted to any language, setting or publication status. We are of the opinion that we identified all the existing randomised controlled trials relevant to our research question. However, there is a possibility that some unpublished or published trials were not accessible by our search.

We identified three studies for inclusion in this review. The trials generally had small sample sizes. Recruitment restrictions such as basing recruitment on location of residence of the participants resulted in a small sample size in the Selke trial [16]. The

For peer review only - http://bmjopen.bmj.com/site/about/guidelines.xhtml

trials did not include some of the outcomes of interest for decision-making such as acceptability to participants and feasibility of the intervention, for example, adherence to treatment. These limitations might have affected our conclusions about the findings from these trials.

Certainty of evidence

We assessed the certainty or quality of evidence using the GRADE approach [35]. . Evidence from this review should be applied with caution considering its low quality. Three outcomes in this review were found to be of low quality evidence. We are therefore not confident enough to state that the estimates lie close to the true value and it is possible that different and further studies are needed to contribute to the body of knowledge to increase our certainty in these review findings.

Conclusions

There is low-quality evidence that the use of non-pharmacy personnel including lay people in dispensing and distributing ART may be as effective as pharmacy personnel performing these functions in resource-limited settings when accompanied by continued capacity building and supervision by trained pharmacy personnel. The introduction of non-pharmacy personnel is probably cost-effective for the patient and health system. Strong referral systems are crucial in task-shifting of dispensing responsibilities to non-pharmacy personnel to support patients that require advanced medical attention. Due to the critical shortage of human personnel in the health sector, most HIV programmes engaged other cadres of health personnel other than doctors to prescribe and dispense ART at the onset of their programme implementation. This might have contributed to scarcity of studies on the use of pharmacy personnel for dispensing and distributing ART. More trials, with adequate sample sizes to detect

For peer review only

clinically relevant health benefits are therefore needed to investigate task-shifting of dispensing and distributing ART from pharmacy to non-pharmacy personnel.

Author contributions

EN, TK, and CSW conceived the idea; NMM, TK and CSW drafted the review protocol; NMM, OA, TK and CSW conducted screening, selected the studies, extracted data, performed analyses and drafted the manuscript. All authors reviewed and approved the manuscript.

Acknowledgements

Funding for preparing this review was provided by the Department of HIV/AIDS at the WHO Headquarters in Geneva, Switzerland through a grant from University of California, San Francisco. The authors would like to thank George Rutherford and Hacsi Horvath (Global Health Sciences, University of California, San Francisco, USA) for support and specific assistance in developing the search strategy for electronic databases. Neither the authors' institutions nor the funders played a role in preparing the manuscript, and the views expressed therein are solely those of the authors. We would like to thank Joy Oliver from Cochrane South Africa, South African Medical Research Council for assistance with the updated search. Both Charles Wiysonge and Tamara Kredo are partly supported by the Effective Health Care Research Consortium. This Consortium is funded by the UK government for the benefit of developing countries (Grant: 5242). The views expressed in this publication do not necessarily reflect UK government policy.

For peer review only

For peer review only - http://bmjopen.bmj.com/site/about/guidelines.xhtml

Competing interests

None declared

Data sharing Statement

Additional unpublished data is not available

References

1. UNAIDS. How AIDS changed everything - MDG 6: 15 years, 15 lessions of hope from the AIDS response. http://wwwunaidsorg/en/resources/presscentre/pressreleaseandstatementarchive/2015/july/20150714 PR MDG6report 2015;**accessed 22nd September 2015**.

2. Palella FJ DK, Moorman AC, Loveless MO, et al. . Declining morbidity and mortality among patients with advanced human immunodeficiency virus infection. New England Journal of Medicine 1998;**338**(13):853-60.

3. Anglemyer A RG, Horvath T, Baggaley RC, Egger M, Siegfried N. . Antiretroviral therapy for prevention of HIV transmission in HIV-discordant couples. Cochrane Database Syst Rev 2013;**4**(CD009153).

4. Ford N, Calmy, A., Mills, E,J. The first decade of antiretroviral therapy in Africa. Globalization and Health 2011;**7**(33).

5. King RC, Fomundam, H.N. Remodeling pharmaceutical care in sub-Saharan Africa (SSA) amidst human resources challenges and the HIV/AIDS pandemic. Int J Health Plann Manage 2010;**25**(1):30-48.

6..SAPC. Pharmacy Human Resources data. http://wwwhealthlingorgza/healthstats/283/data;accessed **15th August, 2015**.

7. Tseng A, Foisy, M., Hughes, C.A., et, al.,. Role of pharmacists in caring for patients with HIV/AIDS: Clinical Practice Guidelines. CJHP 2012;**65**(2):125-45.

8. Saberi P, Dong, B.J., Johnson, M.O., Greenblatt, R.M., Cocohoba, J.M. The impact of HIV clinical pharmacists on HIV treatment outcomes: systematic review. Patient Prefer Adherence 2012;**6**:297-322.

9. Kitahata MM, Koepsell, T.D., Deyo, R.A., et, al. Physicians experience with the acquired immunodeficiency syndrome as a factor in patients' survival. N Engl J Med 1996;**334**(11):701-6.

For peer review only

For peer review only - http://bmjopen.bmj.com/site/about/guidelines.xhtml

10. Landon BE, Wilson, I..B., Malness, K., et, al. Physician specialization and the quality of care for human immunodeficiency virus infection. Arch Intern Med 2005;**165**(10):1133-9.
11. Kredo T AF, Bateganya M, Pienaar ED. . Task shifting from doctors to non-doctors for initiation and maintenance of antiretroviral therapy. Cochrane Database of Systematic Reviews 2014;**CD007331**(7).
12. MSH. *Managing Drug Supply*. Second Edition ed. 14 Oakwood Avenue, West Hartford, Connecticut, 06119-227 USA: Kumarian Press Inc, 1997.
13. WHO. Management of Drugs at health Centre Level - Training Manual. WHO Regional Office for Africa 2004;http://www.who.int/medicinedocs/en/d/js791e.
14. Decroo T, Telfer, B., Biot M, et al,. . Distribution of antiretroviral treatment through self-forming groups of patients in Tete province, Mozambique. J Acquir Immune Defic Syndr 2011;**56**:39-44.
15. Kipp W, KondeLule, J., Rubaale,T., et al. . Comparing antiretroviral treatment outcomes between a prospective community-based and hospital-based cohort of HIV patients in rural Uganda. BMC Int Health Hum Rights 2011;**11Suppl**(2):S12.
16. Selke H, Kimaiyo, S., Sidle, J.E., et, al., . Task-shiftingofantiretroviraldelivery from health care workers to persons living with HIV/AIDS: clinical outcomes of a community-based program in Kenya. J Acquir Immune Defic Syndr 2010;**55: 483-90.**
17. Callaghan M, Ford, N., Schnider, H. Asystematic review of task-shifting for HIV treatment and care in Africa. Human Resources for health 2010;**8**(8).
18. Laurant M RD, Hermens R, Braspenning J, Grol R, Sibbald B. . Substitution of doctors by nurses in primary care. Cochrane Database of Systematic Reviews 2005;**CD001271**.
19. Mbeye NM, Kredo, T., Wiysonge, C.S. The effects of shifting tasks from pharmacy to non-pharmacy personnel for providing antiretroviral therapy to people living with HIV: a systematic review protocol. BMJ Open 2016;**6:e008195**.
20. Higgins JPT, Green, S. (eds). . Cochrane Handbook for Systematic Reviews of Interventions. Wiley-Blackwell 2008.
21. Egger M DSG, Schneider M, Minder C. . Bias in meta-analysis detected by a simple, graphical test. BMJ 1997;**315**(7109):629-34.
22. (RevMan) RM. Computer program. The Nordic Cochrane Centre, The Cochrane Collaboration, Copenhagen 2015;**Version 5.3**.
23. Guyatt GH OA, Santesso N, Helfand M, Vist G, Kunz R, et al. . GRADE guidelines: 12. Preparing Summary of Findings tables-binary outcomes. Journal of Clinical Epidemiology 2013;**66**(2):158-72.
24. Guyatt GH TK, Oxman AD, Walter SD, Patrick D, Furukawa TA, et al. . GRADE guidelines: 13. Preparing Summary of Findings tables and evidence profiles-continuous outcomes. Journal of Clinical Epidemiology 2013;**66**(2):173-83.
25. Jaffar S, Amuron B., Foster S., et, al., . Rates of virologic failure in patients treated in a home-based versus a facility-based HIV-care model in Jinja, southeast Uganda: a cluster-randomised equivalence trial. Lancet 2009;**DOI:10.1016/S1040-6736(09)61674-3.**

For peer review only

For peer review only - http://bmjopen.bmj.com/site/about/guidelines.xhtml

26. Silveira MPT, Guttier, M.C., Page, K., Moreira, L.B. Randomized Controlled Trial to Evaluate the Impact of Pharmaceutical Care on Therapeutic Success in HIV-Infected Patients in Southern Brazil. AIDS Behav 2014;**18**:S75-S84.

27. March K MM, Louie SG. . Effects of pharmacists' interventions on patient outcomes in an HIV primary care clinic. American Journal of Health-System Pharmacy 2007;**64**:2574-78.

28. Chang LW KJ, Nakigozi G, Ssempijja V, Packer AH, Serwadda D, Quinn TC, Gray RH, Bollinger RC, Reynolds SJ. . Effect of peer health workers on AIDS care in Rakai, Uganda: A cluster-randomized trial. PLoS ONE 2010:; **5** (6).

29. Kiweewa FM WD, Nakibuuka J, Mubiru M, Bagenda D, Musoke P, Fowler MG, Antelman G. . Noninferiority of a task-shifting hiv care and treatment model using peer counselors and nurses among ugandan women initiated on ART: Evidence from a randomized trial. Journal of Acquired Immune Deficiency Syndromes 2013;**63** (4):e125-e32.

30. Hansudewechakul R NT, Katana A, Faikratok W, Lolekha R, Thainuea V, McConnell MS. . Successful clinical outcomes following decentralization of tertiary paediatric HIV care to a community-based paediatric antiretroviral treatment network, Chiangrai, Thailand. Journal of the International AIDS Society 2012;**15 (2)**

31. Henderson KC HJ, Johnson SC, Valuck RJ, Kiser JJ. . Assessing the effectiveness of pharmacy-based adherence interventions on antiretroviral adherence in persons with HIV. IDS Patient Care and STDs **25**(4):221-28.

32. Hardon A, Akurut, D., Comoro, C., et al.,. Hunger, waiting time and transport costs: time to confront challenges to ART adherence in Africa. AIDS Care 2007;**19: 658–65.**

33. Rosen S, Fox, MP., Gill, C.J. . Patient retention in antiretroviral therapy programs in sub-Saharan Africa: a systematic review. PLoS Med 2007;**4: e298.**

34. Seden K, . Bradley, M., Miller, AR., Beadsworth, M.B., Khoo, S.H. . The clinical utility of HIV outpatient pharmacist prescreening to reduce medication error and assess adherence. Int J STD AIDS 2013:;**24 (3);237-41.**

35. Guyatt G.A, Akl, E.A., Kunz, R., Vist, G., Brozek, J., Norris, S,. Falck-Ytter, Y., Glasziou, P., DeBeer, H., Jaeschke, R,. Rind, D., Meerpohl, J., Dahm, P., Schünemann, H.J. . GRADE guidelines: 1. Introduction-GRADE evidence profiles and summary of findings tables. Journal of Clinical Epidemiology 2011;**64(4): 383-94**.

List of figures

Fig 1: Flow diagram showing the search and selection of studies

Fig 2: Summary of risk of bias in included studies

Fig 3: Effect of shifting dispensing of ART from pharmacy to non-pharmacy personnel on mortality

For peer review only

744 **Fig 4: Effect of shifting dispensing of ART from pharmacy to non-pharmacy**
745 **personnel on virological failure**

746

747 **Fig 5: Effect of shifting dispensing of ART from pharmacy to non-pharmacy**
748 **personnel on loss to follow up**

749

750 Supporting Information

751 S1. Task-shifting review protocol

752 S2. PRISMA Checklist

For peer review only

For peer review only - http://bmjopen.bmj.com/site/about/guidelines.xhtml

Fig 1

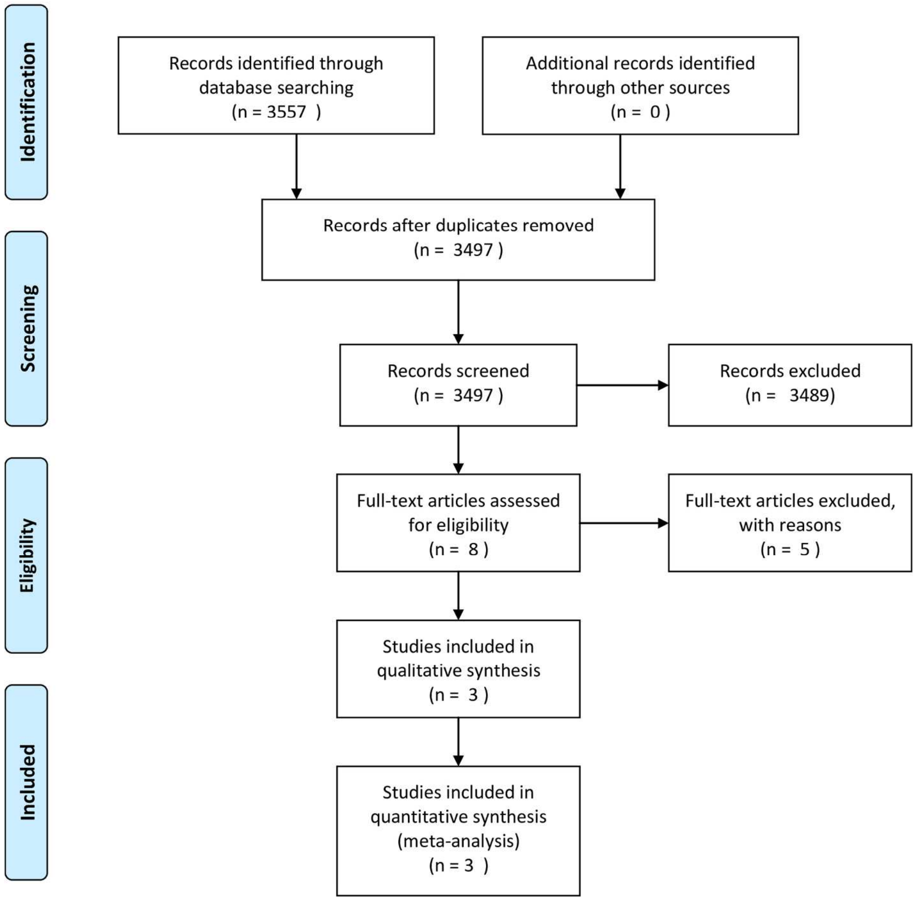

Fig 1: Flow diagram showing the search and selection of studies

168x222mm (300 x 300 DPI)

Fig 2.

Domain	Trial		
	**Jaffar 2009**	**Selke 2010**	**Silveira 2013**
Random sequence generation (selection bias)	+	+	+
Allocation concealment (selection bias)	+	+	?
Blinding of participants and personnel (performance bias)	+	+	−
Blinding of outcome assessment (detection bias)	+	?	−
Incomplete outcome data (attrition bias)	+	+	+
Selective reporting (reporting bias)	+	?	?
Other bias	+	?	−

\+ Low risk; − High risk; ? Unclear risk.

Fig 2: Summary of risk of bias in included studies

144x94mm (300 x 300 DPI)

view only

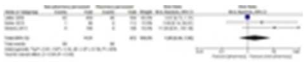

Effect of shifting dispensing of ART from pharmacy to non-pharmacy personnel on mortality

12x2mm (300 x 300 DPI)

For peer review only

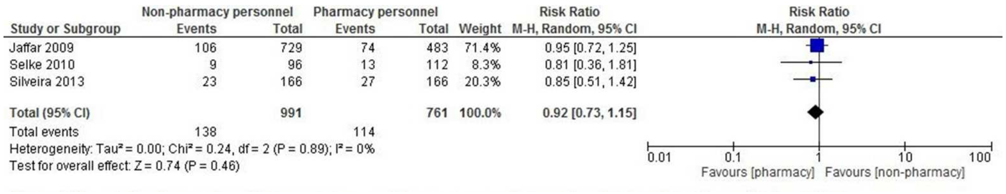

Effect of shifting dispensing of ART from pharmacy to non-pharmacy personnel on virological failure

79x14mm (300 x 300 DPI)

For peer review only

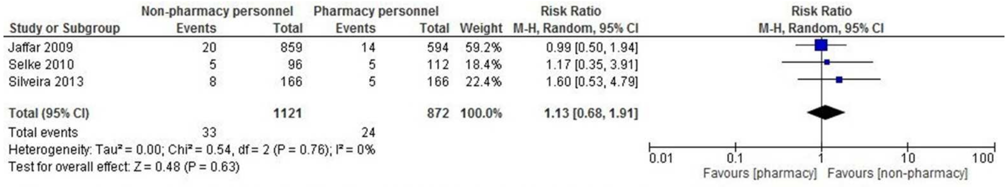

Effect of shifting dispensing of ART from pharmacy to non-pharmacy personnel on loss to follow up

79x15mm (300 x 300 DPI)

or peer review only

S2. PRISMA 2009 Checklist

Section/topic	#	Checklist item	Reported on page #
**TITLE**			
Title	1	Identify the report as a systematic review, meta-analysis, or both.	1
**ABSTRACT**			
Structured summary	2	Provide a structured summary including, as applicable: background; objectives; data sources; study eligibility criteria, participants, and interventions; study appraisal and synthesis methods; results; limitations; conclusions and implications of key findings; systematic review registration number.	2-3
**INTRODUCTION**			
Rationale	3	Describe the rationale for the review in the context of what is already known.	7
Objectives	4	Provide an explicit statement of questions being addressed with reference to participants, interventions, comparisons, outcomes, and study design (PICOS).	8-9
**METHODS**			
Protocol and registration	5	Indicate if a review protocol exists, if and where it can be accessed (e.g., Web address), and, if available, provide registration information including registration number.	8
Eligibility criteria	6	Specify study characteristics (e.g., PICOS, length of follow-up) and report characteristics (e.g., years considered, language, publication status) used as criteria for eligibility, giving rationale.	8-9
Information sources	7	Describe all information sources (e.g., databases with dates of coverage, contact with study authors to identify additional studies) in the search and date last searched.	9
Search	8	Present full electronic search strategy for at least one database, including any limits used, such that it could be repeated.	10-11
Study selection	9	State the process for selecting studies (i.e., screening, eligibility, included in systematic review, and, if applicable, included in the meta-analysis).	12
Data collection process	10	Describe method of data extraction from reports (e.g., piloted forms, independently, in duplicate) and any processes for obtaining and confirming data from investigators.	12-13
Data items	11	List and define all variables for which data were sought (e.g., PICOS, funding sources) and any assumptions and simplifications made.	12-13
Risk of bias in individual studies	12	Describe methods used for assessing risk of bias of individual studies (including specification of whether this was done at the study or outcome level), and how this information is to be used in any data synthesis.	13-16
Summary measures	13	State the principal summary measures (e.g., risk ratio, difference in means).	16
Synthesis of results	14	Describe the methods of handling data and combining results of studies, if done, including measures of consistency (e.g., I^2) for each meta-analysis.	16-17

For peer review only - http://bmjopen.bmj.com/site/about/guidelines.xhtml

S2. PRISMA 2009 Checklist

Section/topic	#	Checklist item	Reported on page #
Risk of bias across studies	15	Specify any assessment of risk of bias that may affect the cumulative evidence (e.g., publication bias, selective reporting within studies).	N/A
Additional analyses	16	Describe methods of additional analyses (e.g., sensitivity or subgroup analyses, meta-regression), if done, indicating which were pre-specified.	17-18
**RESULTS**			
Study selection	17	Give numbers of studies screened, assessed for eligibility, and included in the review, with reasons for exclusions at each stage, ideally with a flow diagram.	18
Study characteristics	18	For each study, present characteristics for which data were extracted (e.g., study size, PICOS, follow-up period) and provide the citations.	19-23
Risk of bias within studies	19	Present data on risk of bias of each study and, if available, any outcome level assessment (see item 12).	23-24
Results of individual studies	20	For all outcomes considered (benefits or harms), present, for each study: (a) simple summary data for each intervention group (b) effect estimates and confidence intervals, ideally with a forest plot.	24-29
Synthesis of results	21	Present results of each meta-analysis done, including confidence intervals and measures of consistency.	24-29
Risk of bias across studies	22	Present results of any assessment of risk of bias across studies (see Item 15).	23-24
Additional analysis	23	Give results of additional analyses, if done (e.g., sensitivity or subgroup analyses, meta-regression [see Item 16]).	n/a
**DISCUSSION**			
Summary of evidence	24	Summarize the main findings including the strength of evidence for each main outcome; consider their relevance to key groups (e.g., healthcare providers, users, and policy makers).	29-31
Limitations	25	Discuss limitations at study and outcome level (e.g., risk of bias), and at review-level (e.g., incomplete retrieval of identified research, reporting bias).	31-32
Conclusions	26	Provide a general interpretation of the results in the context of other evidence, and implications for future research.	31-32
**FUNDING**			
Funding	27	Describe sources of funding for the systematic review and other support (e.g., supply of data); role of funders for the systematic review.	33

For peer review only - http://bmjopen.bmj.com/site/about/guidelines.xhtml

Downloaded from http://bmjopen.bmj.com/ on September 16, 2016 - Published by group.bmj.com

Open Access Protocol

BMJ Open

The effects of shifting tasks from pharmacy to non-pharmacy personnel for providing antiretroviral therapy to people living with HIV: a systematic review protocol

Nyanyiwe M Mbeye,[1,2] Tamara Kredo,[1] Charles S Wiysonge[1,2]

For peer review only

To cite: Mbeye NM, Kredo T, Wiysonge CS. The effects of shifting tasks from pharmacy to non-pharmacy personnel for providing antiretroviral therapy to people living with HIV: a systematic review protocol. *BMJ Open* 2016;**6**: e008195. doi:10.1136/ bmjopen-2015-008195

▸ Prepublication history for this paper is available online. To view these files please visit the journal online (http://dx.doi.org/10.1136/ bmjopen-2015-008195).

Received 15 March 2015
Revised 16 November 2015
Accepted 17 November 2015

CrossMark

[1]Cochrane South Africa, South African Medical Research Council, Tygerberg, South Africa
[2]Faculty of Medicine and Health Sciences, Centre for Evidence-based Health Care, Stellenbosch University, Cape Town, South Africa

Correspondence to
Dr Nyanyiwe M Mbeye; nyanyiwembeye@gmail.com

ABSTRACT

Introduction: Shifting selected antiretroviral therapy (ART) tasks from specialised healthcare workers to those with shorter or less formal training has been implemented in resource-limited settings to alleviate critical shortages of human resources for health. However, the specifics of shifting ART dispensing from pharmacy to non-pharmacy personnel have not been addressed in a systematic review, although this can potentially increase access to ART. We will assess the effects of shifting dispensing and distribution of ART and adherence assessment from pharmacy to non-pharmacy personnel in low and middle-income countries.

Methods and analysis: We will search PubMed, CENTRAL, EMBASE, WHO Global Health Library and relevant grey literature for eligible controlled trials. Two authors will screen the search output, select eligible studies, assess risk of bias and extract data from included studies, resolving discrepancies by discussion and consensus. We will perform meta-analysis using both fixed and random effects models, investigate clinical and statistical heterogeneity, and assess our confidence in the overall evidence using standard Cochrane methods, including GRADE.

Ethics and dissemination: Only secondary data will be included in this review and ethics approval is not required. We will disseminate the review findings in various scientific fora, including peer-reviewed journals. The findings may help to inform policy makers in defining the scope of work of healthcare workers, and global recommendations for shifting the dispensing and distribution of ART from pharmacy to non-pharmacy personnel.

Trial registration number: CRD42015017034.

INTRODUCTION

Description of the condition

By March 2015, 15 million (40.7%) of the estimated 36.9 million people living with HIV (PLHIV) globally were receiving antiretroviral therapy (ART).[1] Combination ART is effective for reducing HIV related morbidity and mortality as well as preventing HIV transmission.[2] Initiating ART early in the course of HIV infection has been associated with better health outcomes, both at patient and population levels.[3 4] Scale-up of ART in low and middle income countries has averted more than 5 million deaths; however, bottlenecks preventing universal access to ART still exist. One challenge is the critical shortage of human resources for health (HRH), including for the delivery of essential HIV related pharmacy services.

The WHO recommends a minimum of one pharmacist per 2300 population, but most countries in low-resource settings such as sub-Saharan Africa have not yet met this target.[5] In addition to the absolute shortage, it is likely that there is an uneven distribution of pharmacists in such settings, as is the case with other specialist healthcare workers who

Strengths and limitations of this study

- To our knowledge, this is the first published protocol of a systematic review that will investigate the effects of task shifting from pharmacy to non-pharmacy personnel for dispensing or distributing antiretroviral therapy to patients living with HIV.
- The protocol was written according to the PRISMA-P (Preferred Reporting Items for Systematic Review and Meta-Analysis Protocols) recommendations.
- The review findings may help to inform antiretroviral therapy guidelines by the WHO.
- The possible weakness of the planned review would be the limitations of included studies, for example, high risk of bias and heterogeneity of settings, designs and effects.

For peer review only - http://bmjopen.bmj.com/site/about/guidelines.xhtml

Downloaded from http://bmjopen.bmj.com/ on September 16, 2016 - Published by group.bmj.com

tend to concentrate in urban areas and the private sector, further aggravating the HRH shortage.[6] For instance in South Africa, which is home to the largest number of PLHIV in any country in the world, in 2010 only 24% of registered pharmacists worked in the public sector where 80% of the population received care.[7]

Description of the intervention

Studies and programme reports indicate that involvement of pharmacy personnel in HIV care results in improved patient outcomes. For instance, in the USA, the use of a multidisciplinary team approach with pharmacists assuming a central role in ART initiation, dispensing and adherence counselling improved treatment outcomes such as viral load, patient retention and medication adherence.[8]

The work of pharmacists includes supply management, dispensing and distributing medications, promoting adherence, identifying and preventing potential medication-related issues, and monitoring and reporting adverse events. In some settings, programmes have implemented alternative models of pharmacy services that shift selected tasks from pharmacy to non-pharmacy personnel. Such alternative models could potentially increase the number of health workers involved in ART distribution, adherence counselling and patient education, free more time for pharmacy personnel, support the integration of ART in primary care settings, minimise the number of facility visits for ART collection, and reduce pharmacy queue waiting times for patients.[9]

However, the specifics of shifting ART related tasks from pharmacy to non-pharmacy personnel have not yet been addressed in a systematic review. We therefore plan to synthesise the evidence for task shifting in pharmacy services, where non-pharmacy personnel undertake ART dispensing and distribution and medication adherence counselling. For this systematic review, pharmacy personnel will include both pharmacists and pharmacy technicians. Pharmacy technicians constitute an important part of the pharmacy workforce in low and middle-income countries: a survey of 26 low and middle-income countries in 2011 revealed that pharmacy technicians constitute 10% (Nigeria) to 70% (Pakistan) of the pharmacy workforce.[10]

How the intervention might work

Within the last decade, several high HIV burden countries adopted task shifting strategies where nurses and non-physician clinicians initiate and maintain ART.[11] Although this has undeniably expanded access to ART, it is also increasingly essential that long facility waiting times and frequent facility visits to collect ART are addressed to alleviate the burden of care, both for patients and healthcare providers.[12 13]

Recent studies in Uganda, Kenya and Mozambique have shown positive outcomes when non-health professionals (lay people) delivered ART at the community level.[14] In Mozambique the use of PLHIV for distributing ART, monitoring adherence, reporting outcomes and referring sick patients to health facilities yielded a retention rate of 97.5% among stable patients on ART.[14] In a cluster randomised trial in Uganda, the use of trained community health workers produced comparable results with facility-based ART programme in terms of patient retention, viral load suppression and mortality rate.[15] Similar findings were also obtained in Kenya and Uganda when lay providers were engaged in ART delivery.[15 16]

Task shifting has therefore been seen as an achievable solution to the critical human resource shortages for scale-up of ART.[17] While it is imperative to increase the rate of recruitment and training of health workers as well as improve working conditions to reduce attrition and emigration, the HIV pandemic requires a more urgent measure to address the critical skills shortage.[18] Such measures may include shifting selected tasks (including dispensing and distributing ART and adherence counselling) from pharmacy to non-pharmacy personnel. The task shifting could free time for pharmacy personnel to focus on more technical functions such as supply management and pharmacovigilance.

Why this review is important

Previous systematic reviews of task shifting for increasing ART access focused on clinical services where nurses and non-clinician physicians provide care.[11] Dependence on and shortages of pharmacists are also key constraints on ART expansion, but the specifics of task shifting for ART dispensing or distribution from pharmacy to non-pharmacy personnel have not been reviewed systematically. We will systematically review the scientific literature and assess the efficacy and safety of task shifting models that use non-pharmacy personnel in dispensing or distributing ART and assessing adherence to treatment of HIV infection.

OBJECTIVE

The aim of this review is to evaluate the efficacy and safety of shifting dispensing and distribution of ART as well as assessment of adherence from pharmacy to non-pharmacy personnel.

METHODS

This review protocol has been registered in the PROSPERO International Prospective Register of Systematic Reviews (http://www.crd.york.ac.uk/PROSPERO), registration number CRD42015017034.

Criteria for considering studies for this review

Types of studies

We will include randomised controlled trials (RCTs) and non-RCTs, irrespective of whether allocation to interventions occurred at the individual or cluster level.

Types of participants

Participants will be PLHIV receiving ART.

For peer review only - http://bmjopen.bmj.com/site/about/guidelines.xhtml

Downloaded from http://bmjopen.bmj.com/ on September 16, 2016 - Published by group.bmj.com

Types of interventions

We will include studies that evaluate the shifting of selected tasks from pharmacy personnel to non-pharmacy personnel. The selected tasks include dispensing and distribution of ART and adherence assessment. Pharmacy personnel will include both pharmacists and pharmacy technicians. Non-pharmacy personnel may include (but are not limited to) nurses, non-physician clinicians, and lay providers such as patient peer groups, community volunteers, PLHIV and community health committees.

Types of outcome measures

Primary outcomes

The primary outcome for this review is risk of death.

Secondary outcomes

Our secondary outcome measures include:

- Virological suppression
- Number of all-cause sick visits made to the health facility, including for adverse events
- Loss to follow-up
- Adherence to ART (as measured within the study, eg, pill counts, recall methods, digital methods)
- Acceptability to pharmacy personnel, non-pharmacy personnel and patients
- Harm, including error rates.

Search methods for identification of studies

We will perform a comprehensive and exhaustive search of electronic databases and conference proceedings in an attempt to identify all relevant studies available by the search date, regardless of language of publication or publication status (published, unpublished, in press or in progress).

Databases of peer-reviewed literature

We will search the following electronic databases, from 1 January 1996 to the search date:

- Cochrane Central Register of Controlled Trials (CENTRAL)
- Excerpta Medica Database (EMBASE)
- PubMed
- ISI Web of Science (Science Citation index)
- WHO Global Health Library, which includes references from AIM (AFRO), LILACS (AMRO/PAHO), IMEMR (EMRO), IMSEAR (SEARO) and WPRIM (WPRO).

Along with appropriate Medical Subject Heading (MeSH) terms and relevant keywords, we will use the Cochrane Highly Sensitive Search Strategy for identifying reports of randomised controlled trials in MEDLINE,[19] and the Cochrane validated strategies for identifying references relevant to HIV infection and AIDS. To identify other study designs, the RCT string will be omitted. The search strategy will be iterative in that references of included studies will be searched for additional references. See table 1 for our provisional search strategy for electronic databases.

Conference databases

We will search conference abstract archives on the web sites of the Conference on Retroviruses and Opportunistic Infections (CROI), the International AIDS Conference (IAC) and the International AIDS Society Conference on HIV Pathogenesis, Treatment and Prevention (IAS), for all available abstracts presented at these conferences from 1996 to the search date.

Searching other resources

We will also search the references of relevant articles as well as the WHO International Clinical Trials Registry Platform (ICTRP) and Clinicaltrials.gov. We will contact relevant experts or organisations who may be aware of additional studies in this field.

Data collection and analysis

We will base the methodology for data collection and analysis on the guidance provided in the Cochrane Handbook of Systematic Reviews of Interventions.[19]

Selection of studies for inclusion

Two authors will read and assess the abstracts of identified publications for potentially eligible studies. We will obtain full text articles for all abstracts judged by at least one of the two authors, to be potentially eligible. Two authors will independently inspect these potentially eligible publications to establish the relevance of the article to the review according to the pre-specified criteria regarding study design, participants, interventions and outcome measures.

Data extraction and management

Two authors will independently extract data into a prepiloted data extraction form. The following characteristics will be extracted from each included study:

Study details: Complete citations of publications associated with the study, start and end dates, location, study design characteristics, type of facility involved, investigators, funding sources, recruitment, method of randomisation, sequence generation, method of allocation concealment, blinding of participants and personnel, blinding of outcome assessment, length of follow-up, losses to follow-up, withdrawals or drop-outs and other relevant details.

Details of the intervention: training of the cadre of health workers who were dispensing or distributing ART, what training or other support or supervision they received and other relevant details.

Details of participants: Trial inclusion and exclusion criteria, numbers of participants entering the trial, sex, clinical staging, CD4 count and other pertinent details.

Outcome details: Definitions of outcomes, details of how outcomes were assessed, numerators and denominators associated with each outcome, completeness of outcome data, effect estimates reported and other relevant outcome information.

For peer review only

For peer review only - http://bmjopen.bmj.com/site/about/guidelines.xhtml

Downloaded from http://bmjopen.bmj.com/ on September 16, 2016 - Published by group.bmj.com

Table 1 Proposed search strategy for electronic databases

ID	Search terms
PubMed	
#1	(task*[tiab] OR task-shifting[tiab] OR referr*[tiab] OR referral and consultation[mh] OR role*[tiab]) AND (health personnel [mh] OR doctor[tiab] OR doctors[tiab] OR clinician[tiab] OR clinicians[tiab] OR physician[tiab] OR physicians[tiab] OR “healthcare provider"[tiab] OR “healthcare providers"[tiab] OR “health care provider"[tiab] OR “health care providers"[tiab] OR pharmac*[tiab] OR apothecar*[tiab] OR chemist*[tiab] OR dispensar*[tiab])
#2	randomized controlled trial[pt] OR controlled clinical trial[pt] OR randomized controlled trials[MeSH] OR random allocation[MeSH] OR double-blind method[MeSH] OR single-blind method[MeSH] OR clinical trial[pt] OR clinical trials [MeSH] OR (“clinical trial"[tw]) OR ((singl*[tw] OR doubl*[tw] OR trebl*[tw] OR tripl*[tw]) AND (mask*[tw] OR blind*[tw])) OR random*[tw] OR research design[mh:noexp] OR prospective studies[MeSH] OR control*[tw] OR volunteer*[tw]) OR observational[tw] OR non-random*[tw] OR nonrandom*[tw] OR before after study[tw] OR time series[tw] OR cohort*[tw] OR cross-section*[tw] OR prospective*[tw] OR retrospective*[tw] OR research design[mh:noexp] OR follow-up studies [MeSH] OR longitud*[tw] OR evaluat*[tiab] OR pre-post[tw] OR (pre-test[tw] AND post-test[tw]) NOT (animals[MeSH] NOT human[MeSH])
#3	(HIV Infections[MeSH] OR HIV[MeSH] OR hiv[tiab] OR hiv-1[tiab] OR hiv-2*[tiab] OR hiv1[tiab] OR hiv2[tiab] OR hiv infect*[tiab] OR human immunodeficiency virus[tiab]OR human immune deficiency virus[tiab] OR human immuno-deficiency virus[tiab] OR human immune-deficiency virus[tiab] OR ((human immun*) AND(deficiency virus[tiab])) OR acquired immunodeficiency syndromes[tiab] OR acquired immune deficiency syndrome[tiab] OR acquired immuno-deficiency syndrome[tiab] OR acquired immune-deficiency syndrome[tiab] OR ((acquired immun*) AND (deficiency syndrome[tiab])) or “sexually transmitted diseases, viral”[mh]) OR HIV[tiab] OR HIV/AIDS[tiab] OR HIV-infected[tiab] OR HIV[title] OR HIV/AIDS[title] OR HIV-infected[title]
#4	(HAART[tiab] OR ART[tiab] OR cART[tiab] OR antiretroviral[tiab] OR anti-retroviral[tiab] OR anti-viral[tiab] OR antiviral [tiab] OR “Antiretroviral Therapy, Highly Active"[Mesh])
**#5**	#1 AND #2 AND #3 AND #4
Scopus	
	(HIV OR HIV/AIDS OR AIDS OR “HUMAN IMMUNODEFICIENCY” OR “ACQUIRED IMMUNODEFICIENCY)” AND TITLE-ABS-KEY (TASK-SHIFTING OR TASKSHIFTING OR (TASK* AND SHIFT*) OR TASK* OR (REFERR* AND (NURSE* OR PHARMAC*))) AND TITLE-ABS-KEY (ANTIRETROVIRAL OR ANTI-RETROVIRAL OR ART OR CART OR HAART) AND TITLE-ABS-KEY (RANDOM* OR RANDOMIZED OR RANDOMISED OR TRIAL OR COHORT* OR GROUP* OR COMPAR* OR OBSERVATIONAL OR PROSPECTIVE* OR RETROSPECTIVE* OR “SYSTEMATIC REVIEW” OR “META-ANALYSIS)”
Web of Science	
	(TS=(HIV OR HIV/AIDS OR AIDS OR “HUMAN IMMUNODEFICIENCY” OR “ACQUIRED IMMUNODEFICIENCY)” AND TS=(TASK-SHIFTING OR TASKSHIFTING OR (TASK* AND SHIFT*) OR TASK* OR (REFERR* AND (NURSE* OR PHARMAC*))) AND TS=(ANTIRETROVIRAL OR ANTI-RETROVIRAL OR ART OR CART OR HAART) AND TS= (RANDOM* OR RANDOMIZED OR RANDOMISED OR TRIAL OR COHORT* OR GROUP* OR COMPAR* OR OBSERVATIONAL OR PROSPECTIVE* OR RETROSPECTIVE* OR “SYSTEMATIC REVIEW” OR “META-ANALYSIS)”)
OR	
	(TI=(HIV OR HIV/AIDS OR AIDS OR “HUMAN IMMUNODEFICIENCY” OR “ACQUIRED IMMUNODEFICIENCY)” AND TI=(TASK-SHIFTING OR TASKSHIFTING OR (TASK* AND SHIFT*) OR TASK* OR (REFERR* AND (NURSE* OR PHARMAC*))) AND TI=(ANTIRETROVIRAL OR ANTI-RETROVIRAL OR ART OR cART OR HAART) AND TI= (RANDOM* OR RANDOMIZED OR RANDOMISED OR TRIAL OR COHORT* OR GROUP* OR COMPAR* OR OBSERVATIONAL OR PROSPECTIVE* OR RETROSPECTIVE* OR “SYSTEMATIC REVIEW” OR “META-ANALYSIS)”)
CENTRAL	
	HIV* OR HIV-1* OR HIV-2* OR HIV1 OR HIV2 OR HIV INFECT* OR HUMAN IMMUNODEFICIENCY VIRUS OR HUMAN IMMUNEDEFICIENCY VIRUS OR HUMAN IMMUNE-DEFICIENCY VIRUS OR HUMAN IMMUNO-DEFICIENCY VIRUS OR HUMAN IMMUN* DEFICIENCY VIRUS OR ACQUIRED IMMUNODEFICIENCY SYNDROME OR ACQUIRED IMMUNEDEFICIENCY SYNDROME OR ACQUIRED IMMUNO-DEFICIENCY SYNDROME OR ACQUIRED IMMUNE-DEFICIENCY SYNDROME OR ACQUIRED IMMUN* DEFICIENCY SYNDROME in Title, Abstract, Keywords and (TASK-SHIFTING OR TASKSHIFTING OR (TASK* AND SHIFT*) OR TASK* OR (REFERR* AND (NURSE* OR PHARMAC*))) in Title, Abstract, Keywords and ANTIRETROVIRAL OR ANTI-RETROVIRAL OR ART OR cART OR HAART in Title, Abstract, Keywords
WHO Global Health Library	
	(TASK-SHIFTING OR TASKSHIFTING OR (TASK* AND SHIFT*) OR TASK* OR (REFERR* AND (NURSE* OR PHARMAC*)) AND (HIV* OR human immunodeficiency) AND (antiretroviral OR anti-retroviral))) OR (HIV AND task-shifting) OR (HIV* AND task* AND shift*)

Assessment of risk of bias in included studies

We will assess the risk of bias in RCTs using the Cochrane risk of bias assessment tool for randomised studies.[19] For non-RCT studies, we will use the Cochrane Risk of Bias Assessment Tool for Non-Randomised Studies of Interventions (ACROBAT-NRSI).[20]

For peer review only - http://bmjopen.bmj.com/site/about/guidelines.xhtml

Downloaded from http://bmjopen.bmj.com/ on September 16, 2016 - Published by group.bmj.com

We will resolve any disagreements between the authors conducting duplicate independent screening of search outputs, assessments of study eligibility, extraction of data, and risk of bias assessment by discussion and consensus. Should this fail to resolve the differences, a third author will arbitrate.

Measures of effect

We will calculate and report risk ratios for dichotomous and time-to-event data and mean differences for continuous data with their 95% CIs.

Unit of analysis issues

The unit of analysis will be the individual study participant. Cluster-randomised trials will be included in meta-analyses only after adjustments are made for design effect. Design effects for cluster-randomised studies will be corrected by using standard procedures, using the formula: design effect=1+(m−1)r, where m is the average cluster size and r is the intra-cluster correlation coefficient.

Dealing with missing data

We will contact study authors if it is necessary to obtain data missing from published reports.

Assessment of heterogeneity

We will examine statistical heterogeneity between study results using the χ^2 test of homogeneity, with a significance α-level of 0.1. In addition, we will use the I^2 statistic to measure the amount of heterogeneity among the trials in each analysis. If we identify significant heterogeneity (ie, p<0.1), we will explore it by prespecified subgroup analysis. If heterogeneity persists, we will perform sensitivity analyses, report results separately and propose reasons for the observed heterogeneity.

Assessment of reporting biases

If any meta-analysis in our review includes 10 or more studies, we will assess the potential for publication bias using a funnel plot.[21] We will attempt to minimise the potential for publication bias through a comprehensive search of published and unpublished literature.

Data synthesis

We will conduct meta-analysis, if appropriate, using the Cochrane Review Manager software (RevMan [Computer program] The Nordic Cochrane Centre, *The Cochrane Collaboration, Copenhagen*, 2015; Version 5.3). If we find no significant statistical heterogeneity of effects, we will use the fixed effect method of meta-analysis. Otherwise, we will use the random effects model.

Subgroup analysis

In pooled results with significant statistical heterogeneity, we will explore the cause of the heterogeneity through subgroup analyses, with subgroups defined by type of intervention (eg, cadre of health provider), comparison group and region of study (eg, sub-Saharan Africa, Southeast Asia, etc).

Sensitivity analysis

We will conduct a sensitivity analysis to investigate the effect of excluding studies with high risk of bias, with a focus on bias introduced by inadequate allocation concealment, inadequate blinding of outcome assessment and substantial losses to follow-up.

Certainty of evidence

We will assess the certainty (or quality) of evidence using the Grading of Recommendations, Assessment, Development and Evaluation (GRADE) approach,[22] which defines the certainty of evidence for each outcome as "the extent of our confidence that the estimates of effect are correct".[19] The quality rating across studies has four levels: high, moderate, low and very low. Randomised trials are considered to be of high quality but can be downgraded for any of five reasons: risk of bias, indirectness of evidence, unexplained heterogeneity of effects, imprecision of effect estimates and high probability of publication bias. Similarly, observational studies are considered to be of low quality, but can be upgraded for any of three reasons. The quality level of a body of evidence can be increased if there is a large magnitude of effect, if all plausible confounding would reduce a demonstrated effect, and if there is a dose-response gradient.

Reporting of this review

The findings of this review will be presented in a number of ways. The study selection process will be summarised using a flow diagram, and if we identify 10 or more eligible studies, we will assess publication bias using funnel plots. Where appropriate, we will use risk of bias graphs, forest plots and GRADE summary of findings tables. The non-quantitative outcomes will be reported descriptively. We will provide tables of both included and excluded studies. We have prepared this protocol as recommended by the PRISMA-P (Preferred Reporting Items for Systematic Review and Meta-Analysis Protocols) guidelines[23] and will report the findings of the review as recommended by the PRISMA statement.[24]

ETHICS AND DISSEMINATION

Since systematic reviews do not directly involve human participants, they do not require ethical clearance.[25] We will provide the findings of this review to the WHO, with the hope that they may guide policy recommendations of this normative agent regarding the shifting of ART dispensing or distribution from pharmacy to non-pharmacy personnel. Although the majority of national programmes in low and middle-income countries have adopted task shifting in ART care at different levels,

For peer review only

For peer review only - http://bmjopen.bmj.com/site/about/guidelines.xhtml

Downloaded from http://bmjopen.bmj.com/ on September 16, 2016 - Published by group.bmj.com

there has been no global policy to guide the practice for task shifting from pharmacy to non-pharmacy personnel. We will also publish the findings of the systematic review in a peer-reviewed journal.

Twitter Follow Charles Wiysonge at @CharlesShey

Acknowledgements The authors acknowledge the Editor and the referees for critical and constructive comments on earlier versions of this manuscript. The authors would also like to acknowledge the various contributions of Eyerusalem Negussie.

Contributors NMM led the development of the protocol, wrote the first draft, coordinated and integrated comments from co-authors and approved the final version for publication. TK and CSW conceived the study, critically revised successive drafts of the manuscript, and approved the final version for publication. CSW is the guarantor of the manuscript.

Funding Funding for preparing this review was provided by the Department of HIV/AIDS at the WHO Headquarters in Geneva, Switzerland. The authors would like to thank Hacsi Horvath (Global Health Sciences, University of California, San Francisco, USA) for his assistance in developing the search strategy for electronic databases. Neither the authors' institutions nor the funder played a role in preparing the manuscript and the views expressed therein are solely those of the authors.

Competing interests None declared.

Provenance and peer review Not commissioned; externally peer reviewed.

Open Access This is an Open Access article distributed in accordance with the terms of the Creative Commons Attribution (CC BY 4.0) license, which permits others to distribute, remix, adapt and build upon this work, for commercial use, provided the original work is properly cited. See: http://creativecommons.org/licenses/by/4.0/

REFERENCES

1. UNAIDS. How AIDS changed everything—MDG 6: 15 years, 15 lessons of hope from the AIDS response. https//:www.unaids.org/en/resources/documents/2015/MDG6_15years-15lessonsfromtheAIDSresponse (accessed 22 Sep 2015).
2. Palella FJ, Delaney KM, Moorman AC, *et al*. Declining morbidity and mortality among patients with advanced human immunodeficiency virus infection. HIV Outpatient Study Investigators. *N Engl J Med* 1998;338:853–60.
3. Anglemyer A, Rutherford GW, Horvath T, *et al*. Antiretroviral therapy for prevention of HIV transmission in HIV-discordant couples. *Cochrane Database Syst Rev* 2013;4:CD009153.
4. Ford N, Calmy A, Mills EJ. The first decade of antiretroviral therapy in Africa. *Global Health* 2011;7:33.
5. King RC, Fomundam HN. Remodeling pharmaceutical care in sub-Saharan Africa (SSA) amidst human resources challenges and the HIV/AIDS pandemic. *Int J Health Plann Manage* 2010;25:30–48.
6. WHO. *Increasing access to health workers in remote and rural areas through improved retention: global policy recommendations*. Geneva, Switzerland: World Health Organisation, 2010.
7. SAPC. Pharmacy Human Resources data. https//:www.healthlink.org.za/healthstats/283/data (accessed 15 Aug 2015).
8. Saberi P, Dong BJ, Johnson MO, *et al*. The impact of HIV clinical pharmacists on HIV treatment outcomes: a systematic review. *Patient Prefer Adherence* 2012;6:297–322.
9. Tseng A, Foisy M, Hughes CA, *et al*. Role of pharmacist in caring for patients with HIV/AIDS: clinical practice guidelines. *Can J Hosp Pharm* 2012;65:125–45.
10. WHO. *The world of medicines situation 2011: pharmaceutical human resources*. Geneva, Switzerland: World Health Organisation, 2011.
11. Kredo T, Adeniyi FB, Bateganya M, *et al*. Task shifting from doctors to non-doctors for initiation and maintenance of antiretroviral therapy. *Cochrane Database Syst Rev* 2014;7:CD007331.
12. Kitahata MM, Koepsell TD, Deyo RA, *et al*. Physicians experience with the acquired immunodeficiency syndrome as a factor in patients' survival. *N Engl J Med* 1996;334:701–6.
13. Landon BE, Wilson IB, McInnes K, *et al*. Physician specialization and the quality of care for human immunodeficiency virus infection. *Arch Intern Med* 2005;165:1133–9.
14. Decroo T, Telfer B, Biot M, *et al*. Distribution of antiretroviral treatment through self-forming groups of patients in Tete province, Mozambique. *J Acquir Immune Defic Syndr* 2011;56:e39–44.
15. Kipp W, Konde-Lule J, Rubaale T, *et al*. Comparing antiretroviral treatment outcomes between a prospective community-based and hospital-based cohort of HIV patients in rural Uganda. *BMC Int Health Hum Rights* 2011;11(Suppl 2):S12.
16. Selke HM, Kimaiyo S, Sidle JE, *et al*. Task-shifting of antiretroviral delivery from health care workers to persons living with HIV/AIDS: clinical outcomes of a community-based program in Kenya. *J Acquir Immune Defic Syndr* 2010;55:483–90.
17. Callaghan M, Ford N, Schneider H. A systematic review of task-shifting for HIV treatment and care in Africa. *Hum Resour Health* 2010;8:8.
18. Laurant M, Reeves D, Hermens R, *et al*. Substitution of doctors by nurses in primary care. *Cochrane Database Syst Rev* 2005;CD001271.
19. Higgins JPT, Green S, eds. *Cochrane handbook for systematic reviews of interventions*. Wiley-Blackwell, 2008.
20. Cochrane Collaboration. A Cochrane Risk of Bias Assessment Tool: for Non-Randomized studied of Interventions (ACROBAT-NRSI). https://www.riskofbias.info/ (accessed 30 Oct 2015).
21. Egger M, Davey Smith G, Schneider M, *et al*. Bias in meta-analysis detected by a simple, graphical test. *BMJ* 1997;315:629–34.
22. Guyatt G, Oxman AD, Akl EA, *et al*. GRADE guidelines: 1. Introduction-GRADE evidence profiles and summary of findings tables. *J Clin Epidemiol* 2011;64:383–94.
23. Shamseer L, Moher D, Clarke M, *et al*; PRISMA-P Group. Preferred reporting items for systematic review and meta-analysis protocols (PRISMA-P). *BMJ* 2015;349:g7647.
24. Liberati A, Altman DG, Tetzlaff J, *et al*. The PRISMA Statement for reporting systematic reviews and meta-analyses of studies that evaluate healthcare interventions: explanation and elaboration. *BMJ* 2009;339:b2700.
25. Emanuel EJ, Wendler D, Killen J, *et al*. What makes clinical research in developing countries ethical? The benchmarks of ethical research. *J Infect Dis* 2004;189:930–7.

For peer review only

For peer review only - http://bmjopen.bmj.com/site/about/guidelines.xhtml

Downloaded from http://bmjopen.bmj.com/ on September 16, 2016 - Published by group.bmj.com

The effects of shifting tasks from pharmacy to non-pharmacy personnel for providing antiretroviral therapy to people living with HIV: a systematic review protocol

Nyanyiwe M Mbeye, Tamara Kredo and Charles S Wiysonge

BMJ Open 2016 6:
doi: 10.1136/bmjopen-2015-008195

Updated information and services can be found at:
http://bmjopen.bmj.com/content/6/3/e008195

These include:

References This article cites 18 articles, 4 of which you can access for free at:
http://bmjopen.bmj.com/content/6/3/e008195#BIBL

Open Access This is an Open Access article distributed in accordance with the terms of the Creative Commons Attribution (CC BY 4.0) license, which permits others to distribute, remix, adapt and build upon this work, for commercial use, provided the original work is properly cited. See:
http://creativecommons.org/licenses/by/4.0/

Email alerting service Receive free email alerts when new articles cite this article. Sign up in the box at the top right corner of the online article.

Topic Collections Articles on similar topics can be found in the following collections

Evidence based practice (557)
Health services research (1106)
HIV AIDS (159)

Notes

To request permissions go to:
http://group.bmj.com/group/rights-licensing/permissions

To order reprints go to:
http://journals.bmj.com/cgi/reprintform

To subscribe to BMJ go to:
http://group.bmj.com/subscribe/

BMJ Open

Shifting tasks from pharmacy to non-pharmacy personnel for providing antiretroviral therapy to people living with HIV: A systematic review and meta-analysis

Journal:	*BMJ Open*
Manuscript ID	bmjopen-2016-015072.R2
Article Type:	Research
Date Submitted by the Author:	16-Jun-2017
Complete List of Authors:	MBEYE, NYANYIWE; Stellenbosch University, Centre for Evidence Based Health Care; College of Medicine, School of Public Health and Family Medicine<br>Adetokunboh, Olatunji; Stellenbosch University, Centre for Evidence Based Health Care; South African Medical Research Council, Cochrane South Africa<br>Negussie, Eyerusalem; World Health Organisation, HIV/AIDS<br>Kredo, Tamara; South African Medical Research Council, Cochrane South Africa; Stellenbosch University Faculty of Medicine and Health Sciences, Division of clinical Pharmacology<br>Wiysonge, Charles; Stellenbosch University, Centre for Evidence-Based Health Care; South African Medical Research Council, Cochrane South Africa
**Primary Subject Heading**:	Evidence based practice
Secondary Subject Heading:	Health services research, HIV/AIDS, Evidence based practice
Keywords:	Task shifting, HIV, antiretroviral therapy, dispensing, distribution, pharmacy personnel

SCHOLARONE™ Manuscripts

Shifting tasks from pharmacy to non-pharmacy personnel for providing antiretroviral therapy to people living with HIV: A systematic review and meta-analysis

Nyanyiwe M. Mbeye[1,2&5*], Olatunji Adetokunboh[1&2], Eyerusalem Negussie[3], Tamara Kredo[1&4], Charles S. Wiysonge[1&2],

[1] Cochrane South Africa, South African Medical Research Council, Cape Town, South Africa.
[2] Centre for Evidence-based Health Care, Faculty of Medicine and Health Sciences, Stellenbosch University, Cape Town, South Africa.
[3] Department of HIV/AIDS, World Health Organization, Geneva, Switzerland
[4] Division of Clinical Pharmacology, Faculty of Medicine and Health Sciences, Stellenbosch University, Cape Town, South Africa.
[5] College of Medicine, University of Malawi, Blantyre, Malawi.

***Correspondence author:** Dr Nyanyiwe M. Mbeye (nyanyiwembeye@gmail.com), Telephone Number: +265 888356338

For peer review only

Abstract

Objectives

Lay people or non-pharmacy health workers with training could dispense antiretroviral therapy (ART) in resource-constrained countries; freeing up time for pharmacists to focus on more technical tasks. We assessed the effectiveness of such task-shifting in low and middle-income countries.

Method

We conducted comprehensive searches of peer-reviewed and grey literature. Two authors independently screened search outputs, selected controlled trials, extracted data and resolved discrepancies by consensus. We performed random-effects meta-analysis and assessed certainty of evidence using GRADE.

Results

Three studies with 1993 participants met the inclusion criteria, including two cluster trials conducted in Kenya and Uganda and an individually randomised trial conducted in Brazil. We found very low certainty evidence regarding mortality due to the low number of events. Therefore, we are uncertain whether there is a true increase in mortality as the effect size suggests, or a reduction in mortality between pharmacy and non-pharmacy models of dispensing ART (RR 1.86, 95% confidence interval (CI) 0.44 to 7.95, n = 1993, 3 trials, very low certainty evidence). There may be no differences between pharmacy and non-pharmacy models of dispensing ART on virological failure (RR 0.92, 95% CI 0.73 to 1.15, n = 1993, 3 trials, low certainty evidence) and loss to follow-up (RR 1.13, 95% CI 0.68 to 1.91, n = 1993. 3 trials, low

For peer review only

certainty evidence). We found some evidence that costs may be reduced for the patient and health system when task-shifting is undertaken.

Conclusions

The low certainty regarding the evidence implies a high likelihood that further research may find the effects of the intervention to be substantially different from our findings. If resource-constrained countries decide to shift ART dispensing and distribution from pharmacy to non-pharmacy personnel, this should be accompanied by robust monitoring and impact evaluation.

Key words: Task-shifting, HIV, antiretroviral therapy, dispensing, distribution, and pharmacy personnel.

Strengths and limitations

- To our knowledge, this is the first published systematic review reporting the effects of task shifting from pharmacy to non-pharmacy personnel for dispensing or distributing antiretroviral therapy to patients living with HIV.
- The review was written according to the PRISMA (Preferred Reporting Items for Systematic Review and Meta-Analysis) Recommendations for reporting systematic reviews.
- The review findings may help to inform antiretroviral therapy guidelines by the World Health Organization (WHO).
- A limitation of the review is paucity of evidence in this field and use of indirect evidence to inform our results.

For peer review only

Background

Description of the condition

By March 2015, 15 million (40.7%) of the estimated 36.9 million people living with HIV (PLHIV) globally, were receiving antiretroviral therapy (ART) in low and middle-income countries [1]. Combination ART is effective for reducing HIV-related morbidity and mortality as well as preventing HIV transmission [2]. Initiating ART early in the course of HIV infection has been associated with better health outcomes, both at patient and population levels [3 4]. Scale up of ART in low and middle-income countries has averted more than 5 million deaths; however, bottlenecks preventing universal access to ART still exist. One challenge is the critical shortage of human resources for health (HRH), including for delivery of essential pharmacy services.

As for pharmacists, although the World Health Organization (WHO) recommends a minimum of one pharmacist per 2300 population [5], most countries in low-resource settings such as sub-Saharan Africa do not meet this target. In addition to the absolute shortage, it is likely that there is an uneven distribution of pharmacists in such settings, as is the case with other specialist healthcare workers who tend to concentrate in urban areas and the private sector, further aggravating the HRH shortage. For instance in South Africa, which is home to the largest number of PLHIV in any country worldwide, in 2010 only 24% of registered pharmacists worked in the public sector where 80% of the population received care [6].

Description of the intervention

Studies and programmes report that involvement of pharmacy personnel in HIV care results in improved patient outcomes [7]. For instance, in the United States of America (USA), the use of a multidisciplinary team approach with pharmacists assuming a

For peer review only - http://bmjopen.bmj.com/site/about/guidelines.xhtml

central role in the initiation, dispensing and adherence counselling improved treatment outcomes such as viral load, patient retention and medication adherence [8]. The work of pharmacists includes supply management, dispensing and distributing medications, promoting adherence, identifying and preventing potential medication-related issues (such as over dosage, sub-therapeutic dosage, adverse drug reactions, medication errors and untreated indications), and monitoring and reporting adverse drug events [9 10]. In some settings, programmes have implemented alternative models of pharmacy services that shift selected tasks from pharmacy to non-pharmacy personnel. Such alternative models could potentially increase the number of health workers involved in ART distribution, adherence counselling and patient education; free more time for pharmacy personnel; support the integration of ART in primary care settings; minimise the number of facility visits for ART collection; and, reduce pharmacy queue waiting times for patients.

The specifics of shifting ART-related tasks from pharmacy personnel have not been addressed in a systematic review. Available systematic reviews on task shifting focus on clinical services where nurses and non-physician clinicians provide care comparable to physicians [11]. We therefore synthesised the evidence for task shifting in pharmacy personnel services, where non-pharmacy personnel undertake ART distribution and medication adherence counselling. Table 1 provides definitions for the pharmacy functions distribution and dispensing as they have been used in this review. However, these definitions seem to overlap at facility level.

Table 1: Definitions and descriptions of dispensing and distribution of medications

Term	Definition
Dispensing	• Dispensing is a controlled act that authorises one to select, prepare and provide stock medication that has been prescribed to a patient or client (or his/her representative) for

For peer review only

	administration at a later time[12] .<br>• To dispense is to prepare and supply to a patient a course of therapy on the basis of a prescription[13] .<br>• Dispensing is preparation and distribution of a course of therapy to a patient, with appropriate instructions based on a prescription [13].
Distribution	• At facility level, drug distribution mainly refers to drugs dispensed by licensed practitioners, such as nurses, doctors, pharmacists or pharmacy assistants and collected by a patient.<br>• At community level, drug distribution refers to trained lay people collecting pre-packed medications from the facility and delivering them to HIV patients in the community [12].

How task shifting using non-pharmacy personnel might work

Within the last decade, several high HIV burden countries adopted task shifting strategies where nurses and non-physician clinicians initiate and maintain ART. Although this has undeniably expanded access to ART, it is also increasingly essential that long facility waiting times and frequent facility visits to collect ART are addressed to alleviate the burden of care, both for patients and healthcare providers [9 10].

Recent studies in Uganda, Kenya and Mozambique have shown positive outcomes when non-health professionals (lay people) delivered ART at the community level [14]. In Mozambique, the use of PLHIV for distributing ART, monitoring adherence, reporting outcomes and referring sick patients to health facilities yielded a retention rate of 97.5% among stable patients on ART [14]. In a cluster randomised trial in Uganda, the use of community health workers produced comparable results with the facility-based ART programme in terms of patient retention, viral load suppression and mortality rate [15]. Similar findings were also obtained in Kenya and some other parts of Uganda when lay providers were engaged in ART delivery [15 16].

For peer review only

For peer review only - http://bmjopen.bmj.com/site/about/guidelines.xhtml

Task shifting has therefore been seen as an achievable solution to the critical human resource shortages affecting scale up of ART [17]. While it is imperative to increase the rate of recruitment and training of health workers as well as improve working conditions to reduce attrition and emigration, the HIV pandemic requires that all possible options are considered to address the critical skills shortage [18]. Such measures may include shifting selected tasks from pharmacy to non-pharmacy personnel, particularly for patients stable on their ART. Task-shifting allows more time for pharmacy personnel to focus on more technical functions such as supply management, pharmacovigilance and patient consultation.

Why this review is important

Dependence on and shortage of pharmacists are key constraints on ART expansion, but the specifics of task shifting for ART distribution from pharmacy to non-pharmacy personnel have not been reviewed systematically. Previous systematic reviews of task shifting for increasing ART access focused on clinical services where nurses and non-physician clinicians provide care. We systematically reviewed the scientific literature and assessed the efficacy of task-shifting models that use non-pharmacy and pharmacy personnel in distributing ART and assessing adherence to treatment of HIV infection.

Objective

This review evaluated the efficacy and safety of shifting pharmacy related tasks, including ART distribution and adherence assessment, from pharmacy to non-pharmacy personnel.

For peer review only

Methods

The review was registered in PROSPERO International Prospective Register of Systematic reviews (http://www.crd.york.ac.uk/PROSPERO), registration number CRD42015017034. The protocol was published in the BMJ Open [19].

Criteria for considering studies for this review

Types of studies: Randomised controlled trials (RCTs) or observational studies with control arms conducted in low and middle-income countries.

Types of participants*:* People living with HIV receiving ART.

Types of interventions: We included studies that evaluated shifting of selected tasks from pharmacy to non-pharmacy personnel. Tasks include distribution, dispensing of ART and adherence to ART as shown in Table 1. Pharmacy personnel included pharmacists and pharmacy technicians while non-pharmacy personnel included patient peer groups, community volunteers, people living with HIV, community health committees, nurses, physicians and non-physician clinicians.

For peer review only

Types of outcome measures*:*

Primary outcome: mortality

Secondary outcomes: virological suppression; number of all-cause sick visits made to the health facility including adverse events; loss to follow-up; adherence to ART (as measured within the study, e.g. pill counts, recall methods, digital methods, self report); acceptability to pharmacy personnel, non-pharmacy personnel, patients; cost and harm including rates of errors.

Search methods for identification of studies

We performed a comprehensive search of electronic databases and conference proceedings to identify all relevant studies up until 10 May 2016, regardless of language of publication or publication status (published, unpublished, in press and in progress). We searched Cochrane Central Register of Controlled Trials (CENTRAL), Excerpta Medica Database (EMBASE), PubMed, ISI Web of Science (Science Citation index), and World Health Organization (WHO) Global Health Library.

We used appropriate Medical Subject Heading (MeSH) terms, relevant keywords, and validated terms for identifying reports of randomised controlled trials [20]. Search strategies are reported in Table 2. We searched conference abstract archives of the Conference on Retroviruses and Opportunistic Infections (CROI), the International AIDS Conference (IAC), and the International AIDS Society Conference on HIV Pathogenesis, Treatment and Prevention (IAS), up to 10 May 2016. We contacted experts and organisations for additional studies.

For peer review only

Table 2: Search strategy for electronic databases

ID	Search terms
**PubMed**	
#1	(task*[tiab] OR task-shifting[tiab] OR referr*[tiab] OR referral and consultation[mh] OR role*[tiab]) AND (health personnel[mh] OR doctor[tiab] OR doctors[tiab] OR clinician[tiab] OR clinicians[tiab] OR physician[tiab] OR physicians[tiab] OR "healthcare provider"[tiab] OR "healthcare providers"[tiab] OR "health care provider"[tiab] OR "health care providers"[tiab] OR pharmac*[tiab] OR apothecar*[tiab] OR chemist*[tiab] OR dispensar*[tiab])
#2	randomized controlled trial[pt] OR controlled clinical trial[pt] OR randomized controlled trials[MeSH] OR random allocation[MeSH] OR double-blind method[MeSH] OR single-blind method[MeSH] OR clinical trial[pt] OR clinical trials[MeSH] OR ("clinical trial"[tw]) OR ((singl*[tw] OR doubl*[tw] OR trebl*[tw] OR tripl*[tw]) AND (mask*[tw] OR blind*[tw])) OR random*[tw] OR research design[mh:noexp] OR prospective studies[MeSH] OR control*[tw] OR volunteer*[tw]) OR observational[tw] OR non-random*[tw] OR nonrandom*[tw] OR before after study[tw] OR time series[tw] OR cohort*[tw] OR cross-section*[tw] OR prospective*[tw] OR retrospective*[tw] OR research design[mh:noexp] OR follow-up studies[MeSH] OR longitud*[tw] OR evaluat*[tiab] OR pre-post[tw] OR (pre-test[tw] AND post-test[tw]) NOT (animals[MeSH] NOT human[MeSH])
#3	(HIV Infections[MeSH] OR HIV[MeSH] OR hiv[tiab] OR hiv-1[tiab] OR hiv-2*[tiab] OR hiv1[tiab] OR hiv2[tiab] OR hiv infect*[tiab] OR human immunodeficiency virus[tiab]OR human immune deficiency virus[tiab] OR human immuno-deficiency virus[tiab] OR human immune-deficiency virus[tiab] OR ((human immun*) AND(deficiency virus[tiab])) OR acquired immunodeficiency syndromes[tiab] OR acquired immune deficiency syndrome[tiab] OR acquired immuno-deficiency syndrome[tiab] OR acquired immune-deficiency syndrome[tiab] OR ((acquired immun*) AND (deficiency syndrome[tiab])) or “sexually transmitted diseases, viral”[mh]) OR HIV[tiab] OR HIV/AIDS[tiab] OR HIV-infected[tiab] OR HIV[title] OR HIV/AIDS[title] OR HIV-infected[title]
#4	(HAART[tiab] OR ART[tiab] OR cART[tiab] OR antiretroviral[tiab] OR anti-retroviral[tiab] OR anti-viral[tiab] OR antiviral[tiab] OR "Antiretroviral Therapy, Highly Active"[Mesh])
**#5**	#1 AND #2 AND #3 AND #4
**Scopus**	
	(HIV OR HIV/AIDS OR AIDS OR "HUMAN IMMUNODEFICIENCY" OR "ACQUIRED IMMUNODEFICIENCY") AND TITLE-ABS-KEY (TASK-SHIFTING OR TASKSHIFTING OR (TASK* AND SHIFT*) OR TASK* OR (REFERR* AND (NURSE* OR PHARMAC*))) AND TITLE-ABS-KEY (ANTIRETROVIRAL OR ANTI-RETROVIRAL OR ART OR CART OR HAART) AND TITLE-ABS-KEY (RANDOM* OR RANDOMIZED OR RANDOMISED OR TRIAL OR COHORT* OR

	GROUP* OR COMPAR* OR OBSERVATIONAL OR PROSPECTIVE* OR RETROSPECTIVE* OR "SYSTEMATIC REVIEW" OR "META-ANALYSIS")
**Web of Science**	
	(TS=(HIV OR HIV/AIDS OR AIDS OR "HUMAN IMMUNODEFICIENCY" OR "ACQUIRED IMMUNODEFICIENCY") AND TS=(TASK-SHIFTING OR TASKSHIFTING OR (TASK* AND SHIFT*) OR TASK* OR (REFERR* AND (NURSE* OR PHARMAC*))) AND TS=(ANTIRETROVIRAL OR ANTI-RETROVIRAL OR ART OR CART OR HAART) AND TS=(RANDOM* OR RANDOMIZED OR RANDOMISED OR TRIAL OR COHORT* OR GROUP* OR COMPAR* OR OBSERVATIONAL OR PROSPECTIVE* OR RETROSPECTIVE* OR "SYSTEMATIC REVIEW" OR "META-ANALYSIS")) <br>**OR** <br>(TI=(HIV OR HIV/AIDS OR AIDS OR "HUMAN IMMUNODEFICIENCY" OR "ACQUIRED IMMUNODEFICIENCY") AND TI=(TASK-SHIFTING OR TASKSHIFTING OR (TASK* AND SHIFT*) OR TASK* OR (REFERR* AND (NURSE* OR PHARMAC*))) AND TI=(ANTIRETROVIRAL OR ANTI-RETROVIRAL OR ART OR cART OR HAART) AND TI=(RANDOM* OR RANDOMIZED OR RANDOMISED OR TRIAL OR COHORT* OR GROUP* OR COMPAR* OR OBSERVATIONAL OR PROSPECTIVE* OR RETROSPECTIVE* OR "SYSTEMATIC REVIEW" OR "META-ANALYSIS"))
**CENTRAL**	
	HIV* OR HIV-1* OR HIV-2* OR HIV1 OR HIV2 OR HIV INFECT* OR HUMAN IMMUNODEFICIENCY VIRUS OR HUMAN IMMUNEDEFICIENCY VIRUS OR HUMAN IMMUNE-DEFICIENCY VIRUS OR HUMAN IMMUNO-DEFICIENCY VIRUS OR HUMAN IMMUN* DEFICIENCY VIRUS OR ACQUIRED IMMUNODEFICIENCY SYNDROME OR ACQUIRED IMMUNEDEFICIENCY SYNDROME OR ACQUIRED IMMUNO-DEFICIENCY SYNDROME OR ACQUIRED IMMUNE-DEFICIENCY SYNDROME OR ACQUIRED IMMUN* DEFICIENCY SYNDROME in Title, Abstract, Keywords and (TASK-SHIFTING OR TASKSHIFTING OR (TASK* AND SHIFT*) OR TASK* OR (REFERR* AND (NURSE* OR PHARMAC*))) in Title, Abstract, Keywords and ANTIRETROVIRAL OR ANTI-RETROVIRAL OR ART OR cART OR HAART in Title, Abstract, Keywords
**WHO Global Health Library**	
	(TASK-SHIFTING OR TASKSHIFTING OR (TASK* AND SHIFT*) OR TASK* OR (REFERR* AND (NURSE* OR PHARMAC*)) AND (HIV* OR human immunodeficiency) AND (antiretroviral OR anti-retroviral))) OR (HIV AND task-shifting) OR (HIV* AND task* AND shift*)

For peer review only

Data collection and analysis

Data collection and analysis approach was based on standards from the Cochrane Handbook of Systematic Reviews of Interventions [20].

Selection of studies for inclusion

Two review authors (NMM and OA) screened the titles, abstracts of all records to identify potentially eligible reports and then independently inspected potentially eligible publications for inclusion. Differences were discussed with a third author (CSW or TK) and resolved by discussion.

Data extraction and management

Two review authors (NMM and OA) independently extracted data onto standardised, pre-piloted data extraction forms. The following characteristics were extracted from each included study:

- **Study details:** Citations of publications associated with the study, start and end dates, location, study design characteristics, type of facility involved, investigators, funding sources, recruitment, method of randomisation, sequence generation, method of allocation concealment, blinding of participants and personnel, blinding of outcome assessors, length of follow up, losses to follow up, withdrawals or drop-outs and other relevant details.
- **Details of the intervention:** details of the cadre of health worker, what training or other support or supervision they received and other relevant details.

For peer review only

- **Details of participants:** Trial inclusion and exclusion criteria, numbers of participants entering the trial, sex, clinical staging, CD4 count and other pertinent details.
- **Outcome details:** Definitions of outcomes, details of how outcomes were assessed, numerators and denominators associated with each outcome, completeness of outcome data, effect estimates reported and other relevant outcome information.

Assessment of risk of bias in included controlled trials

Two review authors (NMM and OA) independently assessed the risk of bias in each study using the Cochrane risk of bias tool which measures risk of bias in controlled trials across seven domains: sequence generation, allocation concealment, blinding of participants and personnel, blinding of outcome assessors, completeness of outcome data, selective outcome reporting and other potential biases [20].

Measures of effect

We calculated and presented summary statistics for the risk ratio (RR) for dichotomous outcomes with their 95% confidence intervals.

Unit of analysis issues

The unit of analysis was the individual study participant for all the trials.

Dealing with missing data

We contacted study authors for one of the included trials to obtain information on the composition of health professionals in the control groups involved in dispensing ART in order to establish whether pharmacy personnel were part of the team. However, the

For peer review only

author no longer had those details but assumed that health professionals included pharmacy personnel.

Assessment of heterogeneity

We used the I^2 statistic to measure heterogeneity among the trials. We planned to explore substantial heterogeneity (I^2 >50%) by pre-specified subgroup analysis. However, there was no evidence of serious heterogeneity.

Assessment of reporting biases

We minimised the potential for publication bias by using a comprehensive search strategy. As none of the meta-analysis include 10 or more studies, we did not assess publication bias using a funnel plot [20 21].

Data synthesis

Meta-analysis was conducted using Review Manager software [22]. We used a random effects model considering the diverse settings of the included studies. Where meta-analysis was not possible, for instance, for adherence and cost, a narrative synthesis of the evidence was carried out. We summarised the certainty of evidence for each outcome using GRADE [23 24]. The GRADE system defines the certainty of evidence for each outcome as "the extent of our confidence that the estimates of effect are correct" [20]. The quality rating across studies has four levels: high, moderate, low or very low. Randomised trials are considered to be of high quality but can be downgraded for any of the following five reasons: risk of bias; indirectness of evidence; unexplained heterogeneity or inconsistency of results; imprecision of results; and, high probability of publication bias. Similarly, observational studies are considered to be of low quality, but can be upgraded for any of these three reasons: large magnitude of effect; all plausible confounding would reduce a demonstrated

For peer review only

effect; and, the presence of a dose-response gradient. We independently considered the five factors for downgrading the evidence in included trials.

Subgroup analysis and sensitivity analysis

Sensitivity analyses were not conducted to investigate the effect of excluding studies with high or low risk of bias due to the small number of studies included in the review. No sub-group analyses were indicated by the data.

Results

A total of 3557 records were identified. Following title and abstract screening, eight studies were identified for full-text screening. Three trials met the inclusion criteria [16 25 26]. The study selection process is shown in Figure 1. The characteristics of included and excluded studies are shown in Tables 3 and 4 respectively.

Overall, 1993 HIV-infected patients on ART were included. Of these, 1121 were assigned to non-pharmacy personnel model of ART delivery while 872 were assigned to pharmacy personnel model of ART delivery.

Two studies [16], [25] included lay people in the non-pharmacy personnel group while the third [26] included nurses in the non-pharmacy personnel group as shown in Table 3.

For peer review only

300 **Table 3: Summary of included studies**

Study ID	Setting	Methods	Outcomes
Selke 2010 [16]	Kenya	**Study design:** Cluster randomised trial<br><br>**Description of interventions:**<br>**Non-pharmacy personnel (home-based) group:**<br>Community Care Counsellors were:<br>• Clinically stable patients with self-reported 100% adherence to ART over the six-month period before recruitment.<br>• A patient considered by the clinic staff to be good role model and mentor for other patients.<br>Duties for care givers during home visits:<br>• Obtained and entered data concerning patients' symptoms.<br>• Vital sign assessment.<br>• Assessment of adherence to ART and opportunistic infections prophylaxis.<br>• Distributed a one-month supply of the patients' medications (from a prefilled kit).<br><br>**Pharmacy personnel (facility-based) group:**<br>• Nurses/clinical officers or physicians performed the following:<br>o Took an interim medical history.<br>o Addressed any acute concerns.<br>o Reviewed medications.<br>o Prescribed ART and opportunistic infection prophylaxis.<br>• Patients collected a one-month supply of their medication dispensed from the pharmacy.	• Adherence<br>• Viral load responses<br>• Intercurrent opportunistic infections<br>• Hospitalisation<br>• Loss to follow up<br>• Change in second-line therapy<br>• Mortality
Jaffar 2009 [25]	Uganda	**Study design:** Cluster-randomised trial	• Plasma RNA VL>500 copies per

For peer review only - http://bmjopen.bmj.com/site/about/guidelines.xhtml

		**Description of interventions:**<br>**Non-pharmacy personnel (home-based care) group:**<br>• Field workers with degree or college diploma who received a week of intensive training at start of study and yearly refresher courses on:<br>o The principles of antiretroviral therapy.<br>o Adherence support.<br>• Field workers visited patients in the homes on motor bikes every month to:<br>o Deliver drugs.<br>o Monitor participants with a checklist of signs and symptoms of drug toxicity and disease progression.<br>o Provide adherence support.<br>o Referred patients to physician or counsellor at the clinic when judged necessary.<br>• At two and six months after starting therapy, all patients were reviewed by a Medical Officer at the clinic and then at the twelfth month.<br>• Drugs were not dispensed during the clinic visits for this group.<br>• Patients were visited again when not found at home.<br>• Patients asked to come to clinic when unwell.<br><br>**Pharmacy personnel (facility-based) group:**<br>• Patients collected drugs every month from the pharmacy.<br>• Routine reviews by a Medical Officer and counsellor at two and three months after start of treatment & every three months thereafter.<br>• Patients assessed by a nurse and referred to a doctor when necessary during clinic visits.<br>• Patients followed up at home by field workers when missed an appointment.<br>• Patients received vouchers for their households for free voluntary counselling and testing at the clinic.	mL.<br>• Either plasma RNA >500 copies per mL if undetectable at six months, or an increase of 1000 copies between two consecutive tests if RNA detectable at six months.<br>• All-cause mortality.<br>• Mortality or plasma RNA VL >500 copies per mL.<br>• Admitted on one or more occasions.<br>• All admissions.<br>• Death, first admission, or change to second-line therapy.<br>• Frequency of out-patient attendance.<br>• Adherence.<br>• Costs of health service delivery and costs incurred by patients to access care.

For peer review only

For peer review only - http://bmjopen.bmj.com/site/about/guidelines.xhtml

		• Patients asked to come to clinic when unwell.	
Silveira 2014 [26]	Brazil	**Study design:** Individually randomised trial.<br><br>**Description of interventions:**<br>**Pharmacy personnel group:**<br>Patients received this intervention on a monthly basis through the Da´der method which consisted a series of scheduled meetings where the pharmacist and patient addressed, reviewed, and solved drug-related problems for a 12-month period. Patients received structured counselling from pharmacists on their prescription regimens at the time of their initial drug dispensing and at monthly refill visits.<br>Key elements included:<br>• Reviewing the prescription with the patient.<br>• Reviewing a card on which medications were colour-coded to facilitate recognition and reduce confusion that might arise from complicated drug names.<br>• Reviewing the schedule, length and date of next appointment.<br>• Reviewing patient's understanding of the prescription by asking patient to describe it and giving patients verbal information on the expected side effects of their medications and instructing them to seek medical assistance by calling the pharmacist if side effects occurred.<br><br>**Non-pharmacy personnel group:**<br>Patients received Usual Care (UC) for ART drug distribution. The control group received UC for ART drug delivery. This included the following:<br>At the first appointment, when patients received their medications, a nurse first provided information on:<br>• The regimen,<br>• How and when to use the medications,	• Self-reported adherence calculated by taking the number of tablets that patients reported ingesting and dividing it by the number they should have ingested. Patients were classified as adherent if they reported using 95% or more of the tablets prescribed<br>• Depression: This was investigated using a validated Portuguese-language version of BDI with standard scoring

For peer review only - http://bmjopen.bmj.com/site/about/guidelines.xhtml

		• Principal side effects.<br>• Importance of adhering to the prescription.<br><br>Afterward, patients picked up their medications at a drug-dispensation counter. They had no encounters with a pharmacist. Patients were informed that they could schedule time with a nurse for any questions about their treatment or disease.<br>In both groups, patients were scheduled every four months for medical appointments and measurements of viral load and CD4 count. Participants who did not appear for their regularly scheduled appointment were contacted by telephone and asked to return.	

For peer review only

For peer review only - http://bmjopen.bmj.com/site/about/guidelines.xhtml

Table 4: Summary of excluded studies

Study ID	Reasons for exclusion
March 2007 [27]	All participants were enrolled in the pharmacist-managed HIV drug optimisation clinic, with no non-pharmacist control group. We excluded this study because we could not make the comparisons due to the absence of a non-pharmacy personnel group.
Chang 2010 [28]	Non-pharmacy personnel did not dispense or distribute ART
Kiweewa 2013 [29]	Dispensing of ART done in same way for both groups hence we could not make comparisons.
Hansudewechaku 2012 [30]	Cohort study with comparisons between community and non-community hospitals with both groups having pharmacy personnel
Henderson 2011 [31]	Observational study with no comparison group.

Risk of bias in included studies

The included trials all had low risk of selection bias as a result of adequate randomisation and allocation concealment. Selke et al [16] and Jaffar et al [25] were judged to have low risk of performance bias for blinding because the reported outcomes were objective and unlikely to be influenced by lack of blinding. However, the Selke trial [16] did not give sufficient information to permit judgment of whether or not detection bias was present. The Silveira trial [26] was judged to have a high risk of performance and detection bias due to lack of blinding for participants, personnel and outcome assessment respectively for all study outcomes. All trials have a low risk of attrition bias as they did not have differential or large numbers of losses to follow-up across the intervention arms. Selective reporting bias was judged to be low for the study by Jaffar et al [25] and unclear for the Selke trial [16] because the trial protocol was not available. The Silveira trial [26] was judged as having unclear risk of reporting bias

For peer review only

For peer review only - http://bmjopen.bmj.com/site/about/guidelines.xhtml

due to insufficient information and as having a high risk of other biases due to inadequate sample size. Figure 2 provides a graphical summary of the risk of bias assessments.

Mortality

There were 6 reported deaths across all three trials. When pooled, we found there was very low certainty regarding the effect of the task shifting from pharmacy to non-pharmacy personnel on death (RR 1.86; 95% CI: 0.44 to 7.95, figure 3), with no significant heterogeneity ($Chi^2 = 3.34$; df = 2; p = 0.19; $I^2 = 40\%$). We downgraded the quality of the evidence for indirectness because two of the three trials (Selke [16] and Jaffar [25]) compared complex interventions that included, but were not limited to, pharmacy and non-pharmacy personnel as shown in Table 5. There were few deaths and the effect estimate was imprecise, with very wide confidence intervals ranging from appreciable benefit to substantial harm.

Table 5: GRADE Summary of findings table for pharmacy versus non-pharmacy personnel for dispensing antiretroviral therapy

Population: People living with HIV
Setting: Brazil, Kenya, and Uganda (one study per country)
Intervention: Non-pharmacy personnel for dispensing antiretroviral therapy
Control: Pharmacy personnel for dispensing antiretroviral therapy

Outcomes (Mean follow-up: 12 months)	Pharmacy personnel	Non-pharmacy personnel	Relative effect (95% CI)	No. of Participants (studies)	Quality of evidence (GRADE)
**Mortality**	**76 per 1000**	**141 per 1000** (33 to 602)	**RR 1.86** (0.44 to 7.95)	1993 (three studies)	⊕⊕⊕⊖ **very low**[1,2]
**Virological failure**	**131 per 1000**	**120 per 1000** (95 to 150)	**RR 0.92** (0.73 to 1.15)	1993 (three studies)	⊕⊕⊖⊖ **low**[1,3]
**Loss to follow up**	**28 per 1000**	**31 per 1000** (19 to 53)	**RR 1.13** (0.68 to 1.91)	1993 (three studies)	⊕⊕⊖⊖ **low**[1,3]

CI: Confidence interval; **RR:** Risk ratio;

GRADE Working Group grades of evidence
High certainty: Further research is very unlikely to change our confidence in the estimate of effect.
Moderate certainty: Further research is likely to have an important impact on our confidence in the

For peer review only - http://bmjopen.bmj.com/site/about/guidelines.xhtml

estimate of effect and may change the estimate.

Low certainty: Further research is very likely to have an important impact on our confidence in the estimate of effect and is likely to change the estimate.

Very low certainty: We are very uncertain about the estimate.

[1] We downgraded by one for serious indirectness: Two trials compared complex interventions that included, but were not merely limited to, pharmacy and non-pharmacy personnel.

[2] We downgraded by two for serious imprecision: There are few events and the effect estimates have wide confidence intervals, ranging from appreciable benefit to harm.

[3] We downgraded by one for serious imprecision: There are few events and the effect estimates have wide confidence intervals, ranging from appreciable benefit to harm.

For peer review only

Virological response

We found low certainty evidence that there may be no differences in virological failure between the group cared for by non-pharmacy personnel and that cared for by pharmacy personnel (RR 0.92; 95% CI: 0.73 to 1.15, figure 4), with no heterogeneity (Chi^2 = 0.24; df = 2; p=0.89; I^2 = 0%). The quality of the evidence for this outcome was low due to serious indirectness and imprecision as shown in Table 5.

Loss to follow up

We found low certainty evidence that there may be no difference in loss to follow up between the groups (RR 1.13; 95% CI: 0.68 to 1.91), with no heterogeneity (Chi^2 = 0.54; df = 2; p=0.76; I^2 = 0%), figure 5). The quality of the evidence was low due to serious indirectness and imprecision as shown in Table 5.

Clinic visits

Selke [16] reported significantly more clinic visits in the pharmacy compared to the non-pharmacy personnel group (mean visits 12.6 versus 6.4), p<0.001. Although the non-pharmacy personnel group was found to have fewer clinic visits, the authors observed that this group attended 64% more clinic visits than originally scheduled. The study by Jaffar [25] found a high frequency of outpatient attendance (15,242 visits) in the pharmacy compared to the non-pharmacy personnel group (6,691 visits). However, they found similar distribution of new diagnoses between groups where more than 50% were infectious and parasitic infections. The trial by Silveira [26] did not report on clinic visits.

Incidence of opportunistic infections

For peer review only - http://bmjopen.bmj.com/site/about/guidelines.xhtml

Selke[16] trial reported an incidence of 13.6 HIV opportunistic infections per 100 person-years in the non-pharmacy personnel group compared to 19.8 HIV opportunistic infections per 100 person-years in the pharmacy personnel group.

Adherence

All trials reported similar high-level adherence using different measures.

Cost

In the trial by Jaffar [25], a societal perspective economical analysis showed a higher mean cost per patient per year in the pharmacy personnel group ($838) for health services (staff, transport, drugs, laboratory and clinical services, sensitisation, training and workshops, utilities, supervision and overhead capital) compared to non-pharmacy personnel group ($793). Similarly, total per patient costs to access care per year were higher in the pharmacy personnel group ($54) compared to non-pharmacy personnel group ($18). Costs to access care included transport, lunch, childcare and lost work time. The outcome cost was not pre-specified in the protocol, however, this provides additional data that may be relevant for decision makers and was therefore included for consideration.

Acceptability to pharmacy personnel, non-pharmacy personnel and patients and harm, including error rates

These outcomes were not reported in the included trials.

Discussion

Summary of main results

Two cluster randomised clinical trials and one non-blinded randomised controlled trial were included in this review of effects of shifting responsibility from pharmacy

For peer review only - http://bmjopen.bmj.com/site/about/guidelines.xhtml

to non-pharmacy personnel for adherence assessment and dispensing antiretroviral drugs to HIV-infected patients.

In our meta-analysis, we found very low certainty evidence for the outcome mortality. We are therefore not certain whether the intervention may impact on mortality or not. We found low certainty evidence that there may be no difference in virological response, and loss to follow up between non-pharmacy personnel and pharmacy personnel distributing ART to patients. In addition, we did not find significant differences in adherence to treatment. Selke et al [16] reports that the non-pharmacy personnel group had significantly fewer all-cause sick visits to the clinic compared to the pharmacy personnel group. Although the main difference between the Selke [16] and Jaffar [25] trials was the level of education of the non-pharmacy personnel, it did not seem to affect their overall performance [16 25].

Additionally, the Jaffar trial [25] found the non-pharmacy dispensing strategy to be cost–effective and cheaper to run than the pharmacy dispensing strategy by almost 45 US dollars per patient per annum and this accounted for only 6% of the total cost of healthcare service expenditure for the intervention group [25]. The patients who were accessing their medications and other healthcare services from pharmacy personnel incurred more costs in terms of transportation, lunch, childcare costs and lost work time. In the first year of the study, the pharmacy personnel group incurred double the cost of healthcare services per patient per annum compared to the non-pharmacy personnel group. This is a challenge in most poor settings in Africa where many cannot afford basic necessities and this further impedes their access to treatment [25]. Lack of or inadequate financial resources are major factors in late presentation to health facilities, poor access to HIV care and support, and low retention in care after initiation of ART [32 33]. Although health services are provided for free in most public

For peer review only - http://bmjopen.bmj.com/site/about/guidelines.xhtml

institutions, the cost of accessing the services may serve as a hindrance to achieving optimal care and support for HIV-infected patients [32]. The non-pharmacy personnel dispensing ART may reduce the cost of services for both patients and governments with potentially favourable clinical outcomes.

Although issues related to drug-drug interaction, medication errors, pre-screening for ART and some special cases of adherence to therapy may be beyond the capacity of non-pharmacy personnel, targeted pharmacy support to non-pharmacy personnel could offer benefits to patients having challenges with their medications especially those who are taking concomitant medications with ART [34].

This review and meta-analysis has shown that the use of non-pharmacy personnel comprising either lay people or other health professionals such as nurses who are given well-tailored and comprehensive short trainings with refreshers may be able to support ART dispensing systems for HIV-infected patients, however, the low certainty evidence implies that additional trial evidence may change our results. The use of non-pharmacy personnel such as lay people for dispensing ART and monitoring patients' health would enable identification of psychosocial features that might be overlooked by physicians, nurses and pharmacists at the facility. Some of these psychosocial problems such as gender-based violence, food insecurity and alcohol abuse negatively impact on adherence and retention in care [16]. In the case of community-based non-pharmacy personnel, they could also serve as a link between the patients and other healthcare workers and facilitate smooth communication between the patients and the pharmacists on issues such as changes in regimen and dosage of drugs [16 34].

For peer review only

Overall completeness and applicability of evidence

Although it may not be possible to rule out publication and language biases in systematic reviews, our electronic search was not restricted to language, setting or publication status.

We identified three studies for inclusion with small sample sizes and event rates. The trials did not specifically pose the question of task shifting to non-pharmacy personnel and included this approach within complex interventions. As such, this provides indirect evidence where other aspects of the intervention may have resulted in the outcomes reported. The trials did not include some of the outcomes of interest for decision-making such as acceptability to participants and feasibility of the intervention. These limitations might have affected our conclusions about the findings from these trials.

Certainty of evidence

We assessed the certainty of evidence using the GRADE approach [35]. Evidence from this review should be applied with caution considering its very low and low quality. We are therefore not confident enough to state that the estimates lie close to the true value and it is likely that further studies would contribute to the body of knowledge to inform these results.

Conclusions

There is low certainty evidence that loss-to-follow up and virological outcomes may be the same whether pharmacy or non-pharmacy personnel provide services to people living with HIV on ART in resource-limited settings. This is particularly when accompanied by continued capacity building and supervision by trained pharmacy personnel. However, there is very low certainty evidence regarding the mortality

For peer review only

For peer review only - http://bmjopen.bmj.com/site/about/guidelines.xhtml

outcome where further evidence will impact the result. The use of non-pharmacy personnel including lay people in dispensing and distributing ART may be cost-effective for the patient and health system. Strong referral systems are crucial in task-shifting of dispensing responsibilities to non-pharmacy personnel to support patients that require advanced medical attention. Due to the critical shortage of human personnel in the health sector, most HIV programmes engaged other cadres of health personnel other than doctors to prescribe and dispense ART at the onset of their programme implementation. This might have contributed to scarcity of studies on the use of pharmacy personnel for dispensing and distributing ART. More trials, with adequate sample sizes to detect clinically relevant health benefits are therefore needed to investigate task-shifting of dispensing and distributing ART from pharmacy to non-pharmacy personnel. Programmes considering implementation of task-shifting should do so with inclusion of close monitoring and evaluation for health and process outcomes.

Author contributions

EN, TK, and CSW conceived the idea; NMM, TK and CSW drafted the review protocol; NMM, OA, TK and CSW conducted screening, selected the studies, extracted data, performed analyses and drafted the manuscript. All authors reviewed and approved the manuscript.

Acknowledgements

Funding for preparing this review was provided by the Department of HIV/AIDS at the WHO Headquarters in Geneva, Switzerland through a grant from University of California, San Francisco. The authors would like to thank George Rutherford and

For peer review only

Hacsi Horvath (Global Health Sciences, University of California, San Francisco, USA) for support and specific assistance in developing the search strategy for electronic databases. Neither the authors' institutions nor the funders played a role in preparing the manuscript, and the views expressed therein are solely those of the authors. We would like to thank Joy Oliver from Cochrane South Africa, South African Medical Research Council for assistance with the updated search. Both Charles Wiysonge and Tamara Kredo are partly supported by the Effective Health Care Research Consortium. This Consortium is funded by the UK government for the benefit of developing countries (Grant: 5242). The views expressed in this publication do not necessarily reflect UK government policy.

Competing interests

None declared

Data sharing Statement

Additional unpublished data is not available

References

1. UNAIDS. How AIDS changed everything - MDG 6: 15 years, 15 lessions of hope from the AIDS response. http://wwwunaidsorg/en/resources/presscentre/pressreleaseandstatementarchive/2015/july/20150714 PR MDG6report 2015;**accessed 22nd September 2015**.

For peer review only

For peer review only - http://bmjopen.bmj.com/site/about/guidelines.xhtml

2. Palella FJ DK, Moorman AC, Loveless MO, et al. Declining morbidity and mortality among patients with advanced human immunodeficiency virus infection. New England Journal of Medicine 1998;**338**(13):853-60.

3. Anglemyer A RG, Horvath T, Baggaley RC, Egger M, Siegfried N. Antiretroviral therapy for prevention of HIV transmission in HIV-discordant couples. Cochrane Database Syst Rev 2013;**4**(CD009153).

4. Ford N, Calmy, A., Mills, E,J. The first decade of antiretroviral therapy in Africa. Globalization and Health 2011;**7**(33).

5. King RC, Fomundam, H.N. Remodeling pharmaceutical care in sub-Saharan Africa (SSA) amidst human resources challenges and the HIV/AIDS pandemic. Int J Health Plann Manage 2010;**25**(1):30-48.

6. SAPC. Pharmacy Human Resources data. http://wwwhealthlingorgza/healthstats/283/data;accessed **15th August, 2015**.

7. Tseng A, Foisy, M., Hughes, C.A., et, al. Role of pharmacists in caring for patients with HIV/AIDS: Clinical Practice Guidelines. CJHP 2012;**65**(2):125-45.

8. Saberi P, Dong, B.J., Johnson, M.O., Greenblatt, R.M., Cocohoba, J.M. The impact of HIV clinical pharmacists on HIV treatment outcomes: systematic review. Patient Prefer Adherence 2012;**6**:297-322.

9. Kitahata MM, Koepsell, T.D., Deyo, R.A., et, al. Physicians experience with the acquired immunodeficiency syndrome as a factor in patients' survival. N Engl J Med 1996;**334**(11):701-6.

10. Landon BE, Wilson, I..B., Malness, K., et, al. Physician specialization and the quality of care for human immunodeficiency virus infection. Arch Intern Med 2005;**165**(10):1133-9.

11. Kredo T AF, Bateganya M, Pienaar ED. Task shifting from doctors to non-doctors for initiation and maintenance of antiretroviral therapy. Cochrane Database of Systematic Reviews 2014;**CD007331**(7).

12. MSH. *Managing Drug Supply*. Second Edition ed. 14 Oakwood Avenue, West Hartford, Connecticut, 06119-227 USA: Kumarian Press Inc, 1997.

13. WHO. Management of Drugs at health Centre Level - Training Manual. WHO Regional Office for Africa 2004;http://www.who.int/medicinedocs/en/d/js791e.

14. Decroo T, Telfer, B., Biot M, et al. Distribution of antiretroviral treatment through self-forming groups of patients in Tete province, Mozambique. J Acquir Immune Defic Syndr 2011;**56**:39-44.

15. Kipp W, KondeLule, J., Rubaale,T., et al. Comparing antiretroviral treatment outcomes between a prospective community-based and hospital-based cohort of HIV patients in rural Uganda. BMC Int Health Hum Rights 2011;**11Suppl**(2):S12.

16. Selke H, Kimaiyo, S., Sidle, J.E., et, al. Task-shifting of antiretroviral delivery from health care workers to persons living with HIV/AIDS: clinical outcomes of a community-based program in Kenya. J Acquir Immune Defic Syndr 2010;**55: 483-90.**

17. Callaghan M, Ford, N., Schnider, H. A systematic review of task-shifting for HIV treatment and care in Africa. Human Resources for health 2010;**8**(8).

For peer review only - http://bmjopen.bmj.com/site/about/guidelines.xhtml

18. Laurant M RD, Hermens R, Braspenning J, Grol R, Sibbald B. Substitution of doctors by nurses in primary care. Cochrane Database of Systematic Reviews 2005;**CD001271**.
19. Mbeye NM, Kredo, T., Wiysonge, C.S. The effects of shifting tasks from pharmacy to non-pharmacy personnel for providing antiretroviral therapy to people living with HIV: a systematic review protocol. BMJ Open 2016;**6:e008195**.
20. Higgins JPT, Green, S. (eds). Cochrane Handbook for Systematic Reviews of Interventions. Wiley-Blackwell 2008.
21. Egger M DSG, Schneider M, Minder C. Bias in meta-analysis detected by a simple, graphical test. BMJ 1997;**315**(7109):629-34.
22. (RevMan) RM. Computer program. The Nordic Cochrane Centre, The Cochrane Collaboration, Copenhagen 2015;**Version 5.3**.
23. Guyatt GH OA, Santesso N, Helfand M, Vist G, Kunz R, et al. GRADE guidelines: 12. Preparing Summary of Findings tables-binary outcomes. Journal of Clinical Epidemiology 2013;**66**(2):158-72.
24. Guyatt GH TK, Oxman AD, Walter SD, Patrick D, Furukawa TA, et al. GRADE guidelines: 13. Preparing Summary of Findings tables and evidence profiles-continuous outcomes. Journal of Clinical Epidemiology 2013;**66**(2):173-83.
25. Jaffar S, Amuron B., Foster S., et, al. Rates of virologic failure in patients treated in a home-based versus a facility-based HIV-care model in Jinja, southeast Uganda: a cluster-randomised equivalence trial. Lancet 2009;**DOI:10.1016/S1040-6736(09)61674-3.**
26. Silveira MPT, Guttier, M.C., Page, K., Moreira, L.B. Randomized Controlled Trial to Evaluate the Impact of Pharmaceutical Care on Therapeutic Success in HIV-Infected Patients in Southern Brazil. AIDS Behav 2014;**18**:S75-S84.
27. March K MM, Louie SG. Effects of pharmacists' interventions on patient outcomes in an HIV primary care clinic. American Journal of Health-System Pharmacy 2007;**64**:2574-78.
28. Chang LW KJ, Nakigozi G, Ssempijja V, Packer AH, Serwadda D, Quinn TC, Gray RH, Bollinger RC, Reynolds SJ. Effect of peer health workers on AIDS care in Rakai, Uganda: A cluster-randomized trial. PLoS ONE 2010:; **5** (6).
29. Kiweewa FM WD, Nakibuuka J, Mubiru M, Bagenda D, Musoke P, Fowler MG, Antelman G. Noninferiority of a task-shifting hiv care and treatment model using peer counselors and nurses among ugandan women initiated on ART: Evidence from a randomized trial. Journal of Acquired Immune Deficiency Syndromes 2013;**63** (4):e125-e32.
30. Hansudewechakul R NT, Katana A, Faikratok W, Lolekha R, Thainuea V, McConnell MS. Successful clinical outcomes following decentralization of tertiary paediatric HIV care to a community-based paediatric antiretroviral treatment network, Chiangrai, Thailand. Journal of the International AIDS Society 2012;**15 (2)**
31. Henderson KC HJ, Johnson SC, Valuck RJ, Kiser JJ. Assessing the effectiveness of pharmacy-based adherence interventions on antiretroviral adherence in persons with HIV. IDS Patient Care and STDs **25**(4):221-28.
32. Hardon A, Akurut, D., Comoro, C., et al. Hunger, waiting time and transport costs: time to confront challenges to ART adherence in Africa. AIDS Care 2007;**19: 658–65.**

For peer review only

For peer review only - http://bmjopen.bmj.com/site/about/guidelines.xhtml

33. Rosen S, Fox, MP., Gill, C.J. Patient retention in antiretroviral therapy programs in sub-Saharan Africa: a systematic review. PLoS Med 2007;**4: e298.**
34. Seden K, . Bradley, M., Miller, AR., Beadsworth, M.B., Khoo, S.H. The clinical utility of HIV outpatient pharmacist prescreening to reduce medication error and assess adherence. Int J STD AIDS 2013:;**24 (3);237-41.**
35. Guyatt GOA, Akl, E.A., Kunz, R., Vist, G., Brozek, J., Norris, S,. Falck-Ytter, Y., Glasziou, P., DeBeer, H., Jaeschke, R,. Rind, D., Meerpohl, J., Dahm, P., Schünemann, H.J. GRADE guidelines: 1. Introduction-GRADE evidence profiles and summary of findings tables. Journal of Clinical Epidemiology 2011;**64(4): 383-94**.

List of figures

Fig 1: Flow diagram showing the search and selection of studies

Fig 2: Summary of risk of bias in included studies

Fig 3: Effect of shifting dispensing of ART from pharmacy to non-pharmacy personnel on mortality

Fig 4: Effect of shifting dispensing of ART from pharmacy to non-pharmacy personnel on virological failure

Fig 5: Effect of shifting dispensing of ART from pharmacy to non-pharmacy personnel on loss to follow up

Supporting Information

S1. Task-shifting review protocol

S2. PRISMA Checklist

For peer review only

For peer review only - http://bmjopen.bmj.com/site/about/guidelines.xhtml

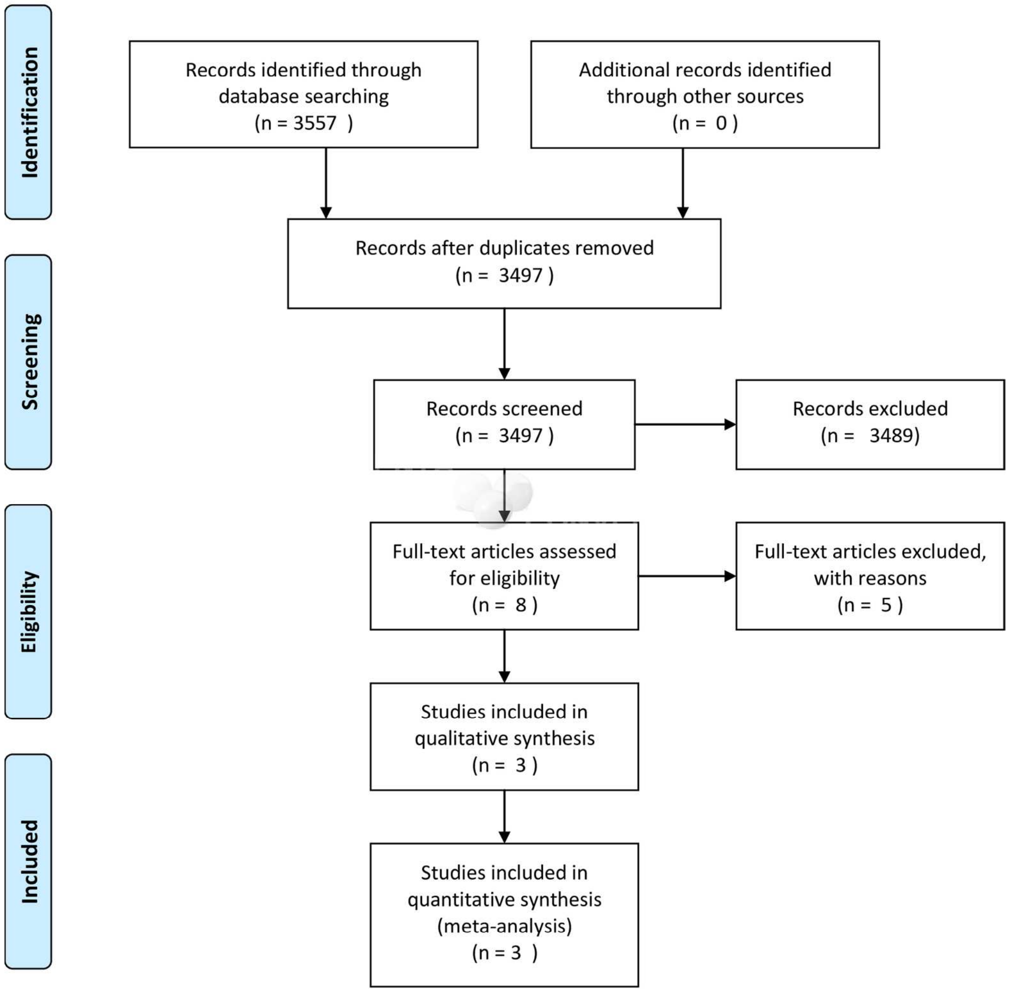

Flow diagram showing the search and selection of studies

168x179mm (300 x 300 DPI)

Domain	Trial		
	Jaffar 2009	Selke 2010	Silveira 2013
Random sequence generation (selection bias)	+	+	+
Allocation concealment (selection bias)	+	+	?
Blinding of participants and personnel (performance bias)	+	+	–
Blinding of outcome assessment (detection bias)	+	?	–
Incomplete outcome data (attrition bias)	+	+	+
Selective reporting (reporting bias)	+	?	?
Other bias	+	?	–

+ Low risk; – High risk; ? Unclear risk.

Summary of risk of bias in included studies

144x87mm (300 x 300 DPI)

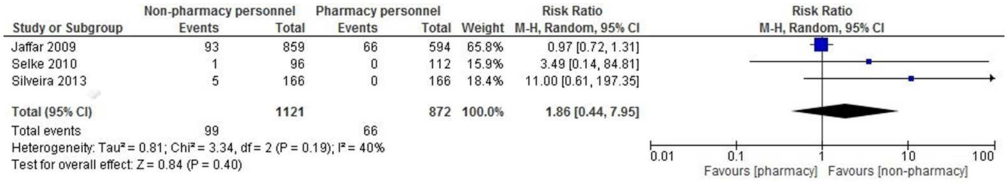

Effect of shifting dispensing of ART from pharmacy to non-pharmacy personnel on mortality

80x14mm (300 x 300 DPI)

For peer review only

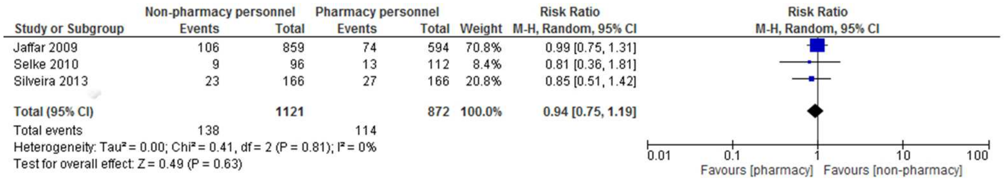

Effect of shifting dispensing of ART from pharmacy to non-pharmacy personnel on virological failure

80x14mm (300 x 300 DPI)

For peer review only

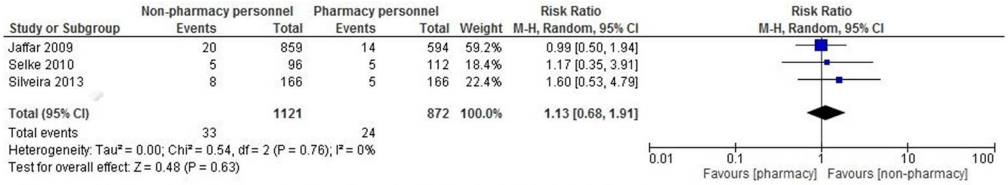

Effect of shifting dispensing of ART from pharmacy to non-pharmacy personnel on loss to follow up

79x15mm (300 x 300 DPI)

For peer review only

Downloaded from http://bmjopen.bmj.com/ on September 16, 2016 - Published by group.bmj.com

Open Access Protocol

BMJ Open

The effects of shifting tasks from pharmacy to non-pharmacy personnel for providing antiretroviral therapy to people living with HIV: a systematic review protocol

Nyanyiwe M Mbeye,[1,2] Tamara Kredo,[1] Charles S Wiysonge[1,2]

To cite: Mbeye NM, Kredo T, Wiysonge CS. The effects of shifting tasks from pharmacy to non-pharmacy personnel for providing antiretroviral therapy to people living with HIV: a systematic review protocol. *BMJ Open* 2016;**6**: e008195. doi:10.1136/bmjopen-2015-008195

▸ Prepublication history for this paper is available online. To view these files please visit the journal online (http://dx.doi.org/10.1136/bmjopen-2015-008195).

Received 15 March 2015
Revised 16 November 2015
Accepted 17 November 2015

[1]Cochrane South Africa, South African Medical Research Council, Tygerberg, South Africa
[2]Faculty of Medicine and Health Sciences, Centre for Evidence-based Health Care, Stellenbosch University, Cape Town, South Africa

Correspondence to
Dr Nyanyiwe M Mbeye; nyanyiwembeye@gmail.com

ABSTRACT

Introduction: Shifting selected antiretroviral therapy (ART) tasks from specialised healthcare workers to those with shorter or less formal training has been implemented in resource-limited settings to alleviate critical shortages of human resources for health. However, the specifics of shifting ART dispensing from pharmacy to non-pharmacy personnel have not been addressed in a systematic review, although this can potentially increase access to ART. We will assess the effects of shifting dispensing and distribution of ART and adherence assessment from pharmacy to non-pharmacy personnel in low and middle-income countries.

Methods and analysis: We will search PubMed, CENTRAL, EMBASE, WHO Global Health Library and relevant grey literature for eligible controlled trials. Two authors will screen the search output, select eligible studies, assess risk of bias and extract data from included studies, resolving discrepancies by discussion and consensus. We will perform meta-analysis using both fixed and random effects models, investigate clinical and statistical heterogeneity, and assess our confidence in the overall evidence using standard Cochrane methods, including GRADE.

Ethics and dissemination: Only secondary data will be included in this review and ethics approval is not required. We will disseminate the review findings in various scientific fora, including peer-reviewed journals. The findings may help to inform policy makers in defining the scope of work of healthcare workers, and global recommendations for shifting the dispensing and distribution of ART from pharmacy to non-pharmacy personnel.

Trial registration number: CRD42015017034.

Strengths and limitations of this study

- To our knowledge, this is the first published protocol of a systematic review that will investigate the effects of task shifting from pharmacy to non-pharmacy personnel for dispensing or distributing antiretroviral therapy to patients living with HIV.
- The protocol was written according to the PRISMA-P (Preferred Reporting Items for Systematic Review and Meta-Analysis Protocols) recommendations.
- The review findings may help to inform antiretroviral therapy guidelines by the WHO.
- The possible weakness of the planned review would be the limitations of included studies, for example, high risk of bias and heterogeneity of settings, designs and effects.

INTRODUCTION

Description of the condition

By March 2015, 15 million (40.7%) of the estimated 36.9 million people living with HIV (PLHIV) globally were receiving antiretroviral therapy (ART).[1] Combination ART is effective for reducing HIV related morbidity and mortality as well as preventing HIV transmission.[2] Initiating ART early in the course of HIV infection has been associated with better health outcomes, both at patient and population levels.[3 4] Scale-up of ART in low and middle income countries has averted more than 5 million deaths; however, bottlenecks preventing universal access to ART still exist. One challenge is the critical shortage of human resources for health (HRH), including for the delivery of essential HIV related pharmacy services.

The WHO recommends a minimum of one pharmacist per 2300 population, but most countries in low-resource settings such as sub-Saharan Africa have not yet met this target.[5] In addition to the absolute shortage, it is likely that there is an uneven distribution of pharmacists in such settings, as is the case with other specialist healthcare workers who

For peer review only - http://bmjopen.bmj.com/site/about/guidelines.xhtml

Downloaded from http://bmjopen.bmj.com/ on September 16, 2016 - Published by group.bmj.com

tend to concentrate in urban areas and the private sector, further aggravating the HRH shortage.[6] For instance in South Africa, which is home to the largest number of PLHIV in any country in the world, in 2010 only 24% of registered pharmacists worked in the public sector where 80% of the population received care.[7]

Description of the intervention

Studies and programme reports indicate that involvement of pharmacy personnel in HIV care results in improved patient outcomes. For instance, in the USA, the use of a multidisciplinary team approach with pharmacists assuming a central role in ART initiation, dispensing and adherence counselling improved treatment outcomes such as viral load, patient retention and medication adherence.[8]

The work of pharmacists includes supply management, dispensing and distributing medications, promoting adherence, identifying and preventing potential medication-related issues, and monitoring and reporting adverse events. In some settings, programmes have implemented alternative models of pharmacy services that shift selected tasks from pharmacy to non-pharmacy personnel. Such alternative models could potentially increase the number of health workers involved in ART distribution, adherence counselling and patient education, free more time for pharmacy personnel, support the integration of ART in primary care settings, minimise the number of facility visits for ART collection, and reduce pharmacy queue waiting times for patients.[9]

However, the specifics of shifting ART related tasks from pharmacy to non-pharmacy personnel have not yet been addressed in a systematic review. We therefore plan to synthesise the evidence for task shifting in pharmacy services, where non-pharmacy personnel undertake ART dispensing and distribution and medication adherence counselling. For this systematic review, pharmacy personnel will include both pharmacists and pharmacy technicians. Pharmacy technicians constitute an important part of the pharmacy workforce in low and middle-income countries: a survey of 26 low and middle-income countries in 2011 revealed that pharmacy technicians constitute 10% (Nigeria) to 70% (Pakistan) of the pharmacy workforce.[10]

How the intervention might work

Within the last decade, several high HIV burden countries adopted task shifting strategies where nurses and non-physician clinicians initiate and maintain ART.[11] Although this has undeniably expanded access to ART, it is also increasingly essential that long facility waiting times and frequent facility visits to collect ART are addressed to alleviate the burden of care, both for patients and healthcare providers.[12 13]

Recent studies in Uganda, Kenya and Mozambique have shown positive outcomes when non-health professionals (lay people) delivered ART at the community level.[14] In Mozambique the use of PLHIV for distributing ART, monitoring adherence, reporting outcomes and referring sick patients to health facilities yielded a retention rate of 97.5% among stable patients on ART.[14] In a cluster randomised trial in Uganda, the use of trained community health workers produced comparable results with facility-based ART programme in terms of patient retention, viral load suppression and mortality rate.[15] Similar findings were also obtained in Kenya and Uganda when lay providers were engaged in ART delivery.[15 16]

Task shifting has therefore been seen as an achievable solution to the critical human resource shortages for scale-up of ART.[17] While it is imperative to increase the rate of recruitment and training of health workers as well as improve working conditions to reduce attrition and emigration, the HIV pandemic requires a more urgent measure to address the critical skills shortage.[18] Such measures may include shifting selected tasks (including dispensing and distributing ART and adherence counselling) from pharmacy to non-pharmacy personnel. The task shifting could free time for pharmacy personnel to focus on more technical functions such as supply management and pharmacovigilance.

Why this review is important

Previous systematic reviews of task shifting for increasing ART access focused on clinical services where nurses and non-clinician physicians provide care.[11] Dependence on and shortages of pharmacists are also key constraints on ART expansion, but the specifics of task shifting for ART dispensing or distribution from pharmacy to non-pharmacy personnel have not been reviewed systematically. We will systematically review the scientific literature and assess the efficacy and safety of task shifting models that use non-pharmacy personnel in dispensing or distributing ART and assessing adherence to treatment of HIV infection.

OBJECTIVE

The aim of this review is to evaluate the efficacy and safety of shifting dispensing and distribution of ART as well as assessment of adherence from pharmacy to non-pharmacy personnel.

METHODS

This review protocol has been registered in the PROSPERO International Prospective Register of Systematic Reviews (http://www.crd.york.ac.uk/PROSPERO), registration number CRD42015017034.

Criteria for considering studies for this review

Types of studies

We will include randomised controlled trials (RCTs) and non-RCTs, irrespective of whether allocation to interventions occurred at the individual or cluster level.

Types of participants

Participants will be PLHIV receiving ART.

For peer review only - http://bmjopen.bmj.com/site/about/guidelines.xhtml

Downloaded from http://bmjopen.bmj.com/ on September 16, 2016 - Published by group.bmj.com

Types of interventions

We will include studies that evaluate the shifting of selected tasks from pharmacy personnel to non-pharmacy personnel. The selected tasks include dispensing and distribution of ART and adherence assessment. Pharmacy personnel will include both pharmacists and pharmacy technicians. Non-pharmacy personnel may include (but are not limited to) nurses, non-physician clinicians, and lay providers such as patient peer groups, community volunteers, PLHIV and community health committees.

Types of outcome measures

Primary outcomes

The primary outcome for this review is risk of death.

Secondary outcomes

Our secondary outcome measures include:

- Virological suppression
- Number of all-cause sick visits made to the health facility, including for adverse events
- Loss to follow-up
- Adherence to ART (as measured within the study, eg, pill counts, recall methods, digital methods)
- Acceptability to pharmacy personnel, non-pharmacy personnel and patients
- Harm, including error rates.

Search methods for identification of studies

We will perform a comprehensive and exhaustive search of electronic databases and conference proceedings in an attempt to identify all relevant studies available by the search date, regardless of language of publication or publication status (published, unpublished, in press or in progress).

Databases of peer-reviewed literature

We will search the following electronic databases, from 1 January 1996 to the search date:

- Cochrane Central Register of Controlled Trials (CENTRAL)
- Excerpta Medica Database (EMBASE)
- PubMed
- ISI Web of Science (Science Citation index)
- WHO Global Health Library, which includes references from AIM (AFRO), LILACS (AMRO/PAHO), IMEMR (EMRO), IMSEAR (SEARO) and WPRIM (WPRO).

Along with appropriate Medical Subject Heading (MeSH) terms and relevant keywords, we will use the Cochrane Highly Sensitive Search Strategy for identifying reports of randomised controlled trials in MEDLINE,[19] and the Cochrane validated strategies for identifying references relevant to HIV infection and AIDS. To identify other study designs, the RCT string will be omitted. The search strategy will be iterative in that references of included studies will be searched for additional references. See table 1 for our provisional search strategy for electronic databases.

Conference databases

We will search conference abstract archives on the web sites of the Conference on Retroviruses and Opportunistic Infections (CROI), the International AIDS Conference (IAC) and the International AIDS Society Conference on HIV Pathogenesis, Treatment and Prevention (IAS), for all available abstracts presented at these conferences from 1996 to the search date.

Searching other resources

We will also search the references of relevant articles as well as the WHO International Clinical Trials Registry Platform (ICTRP) and Clinicaltrials.gov. We will contact relevant experts or organisations who may be aware of additional studies in this field.

Data collection and analysis

We will base the methodology for data collection and analysis on the guidance provided in the Cochrane Handbook of Systematic Reviews of Interventions.[19]

Selection of studies for inclusion

Two authors will read and assess the abstracts of identified publications for potentially eligible studies. We will obtain full text articles for all abstracts judged by at least one of the two authors, to be potentially eligible. Two authors will independently inspect these potentially eligible publications to establish the relevance of the article to the review according to the pre-specified criteria regarding study design, participants, interventions and outcome measures.

Data extraction and management

Two authors will independently extract data into a pre-piloted data extraction form. The following characteristics will be extracted from each included study:

Study details: Complete citations of publications associated with the study, start and end dates, location, study design characteristics, type of facility involved, investigators, funding sources, recruitment, method of randomisation, sequence generation, method of allocation concealment, blinding of participants and personnel, blinding of outcome assessment, length of follow-up, losses to follow-up, withdrawals or drop-outs and other relevant details.

Details of the intervention: training of the cadre of health workers who were dispensing or distributing ART, what training or other support or supervision they received and other relevant details.

Details of participants: Trial inclusion and exclusion criteria, numbers of participants entering the trial, sex, clinical staging, CD4 count and other pertinent details.

Outcome details: Definitions of outcomes, details of how outcomes were assessed, numerators and denominators associated with each outcome, completeness of outcome data, effect estimates reported and other relevant outcome information.

For peer review only

For peer review only - http://bmjopen.bmj.com/site/about/guidelines.xhtml

Downloaded from http://bmjopen.bmj.com/ on September 16, 2016 - Published by group.bmj.com

Table 1 Proposed search strategy for electronic databases

ID	Search terms
PubMed	
#1	(task*[tiab] OR task-shifting[tiab] OR referr*[tiab] OR referral and consultation[mh] OR role*[tiab]) AND (health personnel [mh] OR doctor[tiab] OR doctors[tiab] OR clinician[tiab] OR clinicians[tiab] OR physician[tiab] OR physicians[tiab] OR "healthcare provider"[tiab] OR "healthcare providers"[tiab] OR "health care provider"[tiab] OR "health care providers"[tiab] OR pharmac*[tiab] OR apothecar*[tiab] OR chemist*[tiab] OR dispensar*[tiab])
#2	randomized controlled trial[pt] OR controlled clinical trial[pt] OR randomized controlled trials[MeSH] OR random allocation[MeSH] OR double-blind method[MeSH] OR single-blind method[MeSH] OR clinical trial[pt] OR clinical trials [MeSH] OR ("clinical trial"[tw]) OR ((singl*[tw] OR doubl*[tw] OR trebl*[tw] OR tripl*[tw]) AND (mask*[tw] OR blind*[tw])) OR random*[tw] OR research design[mh:noexp] OR prospective studies[MeSH] OR control*[tw] OR volunteer*[tw]) OR observational[tw] OR non-random*[tw] OR nonrandom*[tw] OR before after study[tw] OR time series[tw] OR cohort*[tw] OR cross-section*[tw] OR prospective*[tw] OR retrospective*[tw] OR research design[mh:noexp] OR follow-up studies [MeSH] OR longitud*[tw] OR evaluat*[tiab] OR pre-post[tw] OR (pre-test[tw] AND post-test[tw]) NOT (animals[MeSH] NOT human[MeSH])
#3	(HIV Infections[MeSH] OR HIV[MeSH] OR hiv[tiab] OR hiv-1[tiab] OR hiv-2*[tiab] OR hiv1[tiab] OR hiv2[tiab] OR hiv infect*[tiab] OR human immunodeficiency virus[tiab]OR human immune deficiency virus[tiab] OR human immuno-deficiency virus[tiab] OR human immune-deficiency virus[tiab] OR ((human immun*) AND(deficiency virus[tiab])) OR acquired immunodeficiency syndromes[tiab] OR acquired immune deficiency syndrome[tiab] OR acquired immuno-deficiency syndrome[tiab] OR acquired immune-deficiency syndrome[tiab] OR ((acquired immun*) AND (deficiency syndrome[tiab])) or "sexually transmitted diseases, viral"[mh]) OR HIV[tiab] OR HIV/AIDS[tiab] OR HIV-infected[tiab] OR HIV[title] OR HIV/AIDS[title] OR HIV-infected[title]
#4	(HAART[tiab] OR ART[tiab] OR cART[tiab] OR antiretroviral[tiab] OR anti-retroviral[tiab] OR anti-viral[tiab] OR antiviral [tiab] OR "Antiretroviral Therapy, Highly Active"[Mesh])
**#5**	#1 AND #2 AND #3 AND #4
Scopus	
	(HIV OR HIV/AIDS OR AIDS OR "HUMAN IMMUNODEFICIENCY" OR "ACQUIRED IMMUNODEFICIENCY)" AND TITLE-ABS-KEY (TASK-SHIFTING OR TASKSHIFTING OR (TASK* AND SHIFT*) OR TASK* OR (REFERR* AND (NURSE* OR PHARMAC*))) AND TITLE-ABS-KEY (ANTIRETROVIRAL OR ANTI-RETROVIRAL OR ART OR CART OR HAART) AND TITLE-ABS-KEY (RANDOM* OR RANDOMIZED OR RANDOMISED OR TRIAL OR COHORT* OR GROUP* OR COMPAR* OR OBSERVATIONAL OR PROSPECTIVE* OR RETROSPECTIVE* OR "SYSTEMATIC REVIEW" OR "META-ANALYSIS)"
Web of Science	
	(TS=(HIV OR HIV/AIDS OR AIDS OR "HUMAN IMMUNODEFICIENCY" OR "ACQUIRED IMMUNODEFICIENCY)" AND TS=(TASK-SHIFTING OR TASKSHIFTING OR (TASK* AND SHIFT*) OR TASK* OR (REFERR* AND (NURSE* OR PHARMAC*))) AND TS=(ANTIRETROVIRAL OR ANTI-RETROVIRAL OR ART OR CART OR HAART) AND TS= (RANDOM* OR RANDOMIZED OR RANDOMISED OR TRIAL OR COHORT* OR GROUP* OR COMPAR* OR OBSERVATIONAL OR PROSPECTIVE* OR RETROSPECTIVE* OR "SYSTEMATIC REVIEW" OR "META-ANALYSIS)")
OR	
	(TI=(HIV OR HIV/AIDS OR AIDS OR "HUMAN IMMUNODEFICIENCY" OR "ACQUIRED IMMUNODEFICIENCY)" AND TI=(TASK-SHIFTING OR TASKSHIFTING OR (TASK* AND SHIFT*) OR TASK* OR (REFERR* AND (NURSE* OR PHARMAC*))) AND TI=(ANTIRETROVIRAL OR ANTI-RETROVIRAL OR ART OR cART OR HAART) AND TI= (RANDOM* OR RANDOMIZED OR RANDOMISED OR TRIAL OR COHORT* OR GROUP* OR COMPAR* OR OBSERVATIONAL OR PROSPECTIVE* OR RETROSPECTIVE* OR "SYSTEMATIC REVIEW" OR "META-ANALYSIS)")
CENTRAL	
	HIV* OR HIV-1* OR HIV-2* OR HIV1 OR HIV2 OR HIV INFECT* OR HUMAN IMMUNODEFICIENCY VIRUS OR HUMAN IMMUNEDEFICIENCY VIRUS OR HUMAN IMMUNE-DEFICIENCY VIRUS OR HUMAN IMMUNO-DEFICIENCY VIRUS OR HUMAN IMMUN* DEFICIENCY VIRUS OR ACQUIRED IMMUNODEFICIENCY SYNDROME OR ACQUIRED IMMUNEDEFICIENCY SYNDROME OR ACQUIRED IMMUNO-DEFICIENCY SYNDROME OR ACQUIRED IMMUNE-DEFICIENCY SYNDROME OR ACQUIRED IMMUN* DEFICIENCY SYNDROME in Title, Abstract, Keywords and (TASK-SHIFTING OR TASKSHIFTING OR (TASK* AND SHIFT*) OR TASK* OR (REFERR* AND (NURSE* OR PHARMAC*))) in Title, Abstract, Keywords and ANTIRETROVIRAL OR ANTI-RETROVIRAL OR ART OR cART OR HAART in Title, Abstract, Keywords
WHO Global Health Library	
	(TASK-SHIFTING OR TASKSHIFTING OR (TASK* AND SHIFT*) OR TASK* OR (REFERR* AND (NURSE* OR PHARMAC*)) AND (HIV* OR human immunodeficiency) AND (antiretroviral OR anti-retroviral))) OR (HIV AND task-shifting) OR (HIV* AND task* AND shift*)

Assessment of risk of bias in included studies

We will assess the risk of bias in RCTs using the Cochrane risk of bias assessment tool for randomised studies.[19] For non-RCT studies, we will use the Cochrane Risk of Bias Assessment Tool for Non-Randomised Studies of Interventions (ACROBAT-NRSI).[20]

For peer review only - http://bmjopen.bmj.com/site/about/guidelines.xhtml

Downloaded from http://bmjopen.bmj.com/ on September 16, 2016 - Published by group.bmj.com

We will resolve any disagreements between the authors conducting duplicate independent screening of search outputs, assessments of study eligibility, extraction of data, and risk of bias assessment by discussion and consensus. Should this fail to resolve the differences, a third author will arbitrate.

Measures of effect

We will calculate and report risk ratios for dichotomous and time-to-event data and mean differences for continuous data with their 95% CIs.

Unit of analysis issues

The unit of analysis will be the individual study participant. Cluster-randomised trials will be included in meta-analyses only after adjustments are made for design effect. Design effects for cluster-randomised studies will be corrected by using standard procedures, using the formula: design effect=1+(m−1)r, where m is the average cluster size and r is the intra-cluster correlation coefficient.

Dealing with missing data

We will contact study authors if it is necessary to obtain data missing from published reports.

Assessment of heterogeneity

We will examine statistical heterogeneity between study results using the χ^2 test of homogeneity, with a significance α-level of 0.1. In addition, we will use the I^2 statistic to measure the amount of heterogeneity among the trials in each analysis. If we identify significant heterogeneity (ie, $p<0.1$), we will explore it by prespecified subgroup analysis. If heterogeneity persists, we will perform sensitivity analyses, report results separately and propose reasons for the observed heterogeneity.

Assessment of reporting biases

If any meta-analysis in our review includes 10 or more studies, we will assess the potential for publication bias using a funnel plot.[21] We will attempt to minimise the potential for publication bias through a comprehensive search of published and unpublished literature.

Data synthesis

We will conduct meta-analysis, if appropriate, using the Cochrane Review Manager software (RevMan [Computer program] The Nordic Cochrane Centre, *The Cochrane Collaboration, Copenhagen*, 2015; Version 5.3). If we find no significant statistical heterogeneity of effects, we will use the fixed effect method of meta-analysis. Otherwise, we will use the random effects model.

Subgroup analysis

In pooled results with significant statistical heterogeneity, we will explore the cause of the heterogeneity through subgroup analyses, with subgroups defined by type of intervention (eg, cadre of health provider), comparison group and region of study (eg, sub-Saharan Africa, Southeast Asia, etc).

Sensitivity analysis

We will conduct a sensitivity analysis to investigate the effect of excluding studies with high risk of bias, with a focus on bias introduced by inadequate allocation concealment, inadequate blinding of outcome assessment and substantial losses to follow-up.

Certainty of evidence

We will assess the certainty (or quality) of evidence using the Grading of Recommendations, Assessment, Development and Evaluation (GRADE) approach,[22] which defines the certainty of evidence for each outcome as "the extent of our confidence that the estimates of effect are correct".[19] The quality rating across studies has four levels: high, moderate, low and very low. Randomised trials are considered to be of high quality but can be downgraded for any of five reasons: risk of bias, indirectness of evidence, unexplained heterogeneity of effects, imprecision of effect estimates and high probability of publication bias. Similarly, observational studies are considered to be of low quality, but can be upgraded for any of three reasons. The quality level of a body of evidence can be increased if there is a large magnitude of effect, if all plausible confounding would reduce a demonstrated effect, and if there is a dose-response gradient.

Reporting of this review

The findings of this review will be presented in a number of ways. The study selection process will be summarised using a flow diagram, and if we identify 10 or more eligible studies, we will assess publication bias using funnel plots. Where appropriate, we will use risk of bias graphs, forest plots and GRADE summary of findings tables. The non-quantitative outcomes will be reported descriptively. We will provide tables of both included and excluded studies. We have prepared this protocol as recommended by the PRISMA-P (Preferred Reporting Items for Systematic Review and Meta-Analysis Protocols) guidelines[23] and will report the findings of the review as recommended by the PRISMA statement.[24]

ETHICS AND DISSEMINATION

Since systematic reviews do not directly involve human participants, they do not require ethical clearance.[25] We will provide the findings of this review to the WHO, with the hope that they may guide policy recommendations of this normative agent regarding the shifting of ART dispensing or distribution from pharmacy to non-pharmacy personnel. Although the majority of national programmes in low and middle-income countries have adopted task shifting in ART care at different levels,

For peer review only - http://bmjopen.bmj.com/site/about/guidelines.xhtml

Downloaded from http://bmjopen.bmj.com/ on September 16, 2016 - Published by group.bmj.com

there has been no global policy to guide the practice for task shifting from pharmacy to non-pharmacy personnel. We will also publish the findings of the systematic review in a peer-reviewed journal.

Twitter Follow Charles Wiysonge at @CharlesShey

Acknowledgements The authors acknowledge the Editor and the referees for critical and constructive comments on earlier versions of this manuscript. The authors would also like to acknowledge the various contributions of Eyerusalem Negussie.

Contributors NMM led the development of the protocol, wrote the first draft, coordinated and integrated comments from co-authors and approved the final version for publication. TK and CSW conceived the study, critically revised successive drafts of the manuscript, and approved the final version for publication. CSW is the guarantor of the manuscript.

Funding Funding for preparing this review was provided by the Department of HIV/AIDS at the WHO Headquarters in Geneva, Switzerland. The authors would like to thank Hacsi Horvath (Global Health Sciences, University of California, San Francisco, USA) for his assistance in developing the search strategy for electronic databases. Neither the authors' institutions nor the funder played a role in preparing the manuscript and the views expressed therein are solely those of the authors.

Competing interests None declared.

Provenance and peer review Not commissioned; externally peer reviewed.

Open Access This is an Open Access article distributed in accordance with the terms of the Creative Commons Attribution (CC BY 4.0) license, which permits others to distribute, remix, adapt and build upon this work, for commercial use, provided the original work is properly cited. See: http://creativecommons.org/licenses/by/4.0/

REFERENCES

1. UNAIDS. How AIDS changed everything—MDG 6: 15 years, 15 lessons of hope from the AIDS response. https//:www.unaids.org/en/resources/documents/2015/MDG6_15years-15lessonsfromtheAIDSresponse (accessed 22 Sep 2015).
2. Palella FJ, Delaney KM, Moorman AC, *et al*. Declining morbidity and mortality among patients with advanced human immunodeficiency virus infection. HIV Outpatient Study Investigators. *N Engl J Med* 1998;338:853–60.
3. Anglemyer A, Rutherford GW, Horvath T, *et al*. Antiretroviral therapy for prevention of HIV transmission in HIV-discordant couples. *Cochrane Database Syst Rev* 2013;4:CD009153.
4. Ford N, Calmy A, Mills EJ. The first decade of antiretroviral therapy in Africa. *Global Health* 2011;7:33.
5. King RC, Fomundam HN. Remodeling pharmaceutical care in sub-Saharan Africa (SSA) amidst human resources challenges and the HIV/AIDS pandemic. *Int J Health Plann Manage* 2010;25:30–48.
6. WHO. *Increasing access to health workers in remote and rural areas through improved retention: global policy recommendations*. Geneva, Switzerland: World Health Organisation, 2010.
7. SAPC. Pharmacy Human Resources data. https//:www.healthlink.org.za/healthstats/283/data (accessed 15 Aug 2015).
8. Saberi P, Dong BJ, Johnson MO, *et al*. The impact of HIV clinical pharmacists on HIV treatment outcomes: a systematic review. *Patient Prefer Adherence* 2012;6:297–322.
9. Tseng A, Foisy M, Hughes CA, *et al*. Role of pharmacist in caring for patients with HIV/AIDS: clinical practice guidelines. *Can J Hosp Pharm* 2012;65:125–45.
10. WHO. *The world of medicines situation 2011: pharmaceutical human resources*. Geneva, Switzerland: World Health Organisation, 2011.
11. Kredo T, Adeniyi FB, Bateganya M, *et al*. Task shifting from doctors to non-doctors for initiation and maintenance of antiretroviral therapy. *Cochrane Database Syst Rev* 2014;7:CD007331.
12. Kitahata MM, Koepsell TD, Deyo RA, *et al*. Physicians experience with the acquired immunodeficiency syndrome as a factor in patients' survival. *N Engl J Med* 1996;334:701–6.
13. Landon BE, Wilson IB, McInnes K, *et al*. Physician specialization and the quality of care for human immunodeficiency virus infection. *Arch Intern Med* 2005;165:1133–9.
14. Decroo T, Telfer B, Biot M, *et al*. Distribution of antiretroviral treatment through self-forming groups of patients in Tete province, Mozambique. *J Acquir Immune Defic Syndr* 2011;56:e39–44.
15. Kipp W, Konde-Lule J, Rubaale T, *et al*. Comparing antiretroviral treatment outcomes between a prospective community-based and hospital-based cohort of HIV patients in rural Uganda. *BMC Int Health Hum Rights* 2011;11(Suppl 2):S12.
16. Selke HM, Kimaiyo S, Sidle JE, *et al*. Task-shifting of antiretroviral delivery from health care workers to persons living with HIV/AIDS: clinical outcomes of a community-based program in Kenya. *J Acquir Immune Defic Syndr* 2010;55:483–90.
17. Callaghan M, Ford N, Schneider H. A systematic review of task-shifting for HIV treatment and care in Africa. *Hum Resour Health* 2010;8:8.
18. Laurant M, Reeves D, Hermens R, *et al*. Substitution of doctors by nurses in primary care. *Cochrane Database Syst Rev* 2005;CD001271.
19. Higgins JPT, Green S, eds. *Cochrane handbook for systematic reviews of interventions*. Wiley-Blackwell, 2008.
20. Cochrane Collaboration. A Cochrane Risk of Bias Assessment Tool: for Non-Randomized studied of Interventions (ACROBAT-NRSI). https://www.riskofbias.info/ (accessed 30 Oct 2015).
21. Egger M, Davey Smith G, Schneider M, *et al*. Bias in meta-analysis detected by a simple, graphical test. *BMJ* 1997;315:629–34.
22. Guyatt G, Oxman AD, Akl EA, *et al*. GRADE guidelines: 1. Introduction-GRADE evidence profiles and summary of findings tables. *J Clin Epidemiol* 2011;64:383–94.
23. Shamseer L, Moher D, Clarke M, *et al*; PRISMA-P Group. Preferred reporting items for systematic review and meta-analysis protocols (PRISMA-P). *BMJ* 2015;349:g7647.
24. Liberati A, Altman DG, Tetzlaff J, *et al*. The PRISMA Statement for reporting systematic reviews and meta-analyses of studies that evaluate healthcare interventions: explanation and elaboration. *BMJ* 2009;339:b2700.
25. Emanuel EJ, Wendler D, Killen J, *et al*. What makes clinical research in developing countries ethical? The benchmarks of ethical research. *J Infect Dis* 2004;189:930–7.

For peer review only

For peer review only - http://bmjopen.bmj.com/site/about/guidelines.xhtml

Downloaded from http://bmjopen.bmj.com/ on September 16, 2016 - Published by group.bmj.com

BMJ Open

The effects of shifting tasks from pharmacy to non-pharmacy personnel for providing antiretroviral therapy to people living with HIV: a systematic review protocol

Nyanyiwe M Mbeye, Tamara Kredo and Charles S Wiysonge

BMJ Open 2016 6:
doi: 10.1136/bmjopen-2015-008195

Updated information and services can be found at:
http://bmjopen.bmj.com/content/6/3/e008195

These include:

References This article cites 18 articles, 4 of which you can access for free at:
http://bmjopen.bmj.com/content/6/3/e008195#BIBL

Open Access This is an Open Access article distributed in accordance with the terms of the Creative Commons Attribution (CC BY 4.0) license, which permits others to distribute, remix, adapt and build upon this work, for commercial use, provided the original work is properly cited. See: http://creativecommons.org/licenses/by/4.0/

Email alerting service Receive free email alerts when new articles cite this article. Sign up in the box at the top right corner of the online article.

Topic Collections Articles on similar topics can be found in the following collections

Evidence based practice (557)
Health services research (1106)
HIV AIDS (159)

Notes

To request permissions go to:
http://group.bmj.com/group/rights-licensing/permissions

To order reprints go to:
http://journals.bmj.com/cgi/reprintform

To subscribe to BMJ go to:
http://group.bmj.com/subscribe/

For peer review only

S2. PRISMA Checklist

Section/topic	#	Checklist item	Reported on page #
**TITLE**			
Title	1	Identify the report as a systematic review, meta-analysis, or both.	1
**ABSTRACT**			
Structured summary	2	Provide a structured summary including, as applicable: background; objectives; data sources; study eligibility criteria, participants, and interventions; study appraisal and synthesis methods; results; limitations; conclusions and implications of key findings; systematic review registration number.	2-3
**INTRODUCTION**			
Rationale	3	Describe the rationale for the review in the context of what is already known.	7
Objectives	4	Provide an explicit statement of questions being addressed with reference to participants, interventions, comparisons, outcomes, and study design (PICOS).	8-9
**METHODS**			
Protocol and registration	5	Indicate if a review protocol exists, if and where it can be accessed (e.g., Web address), and, if available, provide registration information including registration number.	8
Eligibility criteria	6	Specify study characteristics (e.g., PICOS, length of follow-up) and report characteristics (e.g., years considered, language, publication status) used as criteria for eligibility, giving rationale.	8-9
Information sources	7	Describe all information sources (e.g., databases with dates of coverage, contact with study authors to identify additional studies) in the search and date last searched.	9
Search	8	Present full electronic search strategy for at least one database, including any limits used, such that it could be repeated.	10-11
Study selection	9	State the process for selecting studies (i.e., screening, eligibility, included in systematic review, and, if applicable, included in the meta-analysis).	12
Data collection process	10	Describe method of data extraction from reports (e.g., piloted forms, independently, in duplicate) and any processes for obtaining and confirming data from investigators.	12-13
Data items	11	List and define all variables for which data were sought (e.g., PICOS, funding sources) and any assumptions and simplifications made.	12-13
Risk of bias in individual studies	12	Describe methods used for assessing risk of bias of individual studies (including specification of whether this was done at the study or outcome level), and how this information is to be used in any data synthesis.	13
Summary measures	13	State the principal summary measures (e.g., risk ratio, difference in means).	13

For peer review only

S2. PRISMA Checklist

Synthesis of results	14	Describe the methods of handling data and combining results of studies, if done, including measures of consistency (e.g., I^2) for each meta-analysis.	13-15

Page 1 of 2

Section/topic	#	Checklist item	Reported on page #
Risk of bias across studies	15	Specify any assessment of risk of bias that may affect the cumulative evidence (e.g., publication bias, selective reporting within studies).	N/A
Additional analyses	16	Describe methods of additional analyses (e.g., sensitivity or subgroup analyses, meta-regression), if done, indicating which were pre-specified.	15
**RESULTS**			
Study selection	17	Give numbers of studies screened, assessed for eligibility, and included in the review, with reasons for exclusions at each stage, ideally with a flow diagram.	15
Study characteristics	18	For each study, present characteristics for which data were extracted (e.g., study size, PICOS, follow-up period) and provide the citations.	16-18
Risk of bias within studies	19	Present data on risk of bias of each study and, if available, any outcome level assessment (see item 12).	20-21
Results of individual studies	20	For all outcomes considered (benefits or harms), present, for each study: (a) simple summary data for each intervention group (b) effect estimates and confidence intervals, ideally with a forest plot.	21-24
Synthesis of results	21	Present results of each meta-analysis done, including confidence intervals and measures of consistency.	21-24
Risk of bias across studies	22	Present results of any assessment of risk of bias across studies (see Item 15).	21-24
Additional analysis	23	Give results of additional analyses, if done (e.g., sensitivity or subgroup analyses, meta-regression [see Item 16]).	n/a
**DISCUSSION**			
Summary of evidence	24	Summarize the main findings including the strength of evidence for each main outcome; consider their relevance to key groups (e.g., healthcare providers, users, and policy makers).	24-26
Limitations	25	Discuss limitations at study and outcome level (e.g., risk of bias), and at review-level (e.g., incomplete retrieval of identified research, reporting bias).	27-28
Conclusions	26	Provide a general interpretation of the results in the context of other evidence, and implications for future research.	27-28
**FUNDING**			
Funding	27	Describe sources of funding for the systematic review and other support (e.g., supply of data); role of funders for the systematic review.	28-29

For peer review only

S2. PRISMA Checklist

For peer review only
